# Plasma cells are enriched in localized prostate cancer in Black men and are associated with improved outcomes

**DOI:** 10.1038/s41467-021-21245-w

**Published:** 2021-02-10

**Authors:** Adam B. Weiner, Thiago Vidotto, Yang Liu, Adrianna A. Mendes, Daniela C. Salles, Farzana A. Faisal, Sanjana Murali, Matthew McFarlane, Eddie L. Imada, Xin Zhao, Ziwen Li, Elai Davicioni, Luigi Marchionni, Arul M. Chinnaiyan, Stephen J. Freedland, Daniel E. Spratt, Jennifer D. Wu, Tamara L. Lotan, Edward M. Schaeffer

**Affiliations:** 1 Department of Urology, Northwestern University Feinberg School of Medicine, Chicago, IL USA; 2 Department of Pathology, Johns Hopkins University School of Medicine, Baltimore, MD USA; 3 Decipher Biosciences, San Diego, CA USA; 4 Brady Urological Institute, Johns Hopkins University School of Medicine, Baltimore, MD USA; 5 Department of Radiation Oncology, University of Michigan, Ann Arbor, MI USA; 6 Department of Oncology, Johns Hopkins University School of Medicine, Baltimore, MD USA; 7 Department of Pathology, University of Michigan, Ann Arbor, MI USA; 8 Division of Urology, Department of Surgery, Cedars-Sinai Medical Center, Los Angeles, CA USA; 9 Division of Urology, Durham Veterans Affairs Health Care System, Durham, NC USA; 10 Department of Microbiology and Immunology, Northwestern University Feinberg School of Medicine, Chicago, IL USA

**Keywords:** Cancer microenvironment, Tumour biomarkers, Prostate cancer

## Abstract

Black men die more often of prostate cancer yet, interestingly, may derive greater survival benefits from immune-based treatment with sipuleucel-T. Since no signatures of immune-responsiveness exist for prostate cancer, we explored race-based immune-profiles to identify vulnerabilities. Here we show in multiple independent cohorts comprised of over 1,300 patient samples annotated with either self-identified race or genetic ancestry, prostate tumors from Black men or men of African ancestry have increases in plasma cell infiltrate and augmented markers of NK cell activity and IgG expression. These findings are associated with improved recurrence-free survival following surgery and nominate plasma cells as drivers of prostate cancer immune-responsiveness.

## Abstract (web summary)

A recent report suggested Black men with prostate cancer were more responsive to immunotherapy. Here, the authors analysed prostate cancer gene expression profiles and show tumours from Black men and men with African ancestry have an increased proportion of plasma cells compared to those of White men and this correlates with improved outcome following treatment.

## Introduction

Prostate cancer (PC) is the most common cancer and the second most common cause of cancer death among men in the United States^1^. Although PC is generally considered poorly immunogenic, some limited subsets experience durable responses to immunotherapy^2–4^. In one immunotherapy trial assessing sipuleucel-T, autologous dendritic cells sensitized to PC, the greatest responses were seen in self-identified Black men^4^. Analyses of PC from Black men based on African genetic ancestry have also demonstrated greater activation of immune-related signaling pathways^5^ but have not explored correlations between augmented tumor immune response as potential sensitivities to immunotherapy. Accordingly, we hypothesized the use of large-scale transcriptional profiling from a cohort of PC enriched with tumors from Black men or men of African ancestry could reveal clinically relevant differences in the tumor microenvironment (TME) immune infiltrate.

Here we show in two patient cohorts annotated with self-identified race and one patient cohort annotated with genetic ancestry comprised of over 1300 samples in total that PC from Black men or men of African ancestry (PC-B) have more infiltrating lymphocytes, which may be due to an increase in plasma cells and augmented interferon gamma (IFNG) signaling, IgG expression, and NK activity. We also note an increase in plasma cell content, which may be associated with prolonged recurrence-free survival following surgery for primary PC. These findings suggest that plasma cells could be potential biomarkers or targets for therapeutic response to immunotherapy for use in future prospective evaluation.

## Results

### Prostate tumors from Black men have increased immune content

**Fig. 1:**
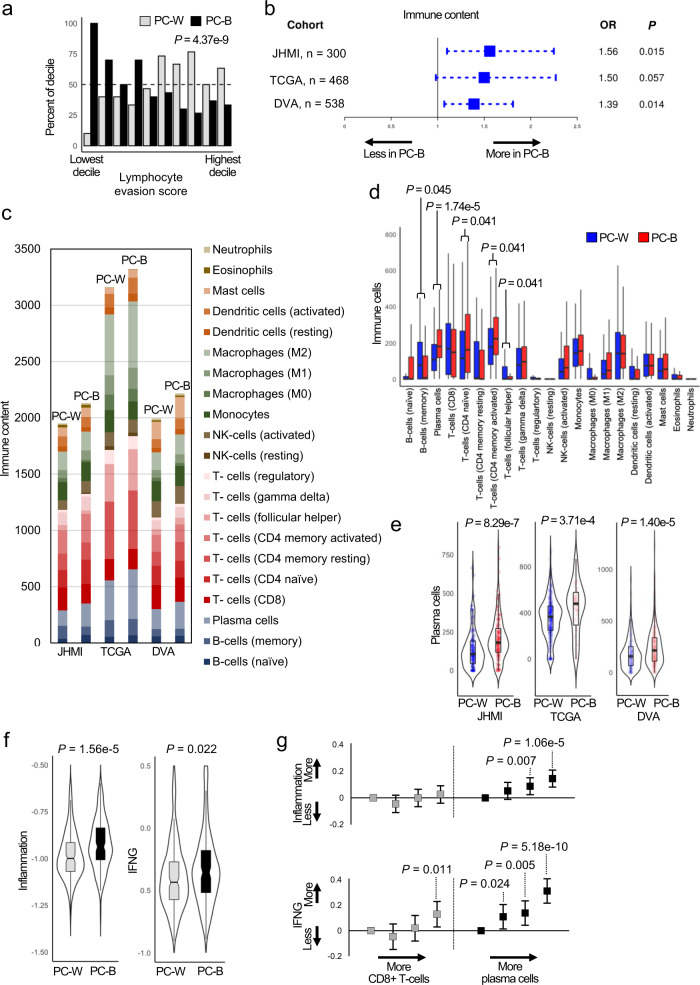
Prostate tumors from self-identified Black men or men of African genetic ancestry have increased quantities of plasma cells which is associated with increased immune activity. In JHMI, tumors with lower lymphocyte evasion scores were enriched with PC-B (**a**) suggesting PC-B is more susceptible to lymphocyte infiltration (Cochrane-Armitage test for trend). Accordingly, we note overall tumor immune content was higher in PC-B based on multivariable linear regression adjusting for tumor stromal content (**b**) (OR ± 95% CI). The immune content was deconvoluted and applied to our estimate of immune content represented by the different colorings of each bar in **c**. Within JHMI, the quantity of several cell types differed by race with increased plasma cells in PC-B representing the largest difference (**d**) (Wilcoxon Rank Sum FDR *P*-values; *n* = 300 patients). Of these, increased plasma cell content in PC-B was the only difference in both validation cohorts (Wilcoxon Rank Sum). Gene signatures of chronic and acute inflammation and IFNG were elevated in PC-B in JHMI (**f**) (Wilcoxon Rank Sum FDR *P*-values). From linear regressions, we demonstrate increasing quartiles of plasma cells were associated with greater expression of inflammation and IFNG but not for CD8 + T-cells (**g**) (estimate ± 95% CI; *n* = 300 patients; *p*-values not adjusted for multiple testing). Box plots **d**–**f**: center line, median; box limits, upper and lower quartiles; whiskers, 1.5x interquartile range. All *P*-values are two-sided. Abbreviations: JHMI Johns Hopkins Medical Institute, TCGA The Cancer Genome atlas, DVA Durham Veterans Affairs, PC-B prostate cancer from Black men, PC-W prostate cancer from White men, IFNG interferon gamma, OR odds ratio, CI confidence intervals, FDR false discovery rate.

Utilizing a discovery cohort of intermediate- and high-grade primary PC from radical prostatectomy specimens grade- and stage-matched by self-identified race (Johns Hopkins Medical Institute [JHMI]; PC-B = 150; White [PC-W] = 150; Supplementary Table 1) we assessed each tumor’s ability to resist lymphocyte infiltration using an established expression-based signature of tumor lymphocyte evasion (“Exclusion” score in Jiang et al.;^6^ See Methods). Tumors with the lowest lymphocyte evasion scores were significantly enriched among PC-B suggesting PC-B tumors are more susceptible to lymphocyte infiltration into the TME (Fig. 1a). Correspondingly, we found PC-B had higher levels of immune content based on the ESTIMATE signature (Fig. 1b)^7^. Similar trends were noticed in two additional (validation) cohorts: 1) The Cancer Genome Atlas (TCGA) which was annotated with genetic ancestry^8^ (PC-B = 58; PC-W = 410; Odds ratio=1.50, *P* = 0.057) and 2) a retrospective cohort of prostate tumors with whole transcriptomic data from radical prostatectomy at Durham Veterans Affairs Hospital (DVA) which was annotated with self-identified race (PC-B = 302; PC-W = 236; Odds ratio=1.39, *P* = 0.014; Supplementary Tables 2–3). After deconvoluting tumor invading lymphocytes by cell type (Fig. 1c), plasma cell content differed the greatest by self-identified race in JHMI and was the only cell type that also differed by genetic ancestry in TCGA and self-identified race in DVA with increased quantities in PC-B (Fig. 1d–e and Supplementary Tables 4–6). We also confirmed increases in B-lineage cells within PC-B using two orthogonal methods including a DNA methylation-based tool (see Methods; Supplementary Fig. 1a–b). To further validate these findings, we leveraged tumors from the previously described Kaur et al.^9^ cohort which showed PC-B based on self-identified race possessed increased signals of B-lineage cells based on DNA methylation signatures (Supplementary Fig. 1c) and both CD79a + and CD138 + cell density (Supplementary Fig. 1d–g).

**Fig. 2:**
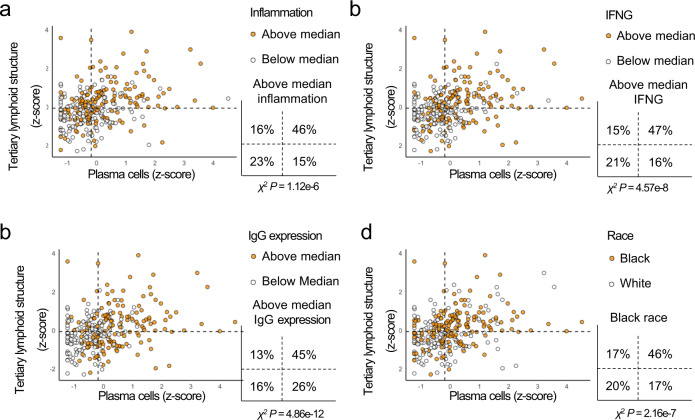
Intratumoral plasma cell and tertiary lymphoid structure content define a distinct subset of prostate cancer. Antigen presentation, cytokine-mediated signaling, and release of tumor-specific antibodies are increased when B-cells localize to tertiary lymphoid structures^17^. We show tumors with high tertiary lymphoid structure activity and plasma cells are enriched with high inflammation, IFNG, IgG expression, and self-identified Black race (**a**–**d**). Dashed horizontal and vertical lines indicate the median for tertiary lymphoid structure activity and plasma cells, respectively. *χ*^2^ *P*-values are all two-sided. Abbreviation: IFNG interferon gamma.

In a prospective trial of 19 men with advanced PC, tumors responsive to anti-CTLA-4 treatment were enriched with enhanced markers of inflammation and IFNG prior to treatment^10^. We noted increased signatures of both inflammation and IFNG activity in PC-B based on self-identified race in JHMI (Fig. 1f) and increased IFNG based on self-identified race in DVA and genetic ancestry in TCGA (Supplementary Fig. h). We also found increasing quantities of plasma cells had a continuous positive association with both increasing inflammation and *IFNG* expression while CD8^+^ T-cells, the immune cells more commonly thought to mediate the most tumor immune activity^11^, did not (Fig. 1g). In the setting of increased IFNG activity, IgG class-switch is augmented^12^. With this in mind, we would expect tumors with high plasma cell content to also have increased markers of IgG expression. We found IgG expression correlated highly with plasma cell content (Spearman’s correlation coefficient = 0.65) suggesting tumors with high plasma cell content also have high IgG expression, potentially as a reflection of plasma cell antibody secretion (Supplementary Fig. 1a–b). Additionally, plasma cell anti-tumor activity increases when they colocalize with other lymphocytes into organized cellular aggregates called tertiary lymphoid structures (TLSs)^13^. Thus, to further confirm the relationship between plasma cells and tumor immune activity, we measured TLS activity (see Methods). Accordingly, we noted tumors with high TLS activity and plasma cell content had higher levels of inflammation, *IFNG*, and IgG expression, and were enriched within PC-B based on self-identified race in JHMI (Fig. 2a–d). Immunohistochemical assessment of T-cells, B-cells, and plasma cells in tumors with high TLS activity confirmed the presence of discrete lymphoid aggregates relative to tumors with low TLS activity (Supplementary Fig. 1a–d). Thus, higher levels of plasma cells and TLSs define a subclass of tumors with higher immune activity which is more prevalent in tumors from Black men.

We also sampled a large cohort of men with metastatic castrate-resistant PC (mCRPC) annotated with self-identified race (PC-B = 7; PC-W = 102; Supplementary Table 7)^14^. No difference in plasma cell content by self-identified race was detected, which may be due to the low numbers of PC-B (Supplementary Fig. 4).

### Assessment of potential effect modifiers

We next assessed several potential sources of effect modification for our main finding of increased plasma cell content in PC-B. The association between PC-B and increased plasma cell content was independent of rearrangements in *ERG* and *PTEN* loss, two of the most common genomic alterations in PC which are less common in PC-B^5,15^, (Supplementary Fig. 5a–b and Supplementary Table 8). In a prospective trial assessing the immune-based therapy sipuleucel-T, Black men with PC experienced longer overall survival compared to White men, in particular when baseline serum prostate-specific antigen (PSA) was low^4^. Additionally, previous work has also noted enhanced immunoglobulin production in self-identified Black patients following vaccinations most notably at younger ages^16,17^. Here, the association between PC-B and increased plasma cell content was independent of baseline PSA and age (Supplementary Table 8). Finally, since the frequency of PC molecular subtypes differ by race^5^, we assessed the interaction between subtype and genetic ancestry on plasma cell content in TCGA. We found the association between PC-B and increased plasma cell content was significantly enhanced among tumors classified as “other” non-subtypeable tumors (Estimate of interaction +159.58, 95% CI 16.62 to 301.55, *P* = 0.028; Supplementary Table 8). Accordingly, of the 16 PC-B classified as “other” non-subtypeable PC, 14 (87.5%) were found to have above median plasma cell content (Supplementary Table 9). Among these 14 tumors, no single gene was amplified or deep deleted more than three times and no gene was mutated more than twice (Supplementary Data 1–2). Since, “other” non-subtypeable PC comprises about 26% of tumors in TCGA and PC-B is more likely to not be classified as “other” non-subtypeable PC^5,18^, these findings suggest a substantial portion of “other” non-subtypeable PC-B may be defined by their tumor immune microenvironment.

### Plasma cell content prognosticates outcomes following surgery

**Fig. 3:**
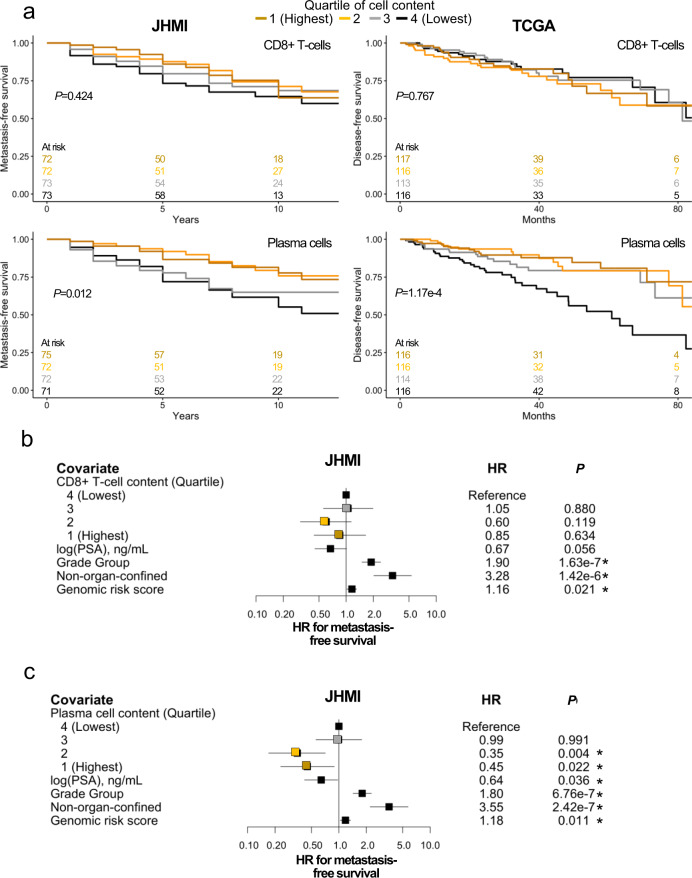
High intratumoral plasma cell content is associated with improved outcomes. Increasing plasma cell content is associated with longer disease-free survival while increasing CD8^+^ T-cell content is not (**a**) (log-rank two-sided *P*-values) even after adjusting for clinical covariates in Cox regressions (**b**–**c**) (*n* = 300 patients; HR ± 95% confidence interval; two-sided *P*-values). Asterisks indicate statistical significance with *p* < 0.05. Abbreviations: JHMI Johns Hopkins Medical Institute, TCGA The Cancer Genome atlas, HR Hazard ratio, PSA prostate-specific antigen.

Developing metastatic disease following local definitive treatment is a strong surrogate for overall survival for men with localized PC^19^. We found no association between metastasis-free and disease-free survival and CD8^+^ T-cell content. Conversely, increasing plasma cell content was associated with significantly prolonged metastasis-free and disease-free survival in JHMI and TCGA, respectively (Fig. 3a). In JHMI, plasma cell content and IgG expression did not tend to vary by grade, while in TCGA lower grade tumors tended to have higher plasma cell content (Supplementary Fig. 6a–b). In multivariable analysis including clinical covariates such as tumor grade, CD8^+^ T-cells remained non-significant while increased plasma cell content was independently associated with longer metastasis-free survival (Fig. 3b and Supplementary Fig. 2a–b).

**Fig. 4:**
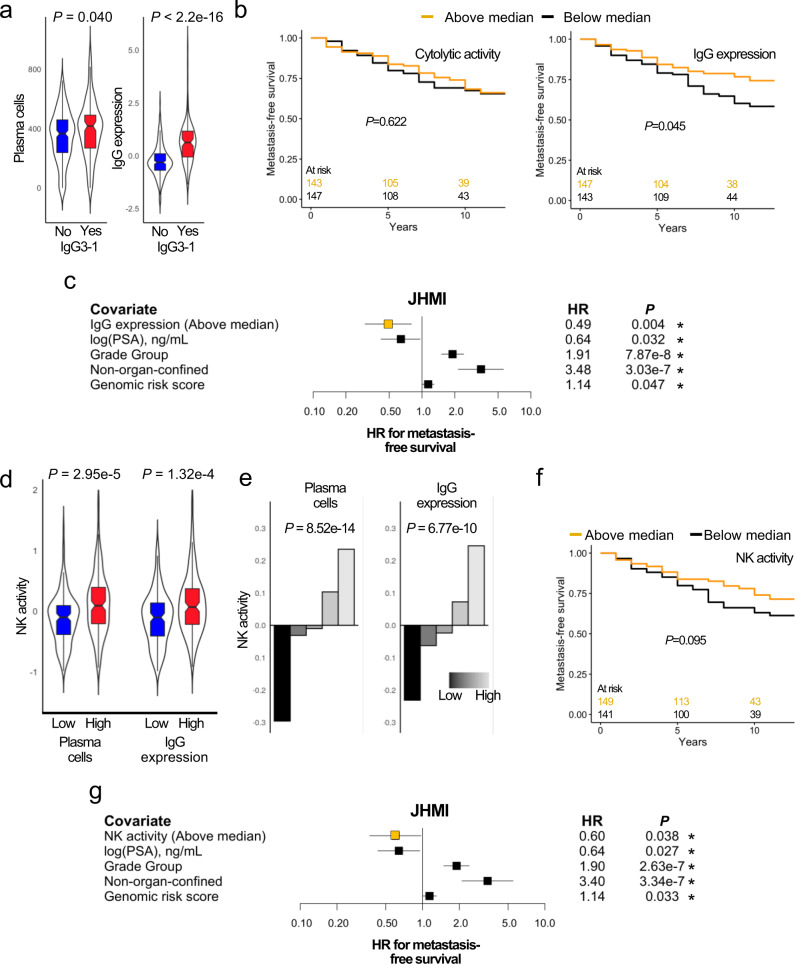
High IgG expression and NK activity are associated with improved outcomes. In TCGA, plasma cell content and IgG expression were both higher in tumors with any IgG3 to 1 subclass switch recombination, a measure of B-cell clonality (**a**); Wilcoxon Rank Sum; *n* = 361 patients). We also show while high T-cell activity as represented by cytolytic activity was not associated with metastasis-free survival in JHMI, high IgG expression was associated with longer survival duration (**b**) (log-rank) even after adjusting for clinical covariates in Cox regression (**c**) (*n* = 300 patients; HR ± 95% confidence interval). Because plasma cells and IgG contribute to antibody-dependent cellular cytotoxicity in term of tumor immunogenicity which is classically defined by NK cell activity in response to IgG^15,16^, we noted in JHMI NK activity is elevated in tumors with high levels of plasma cells and IgG expression (**d**) (Wilcoxon Rank Sum; *n* = 300 patients). Within DVA, increasing plasma cells and IgG expression showed a continuous relationship between these scores and NK activity (**e**) (Kruskal–Wallis analyses for trend). Higher NK activity in JMHI trended towards an association with longer time to metastasis (**f**) (log-rank) which reached significance after adjusting for clinical covariates in Cox regression (**g**) (*n* = 300; HR ± 95% confidence interval). All survival estimations were performed using the Kaplan–Meier method. Asterisks next to *p*-values from Cox regressions denote statistical significance with *p* < 0.05. Box plots (**a**, **d**): center line, median; box limits, upper and lower quartiles; whiskers, 1.5x interquartile range. All *P*-values are two-sided. Abbreviations: JHMI Johns Hopkins Medical Institute, TCGA The Cancer Genome atlas, DVA Durham Veterans Affairs, HR Hazard ratio, PSA prostate-specific antigen.

When B cells encounter a foreign antigen, they undergo clonal expansion and immunoglobulin class-switch recombination. The most common class-switch recombination noted in multiple tumors types is IgG3 to 1 which is associated with cancer outcomes and can be measured by a signature originally described in an analysis by Hu et al.^20^. In tumors from TCGA with any IgG3 to 1 class-switch recombination there were greater quantities of plasma cell content, more IgG expression, and a greater prevalence among tumor from men of African genetic ancestry (Fig. 4a and Supplementary Fig. 8). This observation suggests increasing plasma cell content and IgG expression may be reflective of B-cell antigen recognition within the TME. Accordingly, high IgG expression was associated with longer metastasis-free survival while high cytolytic activity, a measure of CD8^+^ T-cell activity (see Methods) was not (Fig. 4b–c). Tumor immune activity associated with plasma cell activity and IgG expression is typically mediated via antibody-dependent cellular cytotoxicity (ADCC) driven primarily by NK cell activation^21,22^. Accordingly, we noted increased NK activity in tumors with high levels of plasma cells and IgG expression (Fig. 4d–e). Plasma cell content, IgG expression, and NK activity were all independent of tumor neoantigen burden and fraction genome altered in TCGA consistent with previous work on tumor plasma cell content and TLS in melanoma and sarcoma (Supplementary Fig. 9a–b)^10,23^. Similarly, neoantigen burden and fraction genome altered did not correlate with race (Supplementary Fig. 9c–d), In the JHMI cohort, high NK activity was associated with longer duration to metastatic disease following radical prostatectomy (Fig. 4f–g). These data suggest future studies are indicated to assess the role of plasma cell-activated ADCC mediated by NK activity in tumor progression for primary PC.

Previous work from Shalapour et al. demonstrated 138 + /IgA+ positive tumors were associated with immune-related treatment resistance and characterized how immunosuppressive plasma cells can impeded an anti-tumor T-cell response^24^. Thus, findings from the current work, other previous work^10,23,25^, and that of Shalapour et al.^24^ suggest B-cells in the prostate TME are heterogeneous in terms of their protumorgenic or antitumorgenic roles^26^. One consideration to reconcile the potential heterogeneous roles of B cells may be related to the inflammatory cytokine milieu in the TME. Antibody isotype switch is driven by different local inflammatory cytokines in the microenvironment. For example, IgA is mostly found in the intestines, predominantly driven by immune suppressive cytokine such as iNOS and IL-10^27,28^ whereas switch to IgG is mostly driven by IFNG^29,30^.

As noted above, plasma cell content was positively associated with IFNG in primary PC (Fig. 1g). Conversely, plasma cell content was not strongly correlated with the expression of several relevant inflammatory factors that induce IgA class switch (Supplementary Fig. 9e)^27^. Thus, discrepancies in the TME inflammatory cytokine milieu leading to differential Ig isotype switching may account for the heterogeneous observed associations between plasma cell and PC outcomes.

## Discussion

Tumor immune activity and prognosis are often associated with the presence and activity of CD8^+^ T cells in the TME^3,11^. However, it is unclear in PC if CD8^+^ T cells play a predominate role early in the tumor immune response and delaying progression^31^. Recent evaluations of immune activity in multiple tumor types have suggested B cells and plasma cells in the TME are significant biomarkers for predicting immunotherapy responsiveness^10,23,25^. For men with PC receiving immunotherapy in the form of sipuleucel-T, treatment response correlates with induced elevated serum levels of anti-tumor IgG and, interestingly, longer survival was noted in self-identified Black men^4,32^. In multiple independent cohorts comprised of over 1300 PC tumors including two cohorts with 452 tumors from self-identified Black men and one cohort with 58 tumors from men of African genetic ancestry, we show primary PC from Black men or men of African ancestry possess increased quantities of plasma cells and IgG expression which also define a subset of tumors with enhanced innate freedom from disease progression. These tumors demonstrated increased levels of NK cell activity suggesting plasma cells may augment ADCC within the TME, a mechanism that warrants future validation. Together, these findings imply B cells and plasma cells in the TME may play an important role in prostate tumor immune response. Assessing intratumoral B cells and plasma cells may help identify a pathway to engage the immune system for PC treatment and nominate these cell types and various measurements of the plasma cell activity or presences in the TME such as the transcriptome-based signatures, methylation-based signatures, or immunohistochemistry methods used in this work as biomarkers to predict responsiveness to immunotherapy (Supplementary Table 10).

Notably, a relationship between disease recurrence and plasma cell content was distinctly non-linear (Fig. 3a–b) which should encourage future work determining a manner to measure and ascertain a clinically relevant cutoff for plasma cell content. Additionally, extending these observations to the mCRPC disease state are limited as there were only seven mCRPC annotated as self-identified PC-B which is a common issue in race-based genomic analyses^33^. We support further evaluation of advanced PC within self-identified Black men and those of African genetic ancestry to validate our observation in primary PC.

Our data suggest the prognostic value of plasma cell content in the TME may be defined by the context of inflammatory cytokine milieu. These observations warrant experimental designs to identify and potentially induce the TME context needed to optimize any plasma cell antitumorgenic effects. Since plasma cells were enriched in tumors from self-identified Black men and men of African genetic ancestry, leveraging these observations could help reduce racial disparities in PC outcomes^34^.

## Methods

### Patients cohorts and tissue

We derived three cohorts of PC tumors to first discover varying immunogenic signatures by Black race or African ancestry then validate these findings. The discovery cohort was comprised of two groups of patients annotated with self-identified race who underwent radical prostatectomy and no additional treatment until metastatic recurrence at Johns Hopkins Medical Institute (JHMI). The first group consisted of 355 intermediate- or high-risk patients treated between 1995 and 2005, of which 33 were self-identified as Black^35^. The second group consisted 143 self-identified Black men treated from 2006 to 2010^9^ for a total of 312 self-identified White men and 176 self-identified Black men. To prepare for matching, 6 (1%) men were excluded due to missing data on pathological stage (4 self-identified White men and 2 self-identified Black men). All men with grade group 1 disease were excluded given the disproportionate numbers among self-identified Black men (24 vs 6; 30 total, 6%). Additionally, excluding men with grade group 1 PC would create a cohort more ideal for our analyses of outcomes following radical prostatectomy since the preferred treatment for most men with low grade PC is active surveillance with deferred treatment for signs of disease progression^36^. Finally, the cohort was sorted using a random list of numbers generated by the “sample” function from the “base” R package before using the “matchit” function from the “Matchit” version 3.0.2 R package to derive a final cohort comprised of all 150 self-identified Black men grade- and stage-matched to 150 self-identified White men.

Our first validation cohort was generated from publicly available data within TCGA PRAD dataset (*n* = 468).This cohort was annotated with genetic ancestry as available at the Cancer Genetic Ancestry Atlas (http://52.25.87.215/TCGAA/; 410 European ancestry and 58 African ancestry)^8^. Expression, DNA methylation, and clinical data from TCGA were downloaded via cBioPortal (https://www.cbioportal.org/study/summary?id=prad_tcga)^37,38^. Pre-operative prostate-specific antigen levels for TCGA were downloaded from the Broad Institute TCGA Genome Data Analysis Center^39^. Because there were so few men of African genetic ancestry represented in TCGA we also considered general trends in TCGA as validation for findings based on self-identified race from JHMI. To further confirm findings from JHMI, we used a second, large retrospective cohort with whole transcriptomic data (DVA; *n* = 538)^40^. This cohort was annotated with self-identified race (236 self-identified White and 302 self-identified Black). This group was comprised of genome-wide expression profiles from formalin-fixed paraffin-embedded prostate tumors from radical prostatectomy between 1989 and 2016. Investigation in our discovery cohort were exploratory and all analyses in the validation cohorts were confirmatory. Characteristics of our discovery and validation cohorts can be seen in Supplementary Tables 1–3. A description of the strength and limitations of these three main cohorts are provided in the Supplementary Methods. For JMHI and DVA, Institutional Review Board approval was provided by those respective institutions and for TCGA, individual contributing sites were responsible for ethical oversight, including informed consent. Thus, this work complies with all relevant ethical considerations.

Following the discovery and validation of our main findings regarding increased quantities of B-lineage and plasma cells in PC-B, we leveraged a fourth cohort for DNA-methylation-based and histologic validation. This cohort consisted of 135 prostate tumors from self-identified Black men grade-matched to 135 prostate tumors from self-identified White men who underwent radical prostatectomy. Complete description of this cohort can be found in Kaur et al.^9^. Measurements from tumors were from distinct samples without any repeated measurements. Finally, data from biopsies of 118 mCRPC tumors were downloaded from cBioPortal (https://www.cbioportal.org/study/summary?id=prad_su2c_2015)^14,37,38^. From the authors of the data behind this cohort, self-identified race was provided for 109 patients (Supplementary Table 7).

### Gene expression profiling and genomic data

Expression profiling for tumors from JHMI and the DVA were conducted in a Clinical Laboratory Improvement Amendments (CLIA)-certified laboratory facility (Decipher Biosciences, San Diego, CA, USA)^41^. All tumors underwent central pathology review and at least 0.5 mm^2^ of tumor with ≥60% tumor cellularity were required for sampling. RNeasy FFPE (Qiagen, Valencia, CA) was used for RNA extraction and purification. Ovation WTA FFPE system (NuGen, San Carlos, CA) was used for amplification and labeling of RNA, which was then hybridized to Human Exon 1.0 ST GeneChips (Thermo-Fisher, Carlsbad, CA). Affymetrix Power Tools were used for quality control. Finally, the Single Channel Array Normalization (SCAN) algorithm (“SCAN” function from “SCAN.UPC” version 2.32.0 R package) was used for normalization^42^. TCGA and mCRPC expression data were quantile matched to the microarray platform such that all signatures developed on the microarray platform could be applied. All genomic and expression data for TCGA and mCRPC was acquired from cBioPortal^37,38^.

### Tumor immune cell content

Tumor immune and stromal content were calculated based on RNA expression using methods from Yoshihara et al. using the “ESTIMATE” R package version 2.0.0^7^. Our final immune content score was the derived immune score plus the constant of the minimum score in each cohort plus one. This was done so that when we would apply the proportions of the deconvolution of tumor-invading lymphocytes the final quantities of tumor-invading lymphocytes would be positive. Tumor-invading lymphocytes were deconvoluted using the MySort tool (default version) implemented in a R function^43,44^. This produced a proportion of 21 different immune cells within each tumor, which was multiplied by the estimated immune content to derive a quantity of each cell type that could be compared between samples within the same cohort. A second RNA expression-based method for immune cell deconvolution, Microenvironment Cell Populations-counter (MCP-counter) version 1.1.0, was applied to confirm findings from MySort^45^.

As an orthogonal approach to deconvolute the immune TME, we also applied the DNA methylation-based tool methyCIBERSORT version 0.2.1 to TCGA and Kaur et al.^9,46^. The whole methylomes of these cohorts were investigated through the Infinium MethylationEPIC array platform (illumina). The “minfi” version 1.36.0 package in R was used to determine the quality of methylation experiments and to derive single probe scores per tumor sample. For methylCIBERSORT analysis, raw probe scores were normalized using the “preprocessNoob” function in “minfi.”

### ERG + /- and PTEN loss signatures

A validated transcriptome-based signature with 95% accuracy for predicting *ERG* + */-* status as described by Tomlins et al. was applied to JHMI and DVA to assess the interaction between *ERG* + */-* and race and race-based differences in the TME^47^. To the DVA tumors, an expression-based signature predictive of *PTEN* loss was applied as well^48^.

### Tertiary lymphoid structure signature

Using a list of tertiary lymphoid structure-hallmark genes^23,49^, a signature to reflect the activity and presence of tertiary lymphoid structures within the TME was generated as the geometric mean expression of seven genes (*CCR7*, *CXCR5*, *SELL*, *LAMP3*, *CCL19*, *CCL21*, and *CXCL13*).

### Immunohistochemistry and image analysis

Immunohistochemistry for CD79a, a pan-B-cell membrane protein^50^, and CD138/syndecan-1, a marker of plasma cells, within the Kaur et al. cohort was conducted using mouse monoclonal antibodies (JCB117, CellMarque, pre-dilute and B-A38, Ventana/Roche, pre-dilute, respectively) on the Ventana Benchmark platform in a CLIA-accredited laboratory. For CD79a, cell densities were determined using QuPath version 0.1.2^51^ digital image analysis algorithms to count the number of CD79a + cells within each 0.6 mm diameter tissue microarray punch of tumor (average of four spots sampled per case), normalized by the total mm^2^ of tissue analyzed and taking the mean across the four tumor cores (biological replicates) for each patient. In the case of CD138, we observed patchy expression of *CD138* in the benign prostate epithelium (predominantly basal cells) as well as in a small subset of tumor cells, making automated digital image quantification of CD138 + cells impossible (see arrowhead in Supplementary Fig. 1e). This expression of *syndecan-1* in prostate epithelial cells has been previously published^52^. To circumvent this issue, we performed a manual quantification of CD138 + cell density blinded to all clinical and patient characteristics, counting all individual cells positive for CD138 in each 0.6 mm diameter tissue core of tumors (average of four spots sampled per case), normalized by the total mm^2^ of tissue analyzed for each core calculated in QuPath version 0.1.2 as above and taking the mean across the four tumor cores for each patient. All attempts at biological replicates were successful.

As a qualitative means of assessing the expression-based signature for TLS, five tumors with high TLS signature scores (highest pentile) were randomly chosen and immunostained for CD3 (Rabbit polyclonal, A0452, Dako, 1:100 dilution), CD20 (mouse monoclonal, L26, Ventana/Roch, pre-dilute), and CD138 (Mouse monoclonal, B-A38, Ventana/Roche, pre-dilute) on adjacent slides using the Ventana Benchmark system in a CLIA-accredited lab. These images were merged and pseudocolored to demonstrate the spatial relational between cell types. In each, discrete lymphoid aggregates of T and B cells, with scattered adjacent CD138 + cells, are demonstrated suggesting the presence of TLS (Supplementary Fig. 3b–d). Three tumors in the lowest pentile of TLS signature scores were also randomly chosen and immunostained for CD20 and CD3 to qualitatively demonstrate a lack of lymphoid aggregates relative to high TLS tumors.

*PTEN* status in JHMI samples were based on immunohistochemistry using a previously validated technique^53^. Briefly, tissue micro-array samples were incubated with rabbit antihuman PTEN antibody (Clone D4.3 XP, Cell Signaling, 1:100 dilution) for 36 min at 36 °C temperature using the Ventana Benchmark system in a CLIA-accredited lab. Using a NanoZoomer XR slide scanner (Hamamastu), stained tissue micro-arrays were scanned at 20 x magnification. PTEN status was then determined based on the evaluation of two uropathologists who were blinded to tumor grade and clinical outcomes. Tumors with complete loss of immunostaining compared to the internal control benign glands and stroma were considered positive.

### Markers of tumor immune susceptibility and activity

We derived a tumor lymphocyte evasion score as defined by the expression of genes from cell types that are capable of evading lymphocyte infiltration (cancer-associated fibroblasts [CAFs], myeloid-derived suppressor cells [MDSCs], and M2 subtype of tumor-associated macrophages [TAMs]) as described by Jiang et al. (“exclusion” score in previous publication; web application for TIDE [default version]: http://tide.dfci.harvard.edu/)^6^. From the Hallmark Gene Set Collection, we also derived a measure of genes upregulated during chronic and acute inflammation (HALLMARK_INFLAMMATORY_RESPONSE) and interferon gamma (IFNG; HALLMARK_INTERFERON_GAMMA_RESPONSE)^54^. Increased expression of this IFNG signature was previously shown to be enriched in tumors that responded to anti-CTLA-4 systemic therapy in men with advanced PC^3^.

Within TCGA, neoantigen burden data were downloaded from The Cancer Immune Atlas project (https://tcia.at/) which was calculated using the OptiType version 1.2 pipeline^55^. Data were available for 420 of the 468 in our final cohort. Fraction genome altered was defined as the length of segments with log2 or linear copy number alteration value larger than 0.2 divided by the length of all segments measured as available in cBioPortal^37,38^.

### IgG expression metagene

We applied unsupervised hierarchal clustering to a gene-set of proteins related to IgG components and expression originally assessed in breast cancer^56^. We found six IgG genes were highly correlated with each other in PC tumors from JHMI (*IGLC2*, *IGKC*, *IGHG1*, *IGHA1*, *IGHM*, and *IGHG3*; Supplementary Fig. 2a), each of which were independent of the signature matrix used for immune cell deconvolution signature matrix used with MySort. The geometric mean expression of these genes comprised our IgG expression metagene, a measure of plasma cell activity.

### T-cell and NK activity signatures

Cytotoxic T-cell (CD8^+^) activity was measured as the geometric mean expression of *GZMA* and *PRF1* as described by Rooney et al.^57^. Because NK cells have overlapping gene expression with CD8^+^ T cells, we sought to derive a metagene more specific to NK cells activation. As described by Cursons et al., we represented NK activity as the geometric mean expression of eight genes expressed during NK activation (*IL15*, *XCL1*, *XCL2*, *CCL5*, *FLT3LG*, *GZMA*, *GZMB*, and *FASLG*)^58^.

### Clinical outcome measurements

Patients from JHMI were followed until the development of metastatic disease following radical prostatectomy. Notably, in this cohort, no patients received any adjuvant treatment following surgery. Metastatic disease development was assessed with imaging using computed tomography or bone scan following biochemical recurrence, as defined by a rise in PSA > 0.2 ng/mL. In TCGA, patients were followed until any disease recurrence which included the earlier of development of metastatic disease as well as any biochemical recurrence. Included in multivariable analyses for metastasis-free survival was a validated genomic risk score for developing metastatic disease following treatment for localized prostatectomy based on transcriptomic signatures^59^. In JHMI, 72 (24%) men developed metastatic disease and in TCGA 82 (17.5%) developed disease recurrence. This study complied with REMARK reporting guidelines for prognostic tumor biomarkers^60^.

### Statistical analyses

All statistical tests were conducted using RStudio version 1.2.5019 (Boston, MA) and only two-sided *p*-values were generated. Patient characteristics were compared using *χ*^2^ to compare categorical variables and Student’s *t*-test to compare age and PSA. Because stromal content and immune content in tumors positively correlate with each other and thus high levels of immune content may reflect high levels of stroma^7^, when comparing immune content based on race, we performed a logistic regression adjusting for stromal content with Black race as the outcome variable. Individual immune cell type quantities were compared by race within our discovery cohort (JHMI) using the Wilcoxon rank-sum test, and similar comparisons were made in the first validation cohort (TCGA) with cell types that differed with a false-discovery rate (FDR) *P* < 0.05. Parallel findings in TCGA were confirmed in our second validation cohort (DVA). Subsequent comparisons of continuous variables between two groups were assessed using the Wilcoxon rank-sum test unless otherwise noted. Spearman’s correlation was used for correlation analyses. Continuous variables were categorized into equal quantiles for various comparisons. High vs low categorizations of continuous variables were divided by above vs below median unless otherwise noted. Kaplan–Meier analyses were used to estimate metastasis-free survival in the JHMI cohort and disease-free survival in TCGA. Log-rank analyses were used for univariable survival analyses while Cox proportional hazards models were used for multivariable analyses adjusting for clinical covariates. In multivariable Cox regressions, PSA was assessed as a log-transformed continuous variable. Grade group was assessed as a continuous variable per increase in 1 group from 1 (low grade) to 5 (high grade). T-stage was assessed as a categorical variable defined as organ confined as pT1-2 and N0 and non-organ-confined as all others. Genomic risk score was defined as a transcriptomic-based validated score with higher values predicting increased risk of disease recurrence following local treatment for PC and was assessed as a continuous variable per 0.1 increase (range of possible scores: 0.0 to 1.0)^59^.

### Reporting summary

Further information on research design is available in the Nature Research Reporting Summary linked to this article.

## Supplementary information

Supplementary Information

Peer Review File

Description of Additional Supplementary Files

Supplementary Data 1

Supplementary Data 2

Reporting Summary

## Data Availability

The data that support the findings of this study from the Johns Hopkins Medical Institute and Durham Veterans Affairs Hospital were deposited on NCBI Gene Expression Omnibus (GEO) and are accessible through GEO Series accession number GSE153352 and GSE157547, respectively. The data for TCGA and the mCRPC cohort can be downloaded at cBioPortal at [https://www.cbioportal.org/study/summary?id=prad_tcga] and [https://www.cbioportal.org/study/summary?id=prad_su2c_2015], respectively. The remaining data are available within the Article, Supplementary Information or available from the authors upon request. Source data are provided with this paper.

## Source data

Source Data
