# Supplementary material for: Plasma cells are enriched in localized prostate cancer in Black men and are associated with improved outcomes
Source: Nat Commun. 2021 Feb 10;12:935. doi: 10.1038/s41467-021-21245-w (PMC7876147; doi:10.1038/s41467-021-21245-w)
Supplement: Supplementary file 1 — Supplementary Information [file 41467_2021_21245_MOESM1_ESM.pdf]

Supplementary information

Plasma cells are enriched in localized prostate cancer in Black men and are associated with improved outcomes

Weiner *et al.*

Supplementary figures

Supplementary figure 1: Orthogonal methods confirm the association between self-identified Black race or African genetic ancestry and increased plasma cell content

Supplementary figure 2: A signature for IgG expression positively correlates with plasma cell content

Supplementary figure 3: Immunohistochemistry validation of the tertiary lymphoid structure expression signature

Supplementary figure 4: Plasm cell content by self-identified race in metastatic castrate resistant prostate cancer

Supplementary figure 5: The association between self-identified Black race and increased plasma cell content is independent of alterations in *ERG*

Supplementary figure 6: Tumor plasma cell content and IgG expression stratified by grade group

Supplementary figure 7: Multivariable Cox regression for disease-free survival in TCGA

Supplementary figure 8: IgG3 to 1 subclass switch recombination by genetic ancestry in The Cancer Genome Atlas

Supplementary figure 9: Signature correlations with mutation burden and inflammatory cytokines

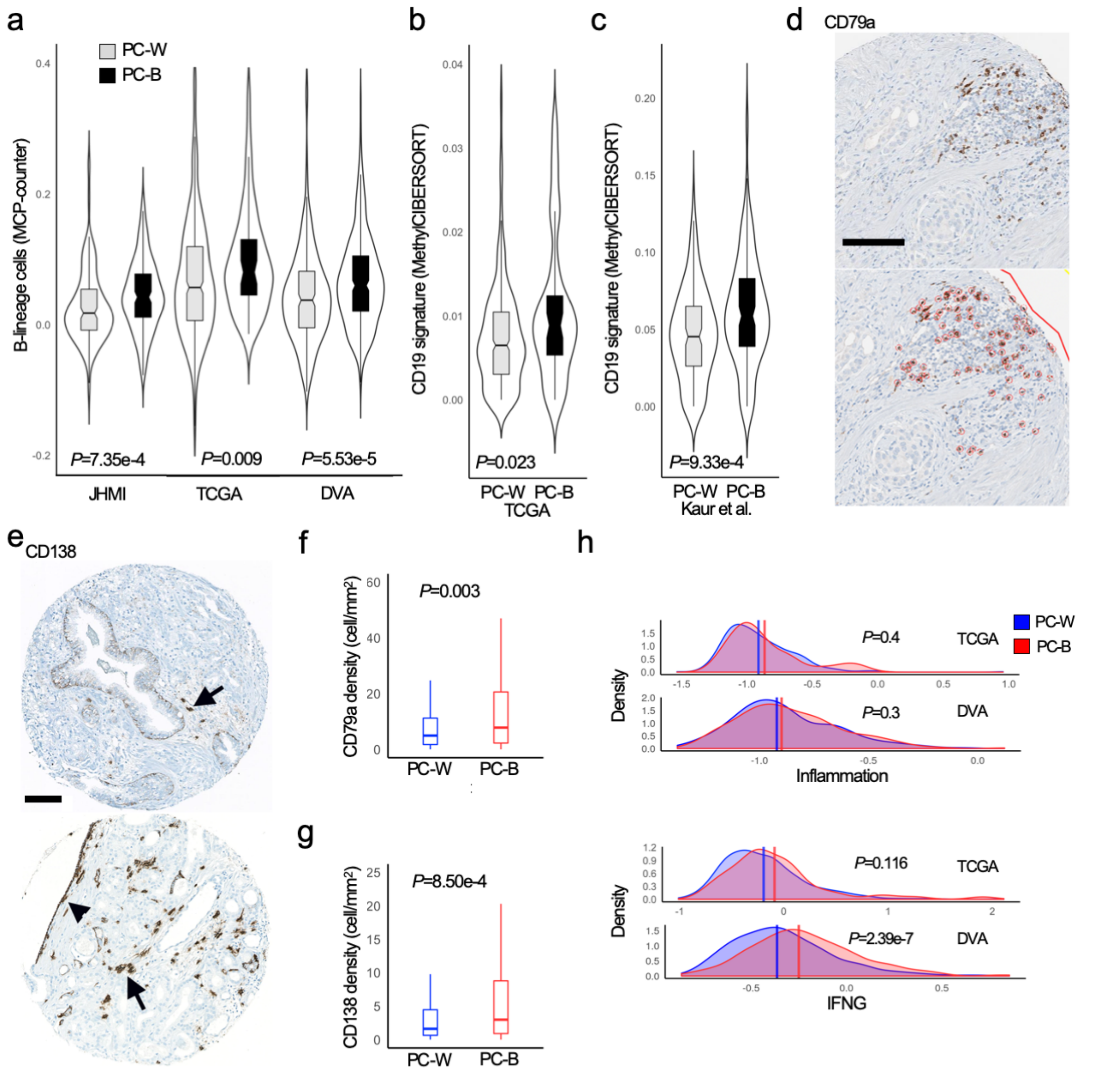

Supplementary figure 1: Orthogonal methods confirm the association between self-identified Black race or African genetic ancestry and increased plasma cell content. A second method for immune cell deconvolution, Microenvironment Cell Populations-counter (MCP-counter), was applied to confirm findings from MySort (**a**; two-sided *P*-values based on Wilcoxon Rank Sum; JHMI, n=300 patients; TCGA, n=468 patients; DVA, n=548 patients).[1,2] Using DNA-methylation-based signatures[3] in TCGA and a grade-matched cohort of 135 PC-B and 135 PC-W based on self-identified race (Kaur *et al.* 2019)[4] B-cell (CD19) lineage signals were again higher in PC-B tumors (**b-c**; two-sided *P*-values based on Wilcoxon Rank Sum). Representative CD79a immunostaining from one of 270 tumors in a tumor from a Black patient in the Kaur et al. cohort shows abundant positive cells (brown; **d**, top panel; scale bar 100µm). Automated digital image cell segmentation without replicates identifies the density of positive cells per mm^2 of tissue sampled (red circles; **d**, bottom panel). Representative images of CD138 immunostaining from one of 270 tumors in prostate cancer tissue microarray cores show cases with high levels of CD138+ plasma cells (**e;** arrows; scale bar 100µm). Manual quantification without replicates was required due to patchy CD138 immunopositivity in background benign epithelial cells (arrowhead). All images reduced from 200x magnification. CD79a+ and CD138+

densities were higher in PC-B (**f-g**; n=270 patients; two-sided *P*-values based on Wilcoxon Rank Sum). Similar to results from JHMI (Figure 1f), trends toward greater levels of inflammation and IFNG were noted in PC-B in TCGA and DVA (**h**; two-sided *P*-values based on Wilcoxon Rank Sum). 3 Abbreviations: JHMI, Johns Hopkins Medical Institute; TCGA, The Cancer Genome atlas; DVA, Durham Veterans Affairs; PC-B, Prostate cancer from men of self-identified Black race or African genetic ancestry; PC-W, prostate cancer from men of self-identified White race or European genetic ancestry; IFNG, interferon gamma.

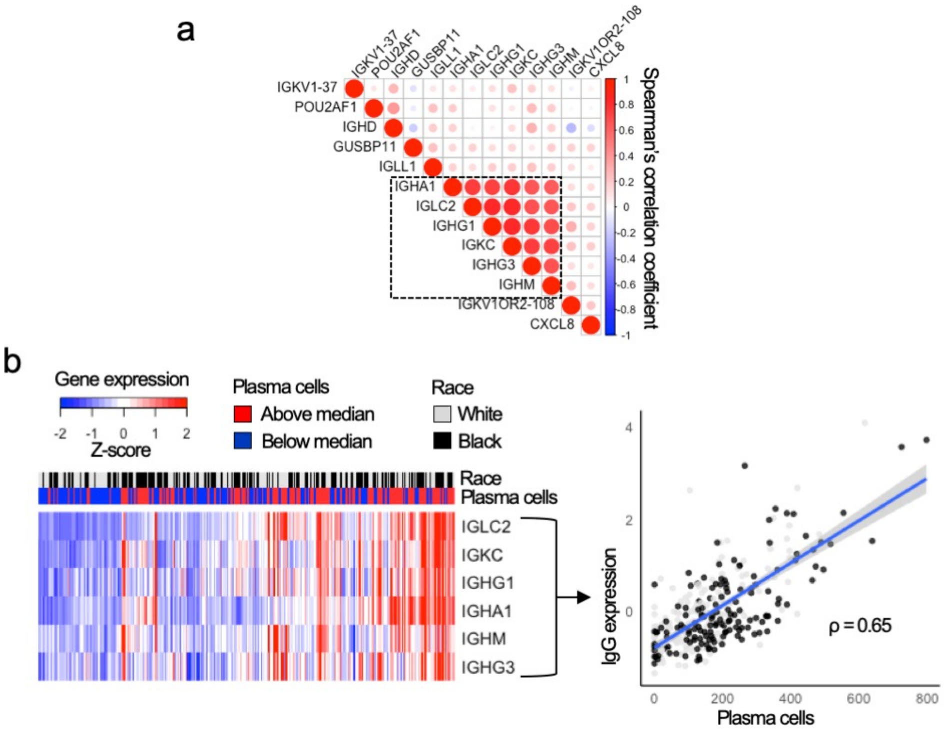

Supplementary figure 2: A signature for IgG expression positively correlates with plasma cell content. Because plasma cell IgG production is augmented in the presence of IFNG expression and tertiary lymphoid structures (TLS), and both IFNG expression, TLS signatures, and plasma cells are present to greater extents in prostate cancer from Black men, we sought to derive a measure of IgG expression. We performed hierarchical clustering in Johns Hopkins Medical Institute (n=300 patients annotated with self-identified race) using an IgG gene set from Rody *et al*. originally designed for breast cancer. Six genes in this gene set tended to cluster within prostate tumors (**a**) and were ultimately included in our IgG signature. From the geometric mean expression of these genes, we created an IgG expression signature which correlated well with plasma cell content (**b**; Spearman's correlation coefficient rho shown with two-sided p<2.2e-16; shading represents 95% confidence level interval for predictions from a linear model).

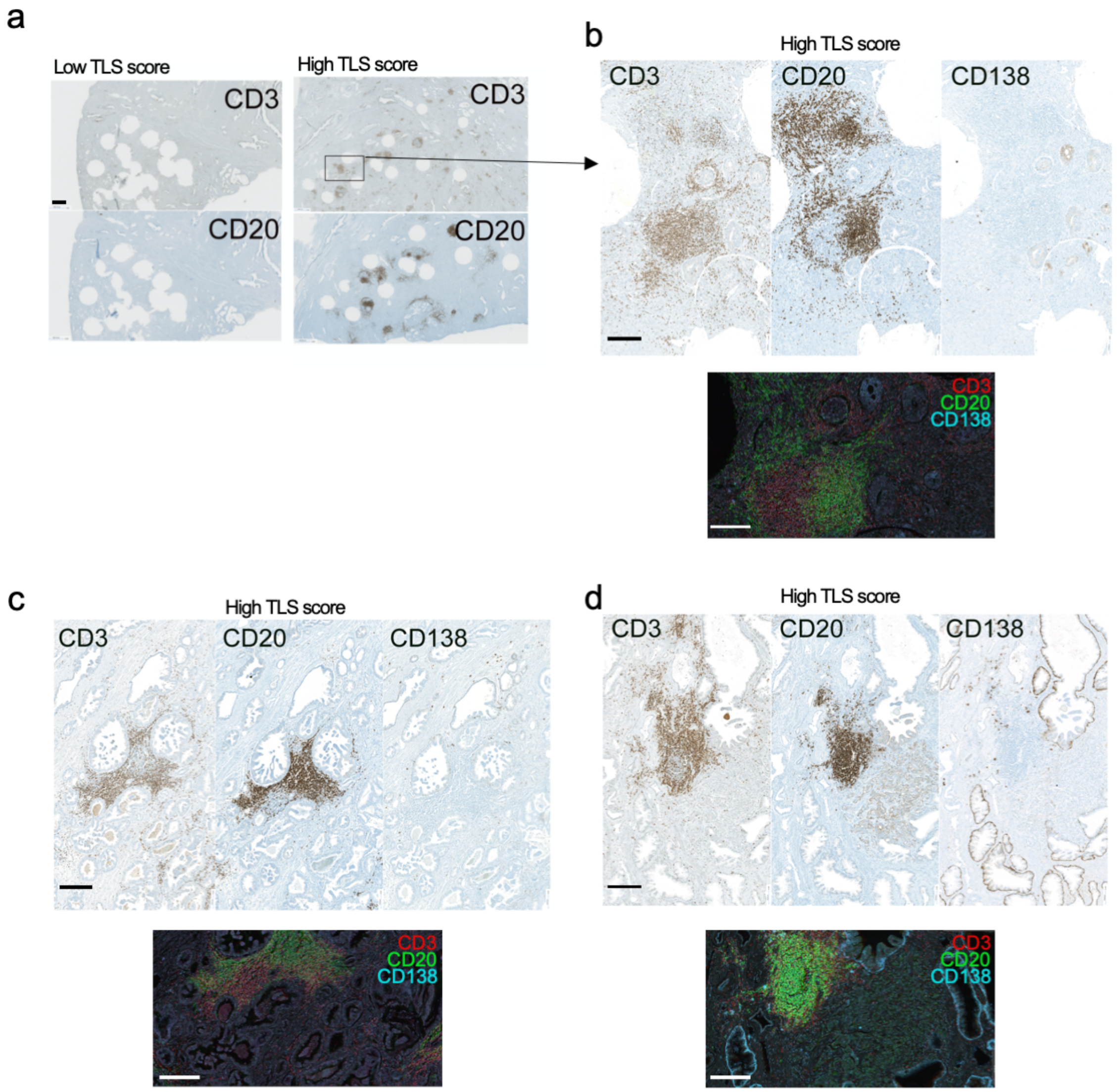

Supplementary figure 3: Immunohistochemistry validation of the tertiary lymphoid structure expression signature. Representative images from one of four tumors from the highest and one of three tumors from the lowest pentile of TLS signature scores which were randomly selected for immunostaining (**a**; scale bar 500μm). At low magnification, discrete lymphoid aggregates can be seen in the tumor with the high TLS score surrounding the areas of tissue microarray tumor punch samplings. The examples of low and high TLS score tumors were similar to the results of two others with low scores and three others with high scores. The box in the upper panel of the high TLS score tumor represents the area magnified in (**b**). Shown are three of the four randomly selected tumors in the highest pentile of TLS scores that underwent immunostaining for CD20, CD3, and CD138 on adjacent slides (**b-d**; top panels; scale bars 200μm) which were then merged and pseudocolored (lower panels with same orientation as top panels; top panels; scale bars 200μm) to demonstrate discrete lymphoid aggregates of T- and B-cells, with scattered adjacent CD138+ cells consistent with the presence of TLS. All four showed similar results to the two tumors depicted in (**c-d**). Holes in the tissue represent areas that were punched for RNA isolation and transcriptomic analyses (**a-d**). Abbreviation: TLS, tertiary lymphoid structure.

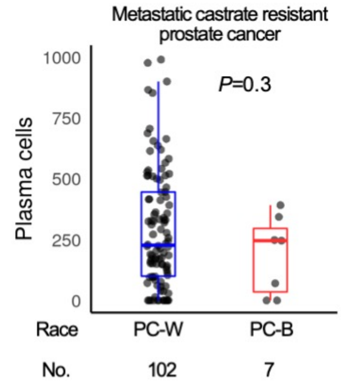

Supplementary figure 4: Plasm cell content by self-identified race in metastatic castrate resistant prostate cancer. An assessment of plasma cell content based on race showed no difference in plasma cell content by race, which may be due to the low numbers of PC-B (two-sided *P*-value based on Wilcoxon Rank Sum). Box plots: center line, median; box limits, upper and lower quartiles; whiskers, 1.5x interquartile range. Abbreviations: PC-B, Prostate cancer from men of self-identified Black race; PC-W, prostate cancer from men of self-identified White race.

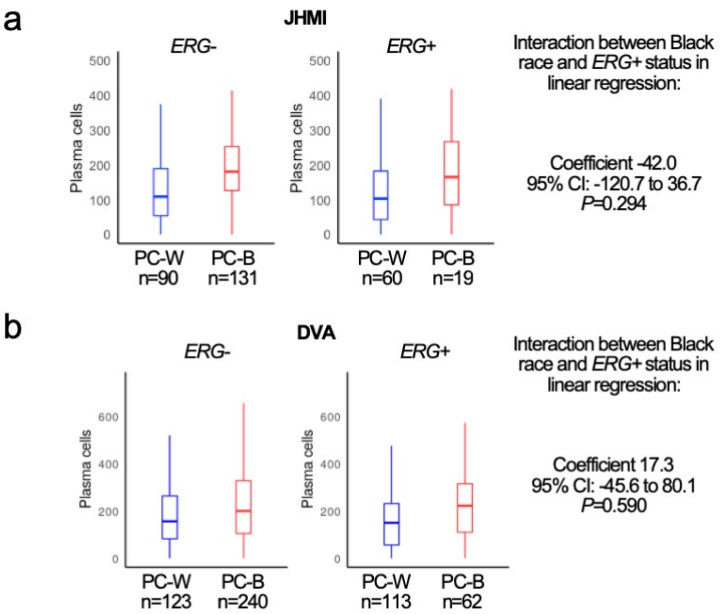

Supplementary figure 5: The association between self-identified Black race and increased plasma cell content is independent of alterations in *ERG*. PC-B is less likely to be defined by *ERG* gene fusion,[5] thus we sought to determine if the association between increased plasma cell content and Black race was related to their propensity to be *ERG*-. In both JHMI (**a**) and DVA (**b**), from linear regressions adjusting for race and *ERG* status based on expression patterns described by Tomlins et al.,[6] the interaction terms between Black race and *ERG*+ status were not statistically significant suggesting Black race is associated with increased plasma cell content independent of *ERG* status. Box plots (**a-b)**: center line, median; box limits, upper and lower quartiles; whiskers, 1.5x interquartile range. All *P*-values were two-side without adjustment for multiple testing. Abbreviations: JHMI, Johns Hopkins Medical Institute; DVA, Durham Veterans Affairs; PC-B, Prostate cancer from men of self-identified Black race; PC-W, prostate cancer from men of self-identified White race; CI, confidence interval.

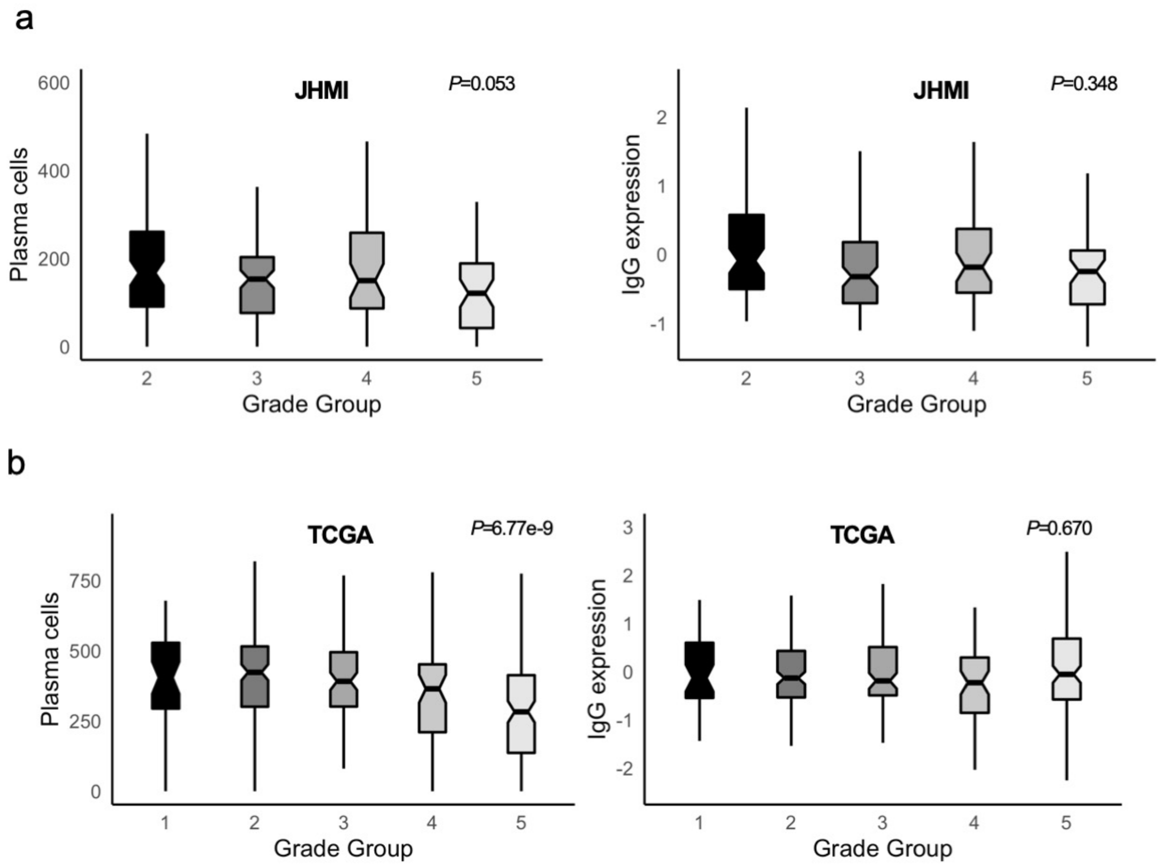

Supplementary figure 6: Tumor plasma cell content and IgG expression stratified by grade group. In JHMI, there was no association between grade group and plasma cell content and IgG expression (**a**; n=300 patients) while in TCGA higher grade tumors tended to have higher plasma cell content (**b**; n=468 patients). Box plots (**a-b**): center line, median; box limits, upper and lower quartiles; whiskers, 1.5x interquartile range. All *P*-values were two-side from Kruskal–Wallis tests without adjustment for multiple testing. Abbreviations: JHMI, Johns Hopkins Medical Institute; TCGA, The Cancer Genome Atlas.

a

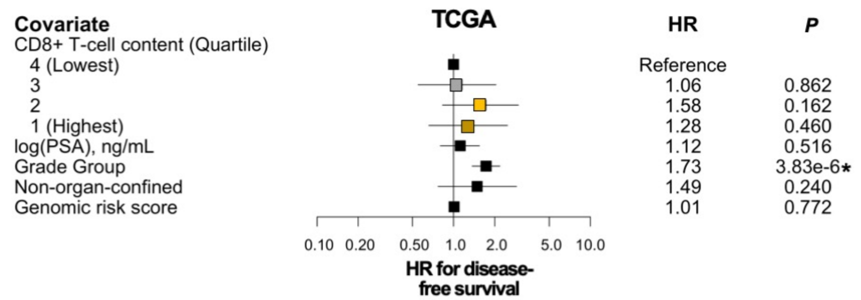

b

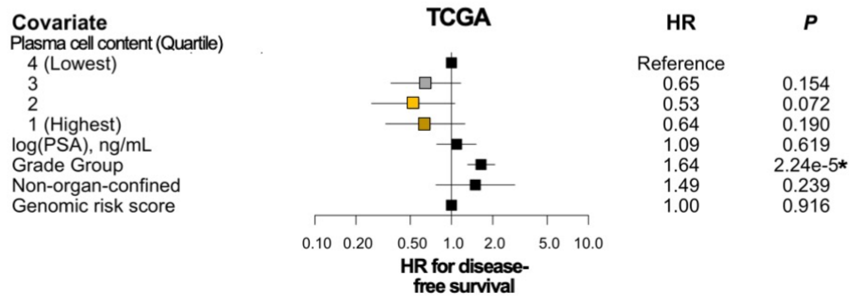

Supplementary figure 7: Multivariable Cox regression for disease-free survival in TCGA. Similar to John Hopkins Medical Institute, in TCGA (n=468 patients) after adjusting for clinical covariates in Cox regressions, higher quantities of plasma cells (**a;** HR ± 95% confidence interval) but not CD8+ T-cells (**b;** HR ± 95% confidence interval) trended towards longer disease-free survival. Asterisk next to *P*-values denote statistical significance with $p<0.05$. All *P*-values were two-side. Abbreviations: TCGA, The Cancer Genome Atlas; HR, hazard ratio; PSA, prostate-specific antigen.

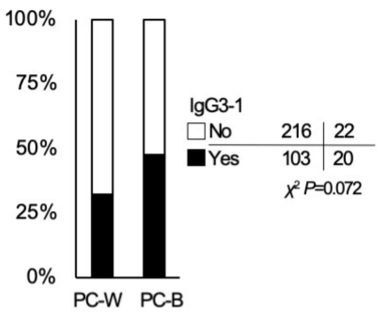

Supplementary figure 8: IgG3 to 1 subclass switch recombination by genetic ancestry in The Cancer Genome Atlas. A higher proportion of PC-B were categorized as having any IgG3 to 1 subclass switch recombination (IgG3-1) based on data from Hu et al.[7] on 361of 468 patients in our cohort from The Cancer Genome Atlas although this was not statistically significant. Two-side *P*-value from χ^2 test. Abbreviations: PC-B, Prostate cancer from men of African genetic ancestry; PC-W, prostate cancer from men of European genetic ancestry.

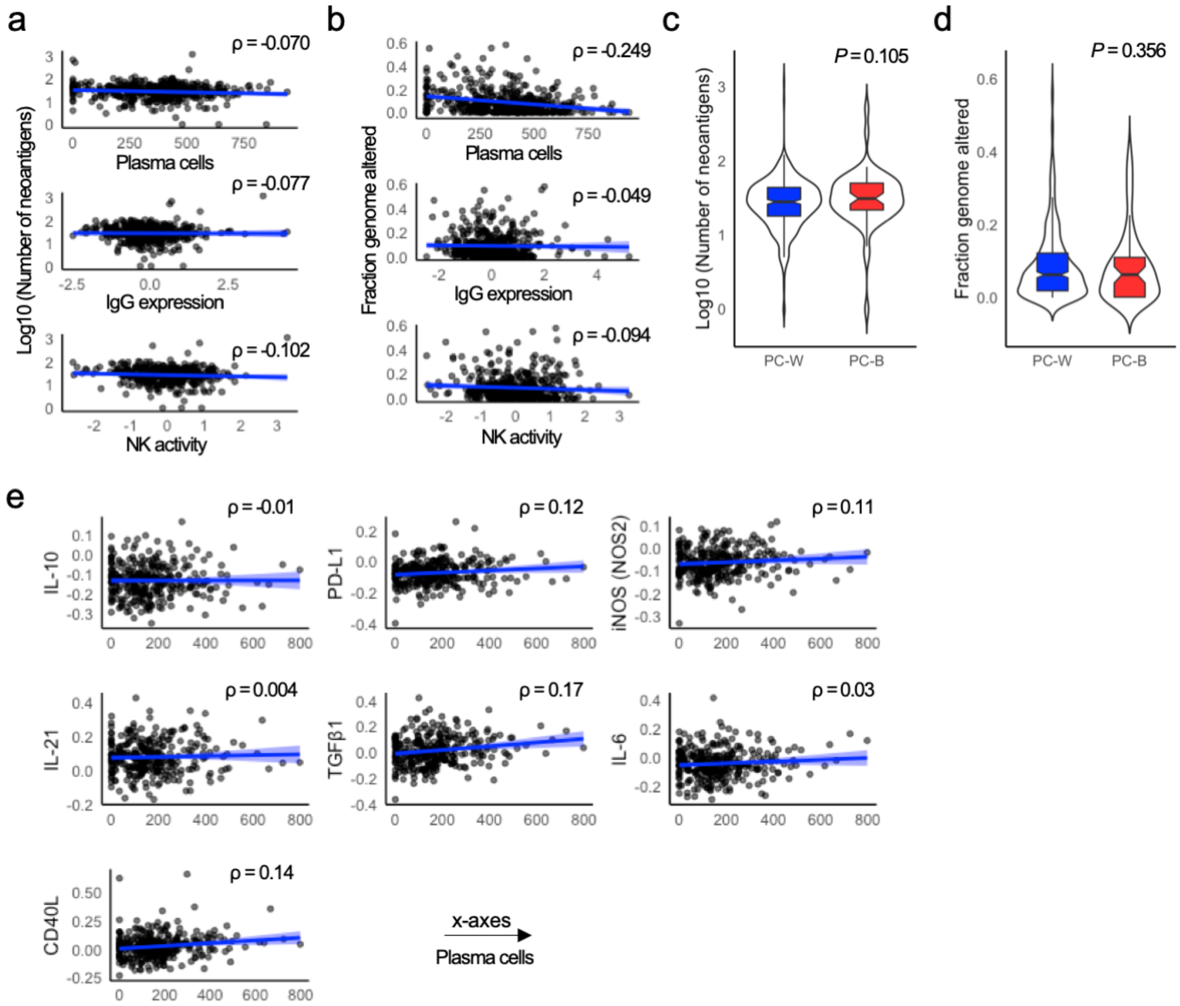

Supplementary figure 9: Signature correlations with mutation burden and inflammatory cytokines. Using data from The Cancer Immune Atlas project (https://tcia.at/home) on the neoantigen burden for 420 of 468 patients in our cohort from TCGA and fraction genome altered as defined as the length of segments with log2 or linear copy number alteration value larger than 0.2 divided by the length of all segments measured as available at https://www.cbioportal.org/study/summary?id=prad_tcga, we show both neoantigen burden and fraction genome altered correlated poorly with plasma cells, IgG, and NK activity (**a-b**; Spearman correlation coefficients; shading represents 95% confidence level interval for predictions from a linear model). Similarly, there was no association between neoantigen burden or fraction genome altered and African genetic ancestry (**c-d**; two-sided *P*-values based on Wilcoxon Rank Sum; center line, median; box limits, upper and lower quartiles; whiskers, 1.5x interquartile range). The expression level of inflammatory cytokines commonly associated with IgA class switch were not correlated with plasma cell content in JHMI (**e**; Spearman correlation coefficients; shading represents 95% confidence level interval for predictions from a linear model; n=300 patients). Abbreviations: JHMI, Johns Hopkins Medical Institute; TCGA, The Cancer Genome atlas; PC-B, Prostate cancer from men of self-identified Black race or African genetic ancestry; PC-W, prostate cancer from men of self-identified White race or European genetic ancestry.

Supplementary tables

Supplementary table 1: Johns Hopkins Medical Institute cohort

Supplementary table 2: The Cancer Genome Atlas cohort

Supplementary table 3: Durham Veterans Affairs cohort

Supplementary table 4: Plasma cell content quartiles by race in Johns Hopkins Medical

Supplementary table 5: Plasma cell content quartiles by race in The Cancer Genome Atlas

Supplementary table 6: Plasma cell content quartiles by race in Durham Veterans Affairs

Supplementary table 7: Metastatic castrate resistant prostate cancer cohort

Supplementary table 8: Interaction analyses

Supplementary table 9: Other subtype tumors from men of African genetic ancestry in The Cancer Genome Atlas with above-median plasma cell content

Supplementary table 10: Summary of immune-based signatures evaluated in prostate tumors by race or genetic ancestry in this study

Supplementary table 1: Johns Hopkins Medical Institute cohort

Characteristic		Self-identified White race n = 150	Self-identified Black race n = 150	*P*
Age				0.015
	Mean (SD)	60.0 (5.7)	58.3 (6.2)	
Grade Group				0.060
	2	49 (32.7%)	42 (28%)	
	3	42 (28%)	60 (40%)	
	4	24 (16%)	27 (18%)	
	5	35 (23.3%)	21 (14%)	
PSA, ng/mL				1.0
	Mean (SD)	9.8 (6.7)	10.8 (8.1)	
	Unknown	0 (0%)	2 (1.3%)	
Organ Confined				0.9
	Yes	110 (73.3%)	110 (73.3%)	
	No	40 (26.7%)	40 (26.7%)	
Margin +				0.145
	No	110 (73.3%)	96 (64%)	
	Yes	40 (26.7%)	52 (34.7%)	
	Unknown	0 (0%)	2 (1.3%)	

χ^2 tests were used to compare categorical variables and student t-tests were used to compare age and PSA. No adjustments for multiple testing were made. All tests were two-sided. Abbreviations: SD, standard deviation; PSA, prostate specific antigen.

Supplementary table 2: The Cancer Genome Atlas cohort

Characteristic		European genetic ancestry n = 410	African genetic ancestry n = 58	*P*
Age				3.71e-5
	Mean (SD)	61.7 (6.5)	57.3 (7.2)	
Grade Group				2.84e-5
	1	30 (7.3%)	12 (20.7%)	
	2	116 (28.3%)	26 (44.8%)	
	3	84 (20.5%)	12 (20.7%)	
	4	57 (13.9%)	2 (3.4%)	
	5	123 (30%)	6 (10.3%)	
PSA, ng/mL				0.779
	Mean (SD)	10.9 (12.4)	11.4 (11.5)	
Organ Confined				0.024
	Yes	149 (36.3%)	31 (53.4%)	
	No	254 (62%)	27 (46.6%)	
	Unknown	7 (1.7%)	0 (0%)	
Margin +				0.7
	No	273 (66.6%)	38 (65.5%)	
	Yes	127 (31%)	15 (25.9%)	
	Unknown	10 (2.4%)	5 (8.6%)	

χ^2 tests were used to compare categorical variables and student t-tests were used to compare age and PSA. No adjustments for multiple testing were made. All tests were two-sided. Abbreviations: SD, standard deviation; PSA, prostate specific antigen.

Supplementary table 3: Durham Veterans Affairs cohort

Characteristic		Self-identified White race n = 236	Self-identified Black race n = 302	*P*
Age				5.74e-8
	Mean (SD)	62.7 (5.7)	59.9 (6.0)	
Grade Group				0.3
	1	35 (14.8%)	30 (9.9%)	
	2	137 (58.1%)	191 (63.2%)	
	3	33 (14%)	48 (15.9%)	
	4	11 (4.7%)	16 (5.3%)	
	5	20 (8.5%)	17 (5.6%)	
PSA, ng/mL				0.021
	Mean (SD)	8.8 (6.7)	10.6 (10.3)	
Organ Confined				0.023
	Yes	119 (50.4%)	183 (60.6%)	
	No	117 (49.6%)	119 (39.4%)	
Margin +				0.145
	No	31 (13.1%)	45 (14.9%)	
	Yes	205 (86.9%)	257 (85.1%)	
Adjuvant hormonal therapy				0.6
	No	177 (75%)	219 (72.5%)	
	Yes	59 (25%)	82 (27.2%)	
	Unknown	0 (0%)	1 (0.3%)	
Adjuvant radiotherapy				0.142
	No	132 (55.9%)	148 (49%)	
	Yes	104 (44.1%)	153 (50.7%)	
	Unknown	0 (0%)	1 (0.3%)	

χ^2 tests were used to compare categorical variables and student t-tests were used to compare age and PSA. No adjustments for multiple testing were made. All tests were two-sided. Abbreviations: SD, standard deviation; PSA, prostate specific antigen.

Supplementary table 4: Plasma cell content quartiles by race in Johns Hopkins Medical Institute

Plasma cell content (Quartile)	PC-W, n=150	PC-B, n=150
4 (Lowest)	52 (34.7%)	23 (15.3%)
3	42 (28.0%)	33 (22.0%)
2	32 (21.3%)	43 (28.7%)
1 (Highest)	24 (16.0%)	51 (34.0%)

Two-sided Cochrane-Armitage test for trend, P = 1.21e-06. Abbreviations: PC-B, Prostate cancer from men of self-identified Black race; PC-W, prostate cancer from men of self-identified White race

Supplementary table 5: Plasma cell content quartiles by race in The Cancer Genome Atlas

Plasma cell content (Quartile)	PC-W, n=410	PC-B, n=58
4 (Lowest)	105 (25.6%)	12 (20.7%)
3	112 (27.3%)	5 (8.6%)
2	105 (25.6%)	12 (20.7%)
1 (Highest)	88 (21.5%)	29 (50.0%)

Two-sided Cochrane-Armitage test for trend, P = 2.74e-4. Abbreviations: PC-B, Prostate cancer from men of African genetic ancestry; PC-W, prostate cancer from men of European genetic ancestry.

Supplementary table 6: Plasma cell content quartiles by race in Durham Veterans Affairs

Plasma cell content (Quartile)	PC-W, n=236	PC-B, n=302
4 (Lowest)	75 (31.2 %)	60 (19.9%)
3	62 (26.3%)	72 (23.8%)
2	57 (24.2%)	77 (25.5%)
1 (Highest)	42 (17.8%)	93 (30.8%)

Two-sided Cochrane-Armitage test for trend, *P* = 5.46e-5. Abbreviations: PC-B, Prostate cancer from men of self-identified Black race; PC-W, prostate cancer from men of self-identified White race

Supplementary table 7: Metastatic castrate resistant prostate cancer cohort

Characteristic		n (%)
All		118 (100)
Age		
	Mean (Standard deviation)	67.2 (8.3)
Self-identified race		
	White	102 (86.4)
	Black	7 (5.9)
	Unknown/Other	9 (7.6)
Biopsy site		
	Visceral	22 (18.6)
	Lymph node	50 (42.4)
	Skeletal	31 (26.3)
	Unknown/Other	15 (12.7)
Prior taxane treatment		
	Yes	44 (37.3)
	No	71 (60.2)
	Unknown	3 (2.5)
Prior abiraterone or enzalutamide treatment		
	Yes	54 (45.8)
	No	61 (51.7)
	Unknown	3 (2.5)

Supplementary table 8: Interaction analyses

Cohort	Variable		Estimate of interaction with self-identified Black race or African genetic ancestry (95% CI)	*P*
JHMI, n=268	PTEN loss (Immunohistochemistry)		-41.67 (117.10 to 33.76)	0.3
TCGA, n=468	PTEN loss (Homozygous deletion)		-193.94 (-408.97 to 21.08)	0.077
DVA, n=467	PTEN loss (RNA-based signature)		19.22 (-137.37 to 175.81)	0.8
JHMI, n=298	PSA (Per increase by 10 ng/mL)		4.26 (-37.73 to 46.25)	0.8
DVA, n=522	PSA (Per increase by 10 ng/mL)		11.51 (-26.0 to 49.0)	0.5
JHMI, n=300	Age quartile			
		1 (Youngest)	Reference	
		2	-59.53 (-149.24 to 30.18)	0.193
		3	-2.73 (-85.78 to 80.32)	0.9
		4 (Oldest)	-61.99 (-145.11 to 21.14)	0.143
DVA, n=538	Age quartile			
		1 (Youngest)	Reference	
		2	13.87 (-69.64 to 97.38)	0.7
		3	-3.62 (-84.28 to 77.05)	0.9
		4 (Oldest)	-89.30 (-165.07 to 4.48)	0.063
TCGA, n=320	Subtype			
	ERG		Reference	
	ETV1		50.18 (-224.72 to 325.09)	0.7
	ETV4		-38.54 (-413.6 to 336.52)	0.8
	FLI1		129 (-284.06 to 542.07)	0.5
	SPOP		-46.62 (-212.9 to 119.67)	0.6
	FOXA1		-278.9 (-660.55 to 102.76)	0.151
	IDH1		-371.81 (-808.37 to 64.76)	0.095
	**Other**		**159.58 (17.62 to 301.55)**	**0.028**

Multiple multivariable linear regressions were performed for the outcome of plasma cell content. Included in each regression was race (Self-identified White vs Black in JHMI and DVA) or genetic ancestry (African vs European in TCGA), the variable listed in this table, and an interaction between the respective variable and Black race or African ancestry. Patients were excluded from these analyses for missing data or if PTEN loss based on RNA-based signature was “Indeterminate” in the DVA cohort. Bolding indicates statistical significance at p<0.05. All *P*-values are two-sided. No adjustments were made for multiple testing. Abbreviations: JHMI, Johns Hopkins Medical Institute; TCGA, The Cancer Genome atlas; DVA, Durham Veterans Affairs; CI, confidence interval.

Supplementary table 9: Other subtype tumors from men of African genetic ancestry in The Cancer Genome Atlas with above-median plasma cell content

TCGA-EJ-8470
TCGA-G9-6378
TCGA-HC-7737
TCGA-HC-A4ZV
TCGA-KC-A4BL
TCGA-KK-A6E5
TCGA-KK-A7AV
TCGA-KK-A8IL
TCGA-V1-A8MF
TCGA-V1-A8MG
TCGA-V1-A8ML
TCGA-V1-A8MU
TCGA-V1-A8WL
TCGA-VP-A87E

Supplementary table 10: Summary of immune-based signatures evaluated in prostate tumors by race or genetic ancestry in this study

**Signature/measure**	**Reference or source**	**Evaluated based on self-identified race, genetic ancestry, or both**	**Note**
Lymphocyte evasion score	Jiang *et al.*[8]	Self-identified race	Lower in Black men
Immune content	Yoshihara *et al.*[9]	Both	Higher in Black men and men of African ancestry
Plasma cell content	Chen *et al.*[2]	Both	Higher in Black men and men of African ancestry
Inflammation	Liberzon *et al.*[10]	Both	More in Black men
Interferon gamma	Liberzon *et al.*[10]	Both	More in Black men
B-lineage cells	Becht *et al.*[1]	Both	More in Black men and men of African ancestry
CD-19+ cells	Chakravarthy *et al.*[3]	Both	More in Black men and men of African ancestry
CD79a+ cells	IHC	Self-identified race	More in Black men
CD138a+ cells	IHC	Self-identified race	More in Black men
IgG3 to 1 class switch recombination	Hu *et al.*[7]	Genetic ancestry	Trend towards more in men of African ancestry
Neoantigen burden	https://tcia.at/[11]	Genetic ancestry	No difference based on genetic ancestry
Fraction genome altered	cBioPortal[12,13]	Genetic ancestry	No difference based on genetic ancestry

Abbreviation: IHC, immunohistochemistry

Supplementary methods

Johns Hopkins Medical Institute

The main strengths of JHMI are related to clinical analyses which made this cohort our *a piori* main source for outcome analyses. This cohort was built excluding men with low grade (grade group 1) PC for whom the preferred treatment would be active surveillance as oppose to radical prostatectomy due to their low risk of developing metastatic disease and dying from PC and the disproportionate numbers of grade group 1 based on self-identified race (6 prostate cancers from men of self-identified White race or European genetic ancestry [PC-W] and 24 prostate cancer from men of self-identified Black race or African genetic ancestry [PC-B]).[16–18] All patients were uniform in that none received adjuvant treatment prior to any disease recurrence. Since all patients where managed at a single institution, the follow-up regimens for disease monitoring were likely similar. Finally, data on metastatic recurrence was available as opposed to biochemical recurrence which is less predictive of death from prostate cancer.[19,20] Additionally, the cohort had a high number of PC-B that was matched for grade and stage to PC-W which likely reduced some of the bias related to clinical variables on the primary findings in the study. Race in JHMI was patient-identified which allows for assessment based on social construct and likely captures the exposures relevant to African-Americans.

Limitations include the tissue procurement and handling. The source tissues for JHMI were derived from formalin-fixed paraffin embedded (FFPE) specimens which were several years old prior to RNA extraction. As noted in previous works, the absolute value of gene expression measurements may be decreased in FFPE specimens compared to that of fresh-frozen (FF) tissue yet are still considered reliable and show similar relative differences to cohorts with higher quality gene expression data.[21] This accounts for the relative discrepancies in the heights of the bars in **Figure 1c** which is an expression-based measurement of tumor immune content. Other limitations include lack of genetic data within JHMI.

The Cancer Genome Atlas

The strengths of TCGA include the availability of genetic ancestry. As noted in previous work, self-define African Americans may be substantially heterogeneous in terms of genomic ancestry.[22] Thus, the use of genetic ancestry as previously determined[23] in this cohort serves as an additional source of validation towards race/ancestry-based findings in the current work. The availability of methylation and genetic data in TCGA also allow for assessments of immune cell signatures in addition to expression-based measures and also afforded analyses of effect modification of genetic alterations on the primary findings in the study. Finally, TCGA RNA extraction occurred from FF tissue. Thus, the RNA expression levels are the least attenuated of the three cohorts.

Limitations of TCGA include the clinical follow-up. In TCGA, disease-free survival (DFS) is defined as the earlier of development of metastatic disease or biochemical recurrence. Since death and prostate-cancer-specific death was so uncommon on TCGA,[24] the best outcomes to use for failure are metastatic recurrence and biochemical recurrence with the former being a much more prognostic surrogate endpoint for death.[19] Additionally, TCGA is comprised of patients from multiple institutions with likely heterogeneous follow-up regimens which would likely contribute to bias in terms of recurrence detection depending on the frequency of disease monitoring. Patients in TCGA may have also received adjuvant treatment prior to a disease recurrence which ultimately may have impacted the timing of recurrence. Similarly, unlike JHMI, this cohort was not matched on clinical covariates based on genetic ancestry. Finally, TCGA has a limited number of PC-B and was not a matched cohort like JHMI which reduces the overall power to validate findings.

Durham Veterans Affairs

The major strength of DVA is its large number of tumors with self-identified race which allow for validation of major expression-based findings. This cohort represents one of the largest collection of PC-B with RNA-expression data.

Limitations include data on clinical outcomes in DVA. While data on recurrence has been reported for this cohort,[25] the DVA cohort has several qualities that render it a more limited resource for time-to-event analyses in this study. About 25% of men received hormonal therapy following surgery and nearly half received radiotherapy (**Supplementary table 3**). These additional treatments and their timing following surgery would surely influence any clinical outcomes in substantial ways that would preclude meaningful analyses. For instance, 60% of patients with tumors in the lowest quartile of plasma cell content received radiotherapy and 35% received hormonal therapy. These values were 45% and 25%, respectively, in patients with tumors in the highest quartile of plasma cell content. Additionally, this cohort is noticeably lower-risk relative to the JMHI. In JHMI 36% (107/300) of the cohort had high grade (grade group 4 or 5) disease). In DVA only 12% (64/538) were high grade and thus the general risk of developing metastatic disease and death from prostate was very low. Accordingly, only 5.4% (29/538) developed metastatic disease. This value is low relative to metastatic recurrence in JHMI (24%) and disease recurrence in TCGA (17.5%). Additionally, about 25% of men received hormonal therapy following surgery and nearly half received radiotherapy (**Supplementary table 3**). These additional treatments, their timing following surgery, and duration and dosing would influence clinical outcomes assessments in indeterminable ways. For instance, 60% of patients with tumors in the lowest quartile of plasma cell content received radiotherapy and 35% received hormonal therapy. These values were 45% and 25%, respectively, in patients with tumors in the highest quartile of plasma cell content. For these reasons, we did not include survival analyses for DVA.

Also, unlike JHMI, this cohort was not matched on clinical covariates based on race. Notably, however, tumors grade group did not differ by race and although serum PSA and organ confined disease did differ by race based on statistical significance, these differences were not substantial. Finally, similar to JHMI, the source tissues for DVA were derived from FFPE specimens with RNA extraction performed several years following fixation. As noted above, this would account for lower absolute value gene expression but not relative gene expression within each tumor.

Supplementary references

1. Becht, E. *et al.* Estimating the population abundance of tissue-infiltrating immune and stromal cell populations using gene expression. *Genome Biology* **17**, 218 (2016).
2. Chen, S.-H. *et al.* A gene profiling deconvolution approach to estimating immune cell composition from complex tissues. *BMC Bioinformatics* **19**, 154 (2018).
3. Chakravarthy, A. *et al.* Pan-cancer deconvolution of tumour composition using DNA methylation. *Nature Communications* **9**, 1–13 (2018).
4. Kaur, H. B. *et al.* Association of Tumor Infiltrating T-cell Density with Molecular Subtype, Racial Ancestry and Clinical Outcomes in Prostate Cancer. *Mod Pathol* **31**, 1539–1552 (2018).
5. Tosoian, J. J. *et al.* Prevalence and Prognostic Significance of PTEN Loss in African-American and European-American Men Undergoing Radical Prostatectomy. *European Urology* **71**, 697–700 (2017).
6. Tomlins, S. A. *et al.* Characterization of 1577 Primary Prostate Cancers Reveals Novel Biological and Clinicopathologic Insights into Molecular Subtypes. *European Urology* **68**, 555–567 (2015).
7. Hu, X. *et al.* Landscape of B cell immunity and related immune evasion in human cancers. *Nature Genetics* **51**, 560–567 (2019).
8. Jiang, P. *et al.* Signatures of T cell dysfunction and exclusion predict cancer immunotherapy response. *Nat Med* **24**, 1550–1558 (2018).
9. Yoshihara, K. *et al.* Inferring tumour purity and stromal and immune cell admixture from expression data. *Nature Communications* **4**, 1–11 (2013).
10. Liberzon, A. *et al.* The Molecular Signatures Database Hallmark Gene Set Collection. *Cell Syst.* **1**, 417–425 (2015).
11. Szolek, A. *et al.* OptiType: precision HLA typing from next-generation sequencing data. *Bioinformatics* **30**, 3310–3316 (2014).

12. Cerami, E. *et al.* The cBio Cancer Genomics Portal: An Open Platform for Exploring Multidimensional Cancer Genomics Data. *Cancer Discov* **2**, 401–404 (2012).

13. Gao, J. *et al.* Integrative Analysis of Complex Cancer Genomics and Clinical Profiles Using the cBioPortal. *Sci. Signal.* **6**, pl1–pl1 (2013).

14. Cabrita, R. *et al.* Tertiary lymphoid structures improve immunotherapy and survival in melanoma. *Nature* **577**, 561–565 (2020).

15. Cursons, J. *et al.* A Gene Signature Predicting Natural Killer Cell Infiltration and Improved Survival in Melanoma Patients. *Cancer Immunol Res* **7**, 1162–1174 (2019).

16. Klotz, L. *et al.* Long-Term Follow-Up of a Large Active Surveillance Cohort of Patients With Prostate Cancer. *JCO* **33**, 272–277 (2014).

17. Tosoian, J. J. *et al.* Intermediate and Longer-Term Outcomes From a Prospective Active-Surveillance Program for Favorable-Risk Prostate Cancer. *JCO* **33**, 3379–3385 (2015).

18. Mohler, J. L. *et al.* Prostate Cancer, Version 2.2019, NCCN Clinical Practice Guidelines in Oncology. *J Natl Compr Canc Netw* **17**, 479–505 (2019).

19. Xie, W. *et al.* Metastasis-Free Survival Is a Strong Surrogate of Overall Survival in Localized Prostate Cancer. *JCO* **35**, 3097–3104 (2017).

20. Xie, W. *et al.* Event-Free Survival, a Prostate-Specific Antigen–Based Composite End Point, Is Not a Surrogate for Overall Survival in Men With Localized Prostate Cancer Treated With Radiation. *JCO* JCO.19.03114 (2020) doi:10.1200/JCO.19.03114.

21. Hedegaard, J. *et al.* Next-Generation Sequencing of RNA and DNA Isolated from Paired Fresh-Frozen and Formalin-Fixed Paraffin-Embedded Samples of Human Cancer and Normal Tissue. *PLOS ONE* **9**, e98187 (2014).

22. Bryc, K. *et al.* Genome-wide patterns of population structure and admixture in West Africans and African Americans. *PNAS* **107**, 786–791 (2010).

23. Yuan, J. *et al.* Integrated Analysis of Genetic Ancestry and Genomic Alterations across Cancers. *Cancer Cell* **34**, 549-560.e9 (2018).

24. Liu, J. *et al.* An Integrated TCGA Pan-Cancer Clinical Data Resource to Drive High-Quality Survival Outcome Analytics. *Cell* **173**, 400-416.e11 (2018).

25. Howard, L. E. *et al.* Validation of a genomic classifier for prediction of metastasis and prostate cancer-specific mortality in African-American men following radical prostatectomy in an equal access healthcare setting. *Prostate Cancer and Prostatic Diseases* 1–10 (2019) doi:10.1038/s41391-019-0197-3.
