# Supplementary material for: Plasma cells are enriched in localized prostate cancer in Black men and are associated with improved outcomes
Source: Nat Commun. 2021 Feb 10;12:935. doi: 10.1038/s41467-021-21245-w (PMC7876147; doi:10.1038/s41467-021-21245-w)
Supplement: Supplementary file 3 — Description of Additional Supplementary Files [file 41467_2021_21245_MOESM3_ESM.pdf]

Description of additional supplementary files

File name: Supplementary data 1
Frequency of copy number alterations in the 14 tumors from Black men in The Cancer Genome Atlas who were categorized as "Other" non-subtypeable prostate cancer with above-median plasma cell content

File name: Supplementary data 2
Frequency of gene mutations in the 14 tumors from Black men in TCGA who were categorized as "Other" non-subtypeable prostate cancer with above-median plasma cell content
