# Supplementary material for: Plasma cells are enriched in localized prostate cancer in Black men and are associated with improved outcomes
Source: Nat Commun. 2021 Feb 10;12:935. doi: 10.1038/s41467-021-21245-w (PMC7876147; doi:10.1038/s41467-021-21245-w)
Supplement: Supplementary file 6 — Reporting Summary [file 41467_2021_21245_MOESM6_ESM.pdf]

nature research

Corresponding author(s): Edward M. Schaeffer, MD, PhD

Last updated by author(s): Nov 22, 2020

Reporting Summary

Nature Research wishes to improve the reproducibility of the work that we publish. This form provides structure for consistency and transparency in reporting. For further information on Nature Research policies, see our Editorial Policies and the Editorial Policy Checklist.

Statistics

For all statistical analyses, confirm that the following items are present in the figure legend, table legend, main text, or Methods section.

n/a	Confirmed	
☐	☒	The exact sample size (n) for each experimental group/condition, given as a discrete number and unit of measurement
☐	☒	A statement on whether measurements were taken from distinct samples or whether the same sample was measured repeatedly
☐	☒	The statistical test(s) used AND whether they are one- or two-sided<br>*Only common tests should be described solely by name; describe more complex techniques in the Methods section.*
☐	☒	A description of all covariates tested
☐	☒	A description of any assumptions or corrections, such as tests of normality and adjustment for multiple comparisons
☐	☒	A full description of the statistical parameters including central tendency (e.g. means) or other basic estimates (e.g. regression coefficient) AND variation (e.g. standard deviation) or associated estimates of uncertainty (e.g. confidence intervals)
☐	☒	For null hypothesis testing, the test statistic (e.g. F, t, r) with confidence intervals, effect sizes, degrees of freedom and P value noted<br>*Give P values as exact values whenever suitable.*
☒	☐	For Bayesian analysis, information on the choice of priors and Markov chain Monte Carlo settings
☒	☐	For hierarchical and complex designs, identification of the appropriate level for tests and full reporting of outcomes
☒	☐	Estimates of effect sizes (e.g. Cohen's d, Pearson's r), indicating how they were calculated

Our web collection on statistics for biologists contains articles on many of the points above.

Software and code

Policy information about availability of computer code

Data collection	QuPath version 0.1.2 was used to quantify CD79a and CD138 density (https://www.nature.com/articles/s41598-017-17204-5)
Data analysis	RStudio version 1.2.5019 (Boston, MA)<br>methyCIBERSORT version 0.2.1<br>minfi version 1.36.0<br>MCP-counter version 1.1.0<br>MySort (default version)<br>ESTIMATE version 2.0.0<br>TIDE: http://tide.dfci.harvard.edu/ (default version)<br>OptiType version 1.2<br>Single Channel Array Normalization (SCAN) algorithm SCAN.UPC" version 2.32.0 R package<br>Matchit version 3.0.2

For manuscripts utilizing custom algorithms or software that are central to the research but not yet described in published literature, software must be made available to editors and reviewers. We strongly encourage code deposition in a community repository (e.g. GitHub). See the Nature Research guidelines for submitting code & software for further information.

Data

Policy information about availability of data

All manuscripts must include a data availability statement. This statement should provide the following information, where applicable:

- Accession codes, unique identifiers, or web links for publicly available datasets
- A list of figures that have associated raw data
- A description of any restrictions on data availability

The data that support the findings of this study from the Johns Hopkins Medical Institute and Durham Veterans Affairs Hospital were deposited on NCBI Gene Expression Omnibus (GEO) and are accessible through GEO Series accession number GSE153352 [https://www.ncbi.nlm.nih.gov/geo/query/acc.cgi?acc=GSE153352] and GSE157547 [https://www.ncbi.nlm.nih.gov/geo/query/acc.cgi?acc=GSE157547], respectively. The source data underlying Figures 1a-g, 2a-d, 3a-c, 4a-g, Supplementary figures 1a-c, 1f-h, 2a-b, 4, 5a-b, 6a-b, 7a-b, 8, and 9a-e and Supplementary table 9 are provided as a Source Data file. The remaining data are available within the Article, Supplementary Information or available from the authors upon request.

Field-specific reporting

Please select the one below that is the best fit for your research. If you are not sure, read the appropriate sections before making your selection.

☒ Life sciences ☐ Behavioural & social sciences ☐ Ecological, evolutionary & environmental sciences

For a reference copy of the document with all sections, see nature.com/documents/nr-reporting-summary-flat.pdf

Life sciences study design

All studies must disclose on these points even when the disclosure is negative.

Sample size	The JHMI cohort was derived from two groups of patients with available clinical and expression data who underwent radical prostatectomy at JHMI without any adjuvant treatment until the end of follow-up or metastatic recurrence. After exclusions (see below), there were 150 Black men and 302 White men available for matching. We matched the cohort 1:1 by race to use all the included Black men (150 Black men and 150 White men). Thus, this cohort consisted of 300 men total with expression data from 1 sample from each patient.<br><br>From TCGA, we based our sample size on all the available patients in TCGA with generated genetic ancestry as White (European American) or Black (African American) from the Cancer Genetic Ancestry Atlas (TCGAA, http://52.25.87.215/TCGAA). Thus, this cohort consisted of 410 White and 58 Black men with expression data from 1 sample from each patient.<br><br>From the DVA cohort, all men were included resulting in 236 White and 302 Black men with expression data from 1 sample from each patient.<br><br>No formal sample size calculation was conducted. However, the sample sizes were deemed sufficient given DVA represented the largest cohort of tumors with whole transcriptome data from localized prostate cancer from men annotated as Black race. Similarly, JHMI represented the largest cohort with expression data from tumors from Black men and detailed clinical outcomes. Additionally, given previous work from TCGA (https://doi.org/10.1371/journal.pgen.1008641) had noted differences in immune expression signaling between tumors from White and Black men with the limited sample of tumors from Black men in TCGA (the same cohort we used), our sample sizes from JHMI and DVA were, again, deemed sufficient for discovery of differential expression of clinically relevant immune signatures.
Data exclusions	From a total of 312 White men and 176 Black men in the original JHMI cohort, 6 (1%) men were excluded due to missing data on pathological stage (4 White men and 2 Black men). All men with grade group 1 disease were excluded given the disproportionate numbers among Black men (24 vs 6; 30 total, 6%). Additionally, excluding men with grade group 1 prostate cancer would create a cohort more ideal for our analyses of outcomes following radical prostatectomy since the preferred treatment for most men with low grade PC is active surveillance with deferred treatment for signs of disease progression. From this, we matched all 150 Black men to 150 White (see below) to create our final cohort thus excluding another 152 white men from the final cohort of 300.
Replication	To quantify CD79a+ and CD138 cell density, the number of cells within each 0.6 mm diameter tissue microarray punch of tumor (average of four spots sampled per case), normalized by the total mm^2 of tissue analyzed and taking the mean across the four tumor cores (biological replicates) for each patient. All these biological replicates were successful. Additionally, in Supplementary figure 3, three randomly selected tumors that were in the lowest pentile of the TLS signature were stained for CD3+ and CD20+ and each were successful and showed similar results. Four tumors were randomly chosen that were in the highest pentile of the TLS signature and were stained for CD3+, CD20+, and CD138+ cells and each showed similar results and were successful.
Randomization	In the JHMI cohort, the cohort sorted using a random list of numbers generated by the “sample” function from the “base” R package before using the “matchit” function from the “Matchit” R package to derive a final grade- and stage-matched cohort of 300 men, 150 each of Black and White men. Additionally, 2 validation cohorts and multiple orthogonal methods were used to confirm findings noted in the discovery cohort (JHMI). Multiple analyses were conducted to assess for effect modifiers (supplementary table 8 and supplementary figures 5a-b). In the regression assessing the association between race and immune content (Figure 1b), stromal content was included as a regressor. In all multivariable cox regressions (Figures 3b-c, 4c, and 4g and Supplementary figures 7a-b), regressors included well validated risk-factors for adverse outcomes for localized prostate cancer.

To create the figures in Supplementary figure 3, as above, three tumors with low TLS scores and 4 tumors with high TLS scores were randomly chosen to confirm the absence and presence, respectively, of TLS on immunohistochemistry.

Blinding	The pathologists who manually quantified the CD138+ cell density were blinded to patient and clinical characteristics of the tumors.<br><br>The pathologists who determined the PTEN status of tumors based on immunohistochemistry were blinded to patient and clinical characteristics of the tumors.

Reporting for specific materials, systems and methods

We require information from authors about some types of materials, experimental systems and methods used in many studies. Here, indicate whether each material, system or method listed is relevant to your study. If you are not sure if a list item applies to your research, read the appropriate section before selecting a response.

Materials & experimental systems

n/a	Involved in the study
☐	☒ Antibodies
☒	☐ Eukaryotic cell lines
☒	☐ Palaeontology and archaeology
☒	☐ Animals and other organisms
☐	☒ Human research participants
☐	☒ Clinical data
☒	☐ Dual use research of concern

Methods

n/a	Involved in the study
☒	☐ ChIP-seq
☒	☐ Flow cytometry
☒	☐ MRI-based neuroimaging

Antibodies

Antibodies used

We used only commercially available antibodies. All dilutions are in the methods section of the text.

CD79a (JCB117; CellMarque) Mouse Monoclonal Antibody
CD138/syndecan-1 (B-A38; Ventana/Roche) Mouse Monoclonal Antibody
CD3 (A0452; Dako) Rabbit Polyclonal Antibody
CD20 (L26; Ventana/Roche) Mouse Monoclonal Antibody
PTEN (Clone D4.3 XP, Cell Signaling Technology®) rabbit antihuman

Validation

CD79a (JCB117; CellMarque) Mouse Monoclonal Antibody

CD79 is a dimeric transmembrane protein with two distinct component chains: CD79a and CD79b.1-3 CD79a is expressed in B cells from the pro-B stage through B-cell differentiation.2 CD79a is a useful marker for B-cell neoplasms.1-3

1. Mason DY, et al. CD79a: a novel marker for B-cell neoplasms in routinely processed tissue samples. Blood. 1995; 86:1453-9.
2. Bhargava P, et al. CD79a is heterogeneously expressed in neoplastic and normal myeloid precursors and megakaryocytes in an antibody clone-dependent manner. Am J Clin Pathol. 2007; 128:306-13.
3. Chu PG, et al. CD79: a review. Appl Immunohistochem Mol Morphol. 2001; 9:97-106.

CD138/syndecan-1 (B-A38; Ventana/Roche) Mouse Monoclonal Antibody

CD138, Syndecan 1, is expressed in the late stages of B-cell differentiation with progression towards plasma cells.1,2 It can be used to differentiate lymphoplasmacytic lymphoma from marginal zone lymphoma.1 ALK+ large B-cell lyphoma (LBCL) usually strongly expresses CD138 whereas lineage-associated markers such as anti-CD20 and anti-CD79a do not stain ALK+LBCL.3,7 Anti-CD138 is immunoreactive with HHV8-associated primary effusion lymphoma even though the lymphoma cells lack the expression of B-cell markers.5 Anti-CD138 is a good marker to identify and enumerate plasma cells, benign, reactive, or malignant, in bone marrow biopsy specimens.4,6 CD138 is also expressed in epithelial cells.6

1. Chilosi M, et al. Mod Pathol. 1999; 12:1101-6.
2. Sebestyén A, et al. Br J Haematol. 1999; 104:412-9.
3. Delsol G, et al. WHO Press, Geneva, Switzerland. 254.
4. Bayer-Garner IB, et al. Mod Pathol. 2001; 14:1052-8.
5. Said J, et al. WHO Press, Geneva, Switzerland. 261.
6. O'Connell FP, et al. Am J Clin Pathol. 2004; 121:254-63.
7. Colomo L, et al. Am J Surg Pathol. 2004; 28:736-47.

CD3 (A0452; Dako)) Rabbit Polyclonal Antibody

Intended use

For in vitro diagnostic use. Monoclonal Mouse Anti-Human CD3, Clone F7.2.38, is intended for use in immunohistochemistry (IHC). The antibody labels CD3 and is a useful aid for the identification of T cells. Results aid in the classification of T-cell neoplasms (1). Differential classification is aided by the results from a panel of antibodies. The clinical interpretation of any staining or its absence should be complemented by morphological studies using proper controls and should be evaluated within the context of the patient's clinical history and other diagnostic tests by a qualified pathologist. This antibody is intended to be used after the primary diagnosis of tumor has been made by conventional histopathology using nonimmunologic histochemical stains.

Synonyms for antigen

T3, CD3 complex (2).

Summary and explanation

The CD3 complex is composed of six polypeptides with usually four different transmembrane CD3 chains, gamma delta epsilon and zeta Three different dimers, gamma- epsilon, delta-epsilon, and zeta-zeta constitute the CD3 complex. The Mr of CD3-epsilon is 20 000 (2) CD3 is first detectable in early thymocytes and its appearance probably represents one of the earliest signs of commitment to the T-cell lineage (3). In cortical thymocytes, the antigen is predominantly present as an intracytoplasmic constituent. It appears subsequently, at the medullar thymocyte stage, on the T-cell surface in close association with the T-cell receptor (TCR). Whereas the TCR forms the ligand-binding part of the TCR/CD3 complex, the function of the CD3 molecule is that of signalling, making CD3 a highly specific marker for T cells. No other cells are known to express the CD3 molecule, although two monoclonal antibodies raised against CD3 have been found to react with Purkinje cells in the cerebellum (4). The CD3 molecule may be present in the great majority of T-cell neoplasms, although occasional tumors are encountered in which the antigen is lost as part of the neoplastic process (5). The CD3 molecule may also be expressed in some cases of malignant histiocytosis (6) and Hodgkin's lymphoma (7). CD3- epsilon and not the whole CD3 molecule, has been detected in the cytoplasm of natural killer (NK) cells (8), and CD3- epsilon may be a marker of nasal T-cell lymphomas which are thought to be of NK cell origin (9).

CD20 (L26; Ventana/Roche) Mouse Monoclonal Antibody

CD20 is a transmembrane protein in late B-cell precursors and mature B-cells that plays a role in regulating proliferation and differentiation. CD20 expression is lost at the plasma cell stage of differentiation.1 Anti-CD20 (pan B-cell)2 has rarely been detected in T-cell malignancies, and is a dependable marker of B-cell lymphomas3 such as DLBCL.4 CD20 expression is present in some thymomas.

1. Tedder T, et al. CD20: a regulator of cell-cycle progression of B lymphocytes. Immunol Today. 1994; 15:450-4.
2. Mason DY, et al. Antibody L26 recognizes an intracellular epitope on the B-cell-associated CD20 antigen. Am J Pathol. 1990; 136:1215-22.
3. Norton AJ, et al. Monoclonal antibody L26: an antibody that is reactive with normal and neoplastic B lymphocytes in routinely fixed and paraffin wax embedded tissues. J Clin Pathol. 1987; 40:1405-12.
4. Suzuki Y, et al. Association of CD20 levels with clinicopathological parameters and its prognostic significance for patients with DLBCL. Ann Hematol. 2012; 91:997-1005.

PTEN (Clone D4.3 XP, Cell Signaling Technology®) rabbit antihuman

PTEN (phosphatase and tensin homologue deleted on chromosome ten), also referred to as MMAC (mutated in multiple advanced cancers) phosphatase, is a tumor suppressor implicated in a wide variety of human cancers (1). PTEN encodes a 403 amino acid polypeptide originally described as a dual-specificity protein phosphatase (2). The main substrates of PTEN are inositol phospholipids generated by the activation of the phosphoinositide 3-kinase (PI3K) (3). PTEN is a major negative regulator of the PI3K/Akt signaling pathway (1,4,5). PTEN possesses a carboxy-terminal, noncatalytic regulatory domain with three phosphorylation sites (Ser380, Thr382, and Thr383) that regulate PTEN stability and may affect its biological activity (6,7). PTEN regulates p53 protein levels and activity (8) and is involved in G protein-coupled signaling during chemotaxis (9,10).

1. Cantley, L.C. and Neel, B.G. (1999) Proc Natl Acad Sci USA 96, 4240-5.
2. Myers, M.P. et al. (1997) Proc Natl Acad Sci USA 94, 9052-7.
3. Myers, M.P. et al. (1998) Proc Natl Acad Sci USA 95, 13513-8.
4. Wan X and Helman LJ (2003) Oncogene 22, 8205–11
5. Wu, X. et al. (1998) Proc Natl Acad Sci USA 95, 15587-91.
6. Vazquez, F. et al. (2000) Mol Cell Biol 20, 5010-8.
7. Torres, J. and Pulido, R. (2001) J Biol Chem 276, 993-8.
8. Freeman, D.J. et al. (2003) Cancer Cell 3, 117-30.
9. Funamoto, S. et al. (2002) Cell 109, 611-23.
10. Iijima, M. and Devreotes, P. (2002) Cell 109, 599-610.

Human research participants

Policy information about studies involving human research participants

Population characteristics	Supplementary tables 1-3 provide these characteristics in details. Please also see our supplementary methods section. Here are summaries:<br><br>All patients were men who underwent radical prostatectomy for localized prostate cancer<br><br>JHMI<br>Mean age, White men 60 years, Black men 58.3 years<br>Mean PSA (ng/mL), White men 9.8, Black men 10.8<br>72 (24%) men developed metastatic disease<br><br>TCGA<br>Mean age, White men 61.7 years, Black men 57.3 years<br>Mean PSA (ng/mL), White men 10.9, Black men 11.4<br>82 (17.5%) developed disease recurrence<br><br>DVA<br>Mean age, White men 2.7 years, Black men 59.9 years<br>Mean PSA (ng/mL), White men 8.8, Black men 10.6
Recruitment	Details on TCGA recruitment can be found at the main website. Individual contributing sites are responsible for adherence to guidelines set forth by the TCGA leadership. https://www.cancer.gov/about-nci/organization/ccg/research/structural-genomics/tcga/history/policies
Ethics oversight	Institutional Review Board was obtained previously from the Johns Hopkins Medical Institute and the Durham Veterans Affairs Medical Center. Details on TCGA ethics and oversight can be found at the main website. Individual contributing sites are responsible for oversight. https://www.cancer.gov/about-nci/organization/ccg/research/structural-genomics/tcga/history/policies

Note that full information on the approval of the study protocol must also be provided in the manuscript.

Clinical data

Policy information about clinical studies

All manuscripts should comply with the ICMJEguidelines for publication of clinical research and a completedCONSORT checklist must be included with all submissions.

Clinical trial registration	No clinical trial registrations. Data for JHMI and DVA cohorts were retrospectively collected. More information on TCGA cancer be found at the projects website https://www.cancer.gov/about-nci/organization/ccg/research/structural-genomics/tcga/history
Study protocol	As above, this information would not be available for JHMI and DVA. Details on TCGA can be found at their main website: https://www.cancer.gov/about-nci/organization/ccg/research/structural-genomics/tcga/history
Data collection	The discovery cohort was comprised of two groups of patients who underwent radical prostatectomy and no additional treatment until metastatic recurrence at Johns Hopkins Medical Institute (JHMI). The first group consisted of 355 intermediate- or high-risk patients treated between 1995 and 2005, of which 33 were Black. The second group consisted 143 Black men treated from 2006 to 2010. The DVA validation cohort consisted of men treated with radical prostatectomy for clinically localized PC between 1989 and 2016 at the DVA. For both of these cohorts, patient and clinically data were retrospectively collected from chart review.
Outcomes	Patients from JHMI were followed until the development of metastatic disease following radical prostatectomy. Notably, in this cohort, no patients received any adjuvant treatment following surgery. Metastatic disease development was assessed with imaging using computed tomography or bone scan following biochemical recurrence, as defined by a rise in PSA >0.2 ng/mL. In TCGA, patients were followed until any disease recurrence which included the earlier of development of metastatic disease as well as any biochemical recurrence. Included in multivariable analyses for metastasis-free survival was a validated genomic risk score for developing metastatic disease following treatment for localized prostatectomy based on transcriptomic signatures. In JHMI, 72 (24%) men developed metastatic disease and in TCGA 82 (17.5%) developed disease recurrence.<br><br>The primary analysis was conducted with metastasis-free survival in JHMI given its inherent strengths for outcomes as noted in our supplementary methods. Disease-free survival was used as to confirm findings from JHMI outcome analyses.
