# Supplementary material for: Plasma cells are enriched in localized prostate cancer in Black men and are associated with improved outcomes
Source: Nat Commun. 2021 Feb 10;12:935. doi: 10.1038/s41467-021-21245-w (PMC7876147; doi:10.1038/s41467-021-21245-w)
Supplement: Supplementary file 2 — Peer Review File [file 41467_2021_21245_MOESM2_ESM.pdf]

REVIEWER COMMENTS

Reviewer #1 (Remarks to the Author): Expert in prostate cancer and immunotherapy

Thank you for the opportunity to review "Plasma cells are enriched in prostate cancer from black men and are associated with enhanced immune responsiveness and improved cancer outcome." The aim of this study was to compare transcriptional profiles between primary prostate tumors from Black versus White men to identify differential immune signatures that could suggest distinct immune responses. This is a very interesting, hypothesis-generating study—however, the authors make several conclusive claims that cannot be supported by the methods employed within this manuscript.

Major Revisions
1. The title does not match the findings presented within this manuscript. There is no proof provided within the data section demonstrating that plasma cells are associated with enhanced immune responsiveness. Please revise the title unless they provide data from patients treated with sipuleucel-T, a FDA-approved autologous dendritic cell vaccine.

2. Sipuleucel-T is approved in the metastatic castration-resistant prostate cancer (mCRPC) setting. Furthermore, all of the patients with mCRPC received androgen deprivation therapy (ADT), which has been shown to have profound immunological effects within the prostate tumor microenvironment. Therefore, the authors should validate their findings using:
a. Prostate tissues in the mCRPC setting.
b. Lymph node metastases.

3. The transcriptional data points to an enrichment of plasma cells. For this reason, it is unclear why the authors chose to evaluate plasma cell density using IHC staining for CD79a, a pan-B cell marker. They should reevaluate their findings with CD138 IHC (Supplemental Figures 1 d-g).

4. Is the association between PC-B and increased plasma cell content independent of PTEN deletion status (frequency ~20% in primary and ~50% in CRPC), which has been shown to cause immune exclusion?

5. The authors should confirm their associations with TLS using IHC and/or multiplex immunofluorescence.

6. Is there a difference between Black versus White when evaluating plasma cell content by quartiles?

Minor Revisions
1. Authors should be specific in this journal that their use of "immunotherapy" is referring to sipuleucel-T (autologous dendritic cell vaccine) and not immune checkpoint blockade.

2. Line 19: "No signatures of immune-responsiveness exist for PC." This is contradicted in line 90 of the Methods section: "Increased expression of this IFNG signature was previously associated with response to immunotherapy for men with advanced PC." (Reference 3) Please clarify for consistency. Also, how does your signature compare to the one from Spranger et al. Nature. 2015 Jul 9;523(7559)?

3. Supplementary Tables 1-3: Would acknowledge that the cohorts are unbalanced by certain criteria (e.g. by grade group in the TCGA cohort, by organ-confined status in the GRID cohort), which is inevitable when only a small number of Black men are represented. This may skew the results.

4. Lines 154-156: "These data suggest the prognostic benefits of plasma cells in primary prostate cancer are likely related to activation of ADCC mediated by NK activity following tumor antigen recognition by plasma cells." This is a hypothesis, but there is no direct evidence here of tumor antigen recognition or ADCC. Please correct.

Reviewer #2 (Remarks to the Author): Expert in transcriptomics

The authors use multiple clinical prostate cancer cohorts to derive an hypothesis to help explain a potential improved response rate in African American (AA) over White men when receiving immunotherapy. Prostate cancer has show little response to immunotherapy as comparted to other cancer. The study does not provide any experimental confirmation of these proposed findings. The definition of AA ancestry is also variable between cohorts.

1) Defining the Cohorts. For the African American cohort how were the individuals defined? There appears to be an inconsistency in these definitions. The greatest concern would be using a transcriptomic signature to define the cohort (GRID) and then make comments about transcriptomics differences. Specifically, according to the methods supplement for the GRID cohort, "race was defined by a transcriptomic-based signature validated for expression data from primary PC tumours (Spratt et al. unpublished, ref 8))". This should be clarified as this is an unpublished study. How can this signature distinguish between such a highly diverse AA population given the high level of genomic admixture? Was this same signature applied to other cohorts for validation? Does the signature harbour the immune signature that was reported as a result? It would be important to demonstrate that the two signatures are independent of one another. (the unpublished paper is submitted by lists XX,XX as the number of tested cases).

2) The authors note that Sartor et al (Ref 4) propose a significantly better response in AA men over white men using spuleucel-T immunotherapy. Spuleucel-T immunotherapy was designed to activate the immune system against the prostatic acid phosphatase expressed on most prostate cancer cells. They also report a significantly higher PSA level in the AA men as compared to the white male population. Although, reported as non-significant, the PSA levels are also higher in the AA patients in the current study. Could PSA explain the differences in the immune profile?

3) A number of prior studies have explored for genomics differences between AA and white populations of men with prostate cancer. Lower incidence of PTEN mutations but also other alterations have been reported. If the authors include these genomic differences do, they also see an enrichment in their immune profile?

4) Bryc et al (Proc Natl Acad Sci U S A 2010 Jan 12;107(2):786-91) but also a number of other genomic studies suggest that AA men have up to 20% European ancestry (genomic admixture). Can the signature also be plotted using ethnicity traits derived from the WES data (e.g., TCGA) to show that these findings are related to African ancestry and not some other confounding parameter like PSA levels, size of prostate gland at time of surgery, or BPH?

Minor comments

-In the supplemental table 1, the JHH matching of 150 cases of AA and White men appears similar with all respects except for PSA levels. This is noted as not statistically significant. However, the difference between a mean (SD) of 9.8 (6.7) and 24.0 (114.0), for White and AA men, respectively, appear quite different. Is this correct?

-It is not clear how the TCGA clinical outcomes data was followed. This was not included in the initial study. Can the authors confirm that cases reporting no failure, in fact, were followed for biochemical failure?

Reviewer #3 (Remarks to the Author): Expert in survival analyses

The manuscript is original and contains potentially important information relevant to the immune

microenvironment found in prostate cancers. The distinctions between clinical outcomes in AA men and other ethnic subsets are important on many levels and new insights derived from this manuscript may contribute to our better understanding of the pathophysiology underlying these differential outcomes.

1) The measurements of IgG activity are indirect and more caution seems appropriate in the interpretation of these indirect measurements.

2) Does other evidence exist that IgG production might vary by racial subsets? If so, this should be referenced. Is this a general phenomenon are is this unique to prostate cancer? What about vaccination literature?

3) In Figure 1c, substantial differences in the overall immune content between TCGA and JHMI datasets appear to be present. Please comment on why this might be.

4) Importantly, the relationship between outcomes and plasma cells appear to be somewhat non-linear. In the figure 2C, the lowest two quartiles of plasma cells have no impact on outcomes. Only the highest quartiles appear to be distinct. Please comment on the non-linearity and compare to the TCGA data shown in figure 2a.

5) In the outcome curves shown in figure 2a (particularly TCGA) only the lowest quartile of plasma cells appeared distinct. Is this finding different from that of 2c?

6) Were the figures in 2c comprised of JHMI or TCGA data or both? Please clarify.

7) The relationship between plasma cells and ADCC should be framed as a hypothesis rather than a factual relationship. Though correlations are clear, causality is not.

Reviewer #4 (Remarks to the Author): Expert in B cells

This short report of Weiner and colleagues uses a bioinformatics approaches to examine the immune cell infiltrates in prostate cancers. They use newly developed tool to determine the race of samples and address the question of why in the US, black men with prostate cancer have a different outcomes to the white population, in terms of survival and the benefits of immune therapy. They find that black men have more plasma cells in their tumors, which may correlate with increased NK cell activity. This study fits in with an emerging body of evidence that B cells and plasma cells are key players in anti-tumor immunity.

Specific comments.
1. Major point. The authors need to provide some direct experimental evidence that plasma cells are increased in prostate cancer tumor mass. Immunohistochemistry for a plasma cell marker, probably a pan-anti-IgG is needed to validate the findings. Extended Figure 1d shows a B cell protein CD79a which is not really relevant for enumerating plasma cells.
2. Figure 2d (lines 140-144) The authors test whether IgG3-to-IgG1 switched cells are increased in prostate cancer. The basis of this assay it ref 13, however although that study found that the IgG3-to-IgG1 switch occurred in tumors, it didn’t find this event to be enriched in prostate cancer.
3. The text around lines 118-122 is not really accurate. Only a very low proportion of cells that secrete antibody proliferate. Plasma cells are post-mitotic, while plasmablasts do proliferate, although compared to B cell this is very minor. Also, plasma cells constitutively secrete Ab, and so there is not an activation state per se. The activation occurs in non-secreting B cell. Hence Ig production (of any isotype) and plasma cell numbers by definition correlate, making Extended figure 1j-k trivial.
4. The authors have not cited or discussed Shalapour et al Nature 2015. This study is highly relevant as it examined plasma cells in clinical samples of prostate cancer and conducts extensive analysis the role of B cells and plasma cells in mouse models of treatment resistance prostate cancer. The authors conclude that plasma cells are protecting the tumors in some contexts.
5. The data availability statement is vague. How is a reasonable request defined and what is Decipher Biosciences statement on the likelihood of granting permission? The authors should also

provide the code on the GitHub depository or similar.
6. Extended figure 1F needs to be enlarged to be readable.

REVIEWER COMMENTS

Reviewer #1 (Remarks to the Author): Expert in prostate cancer and immunotherapy

Thank you for the opportunity to review "Plasma cells are enriched in prostate cancer from black men and are associated with enhanced immune responsiveness and improved cancer outcome." The aim of this study was to compare transcriptional profiles between primary prostate tumors from Black versus White men to identify differential immune signatures that could suggest distinct immune responses. This is a very interesting, hypothesis-generating study—however, the authors make several conclusive claims that cannot be supported by the methods employed within this manuscript.

Major Revisions

1) The title does not match the findings presented within this manuscript. There is no proof provided within the data section demonstrating that plasma cells are associated with enhanced immune responsiveness. Please revise the title unless they provide data from patients treated with sipuleucel-T, a FDA-approved autologous dendritic cell vaccine.

Reply

We agree with the reviewer and we did reach out to Dendreon, the company who produces sipuleucel-T. We requested access to expression data from patients enrolled in clinical trials with sipuleucel-T. Unfortunately, our inquiry was never answered. Because our main findings were surrounding more plasma cells in the PC-B based on multiple orthogonal methods, we have used the phrase "enhanced immune infiltrate" in the title. We have adjusted our manuscript title to avoid misleading readers.

Line 1

Title: Plasma cells are enriched in prostate cancer from black men and are associated with enhanced immune infiltration and improved cancer outcome

2) Sipuleucel-T is approved in the metastatic castration-resistant prostate cancer (mCRPC) setting. Furthermore, all of the patients with mCRPC received androgen deprivation therapy (ADT), which has been shown to have profound immunological effects within the prostate tumor microenvironment. Therefore, the authors should validate their findings using:
a. Prostate tissues in the mCRPC setting.
b. Lymph node metastases.

Reply

Based on the reviewer's insightful point, we evaluated plasma cell content in mCRPC, and lymph nodes. We first looked at a very well annotated cohort of tumors from men with mCRPC. We obtained access via cBioPortal to data on a cohort of men with mCRPC and molecular profiling - the Robinson cohort first published in Cell in 2015.[2] This cohort is one of the most referenced and well-annotated cohorts of tumors of this type with a sizable number of mCRPC tumors with RNA expression data (n=118). We also reached out to the original authors of this paper and were able to obtain access to the self-identified race annotation. However, only 7 men were annotated as Black. Thus, while we saw the median plasma cell content was higher in PC-B, the analysis was

underpowered (see new supplemental figure below, p=0.3). Given these constraints, we don't feel it would be appropriate to make strong claims due to low numbers. Unfortunately, the lack of racial ancestry annotation and subjects with Black race within publicly available tumors cohorts is a major issue in cancer research (see **Figure 1b** in the accompanying manuscript on the creation of a transcriptomic model to define Black race in prostate cancer as well by Spratt et al.[3]). The only other comparable publicly available cohort of mCRPC with molecular profiling has only 88 tumors with RNA sequencing and only 5 tumors from Black men – and we suspect even fewer with RNA sequencing.[4] Race annotation is also not publicly available in that cohort. The dearth of PC-B tumors with molecular profiling was the ultimate inspiration for the accompanying manuscript and we are also currently working with collaborators and trialists to increase involvement of Black patients in molecular and clinical trial studies. Thus, for now, these are the best measurements in the mCRPC setting that can be offered. In our revised manuscript, we discuss this point and support the future direction for comparison of molecular profiles of mCRPC by race.

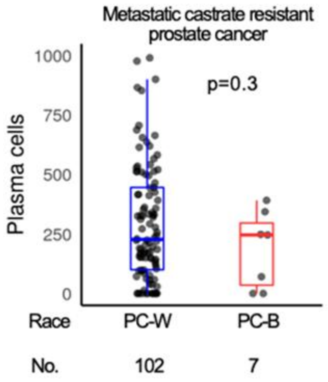

The reviewer has also asked that we assess plasma cell content in tumors from lymph node metastases. We believe the reviewers suggestion to do an analysis in lymph node metastases was made to attempt to correlate plasma content with more advanced disease.

We looked at the plasma cell content in a previously published cohort of 23 paired tumors from radical prostatectomy and lymph nodes in men who had tumor lymph node involvement at the time of radical prostatectomy at the University of California San Diego.[5] Race annotation was not available for this cohort. We found tumors in the lymph nodes had higher plasma cell content (See figure below, p=0.029). However, we feel that including this analysis in the manuscript could potentially be misleading as one would expect that lymph nodes have high immune content. Additionally, due to incomplete follow-up, only 20 men had information on biochemical recurrence with very limited duration of follow-up. Thus, outcomes could not be assessed in this cohort.

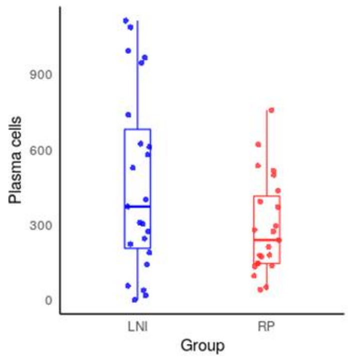

In summary, in response to this reviewer's request, we did analyze more advanced prostate cancer cases. We did find increased plasma cell content in tumors from Black men. However, due to the paucity of publicly available (n=7) tissue from Black men in this setting, we feel our comments on this topic should be tempered.

Line 132

We also sampled a large cohort of men with metastatic castrate-resistant PC (mCRPC; PC-B=7; PC-W=102; **Supplementary table 7**).[2] al Due to the low numbers of PC-B samples in the mCRPC cohort, no difference in plasma cell content by race was detected (**Extended figure 1m**).

Line 225

Additionally, extending these observations to the mCRPC disease state are limited as there were only seven mCRPC annotated as PC-B which is a common issue in race-based genomic analyses.[3] We support further evaluation of advanced PC within Black men to validate our observation in primary PC.

Extended figure 1m

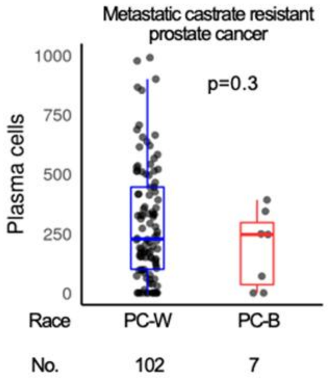

3) The transcriptional data points to an enrichment of plasma cells. For this reason, it is unclear why the authors chose to evaluate plasma cell density using IHC staining for CD79a, a pan-B cell marker. They should reevaluate their findings with CD138 IHC (Supplemental Figures 1 d-g).

Reply

We agree that a marker more specific to plasma cells would improve our IHC validation of our primary finding that PC-B has higher plasma cell content. We initially stained our cases with CD138 and observed patchy expression of CD138 in the benign prostate epithelium (predominantly basal cells) as well as in a small subset of tumor cells. This made making automated digital image quantification of CD138+ cells impossible (see arrowhead in new Extended **Figure 1e**). This distribution of CD138 (syndecan-1) in prostate epithelial cells is consistent with previously published work (see PMID 16280658). To circumvent this issue, we have now performed a blinded, manual quantification of CD138+ cell density in the same Kaur et al. cohort where we originally reported CD79a density. To validate this assay, we also examined the correlation of CD38 with CD138 (an alternative plasma cell-B-cell marker) in a subset of cases. We found a high correlation between these assays as expected (R2=0.39, p<0.0001). In summary, this additional IHC evaluation validates our findings and was statistically significant. We have included this analysis and found a similar increase in CD138 density in PC-B compared to PC-W (Wilcoxan-rank sum p<0.001)

Line 105

To further validate these findings, we leveraged tumors from the previously described Kaur et al.[6] cohort which showed PC-B possessed increased signals of B-lineage cells based on DNA methylation signatures (**Extended figure 1c)** and both CD79a+ and CD138+ cell density (**Extended figures 1d-g**).

Extended figure 1e

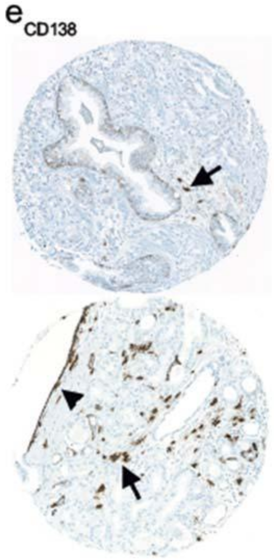

Extended figure 1f

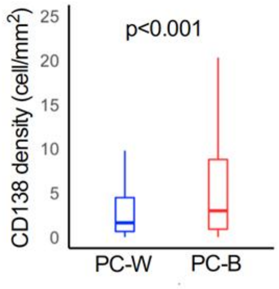

4) Is the association between PC-B and increased plasma cell content independent of PTEN deletion status (frequency ~20% in primary and ~50% in CRPC), which has been shown to cause immune exclusion?

Reply

This reviewer and reviewer 2 comment #3 bring up valuable points. Is the association between PC-B and plasma content due to the effect modification of some genomic alteration? Other reviewers bring up similar points regarding other clinicopathologic features. In the initial submission, we started to answer this question by assessing the interaction between *ERG* alterations and PC-B with plasma cell content in linear regression (**Extended figure 1q**) which showed no interaction. We now take that analysis several steps further. In the new Supplemental table 8, we now show the interaction between PC-B and *PTEN* loss in all cohorts is non-significant. In summary, the association between PC-B and plasma cell content is independent of *PTEN* loss.

Line 137

We next assessed several potential sources of effect modification for our main finding of increased plasma cell content in PC-B. The association between PC-B and increased plasma cell content was independent of rearrangements in *ERG* and *PTEN* loss, two of the most common genomic alterations in PC which are less common in PC-B,[7,8] (**Extended figure 1p** and **Supplemental table 8**).

5) The authors should confirm their associations with TLS using IHC and/or multiplex immunofluorescence.

Reply

This is an important point. In the initial submission, we showed that tumors with both increased plasma cell content and increased measures of a tertiary lymphoid structure (TLS) signature were further associated with increased markers of inflammation, IFNG, IgG expression, and PC-B. We used a previously validated expression-based marker of TLS for this initial analysis. As the reviewer has request, we now confirm these observations with IHC. More specifically, we assessed tumors with high and low TLS signature as a qualitative validation for this signature. The immunostains were done in a manner similar to previously published on this topic using IHC.[9] Adjacent slides are stained, merged, and pseudocolored to demonstrate the spatial relational between cell types. In each of the TLS high cases (three shown below), discrete lymphoid aggregates

of T- and B-cells with scattered adjacent CD138+ cells are demonstrated consistent with the presence of TLS. We feel these additional staining provide substantive support for the TLS signature in prostate tumors and our group has been encouraged by these new images to pursue detailed evaluation of TLS in ongoing work. To summarize, we provide new IHC supporting the TLS signature.

Lines 127

Immunohistochemical assessment of T-cells, B-cells and plasma cells in tumors with high TLS activity confirmed the presence of discrete lymphoid aggregates relative to tumors with low TLS activity (**Extended figure 1k-l**).

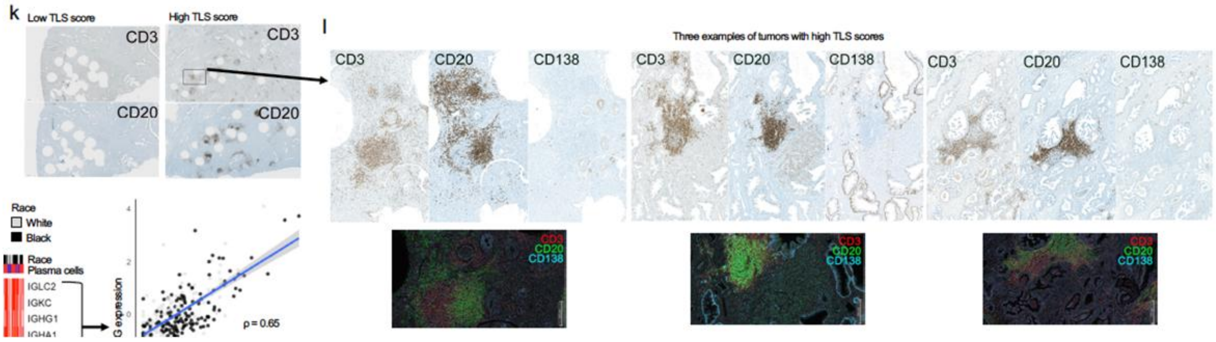

6) Is there a difference between Black versus White when evaluating plasma cell content by quartiles?

Reply

The reviewer brings up an important point. We associate PC-B with high plasma cell content and high plasma cell content (by quartile) with longer time to disease recurrence but we did not show that the highest plasma cell quartile was enriched with PC-B. We now provide two new supplemental tables to show the breakdown of race by plasma cell content in all three cohorts. In summary, these analyses show PC-B tended to be in the higher quartiles of plasma cell content.

Line 100

After deconvoluting tumor invading lymphocytes by cell type (**Figure 1c**), plasma cell content differed the greatest by race in JHMI and was the only cell type that differed by race in all cohorts (JHMI, TCGA, and DVA) with increased quantities in PC-B (**Figure 1d-e** and **supplemental tables 4-6**).

Supplemental table 4: Plasma cell content quartiles by race in JHMI

Plasma cell content (Quartile)	PC-W, n=150	PC-B, n=150
4 (Lowest)	52 (34.7%)	23 (15.3%)
3	42 (28.0%)	33 (22.0%)
2	32 (21.3%)	43 (28.7%)
1 (Highest)	24 (16.0%)	51 (34.0%)

Cochrane-Armitage test for trend, $p<0.001$

Supplemental table 5: Plasma cell content quartiles by race in TCGA

Plasma cell content (Quartile)	PC-W, n=410	PC-B, n=58
4 (Lowest)	105 (25.6%)	12 (20.7%)
3	112 (27.3%)	5 (8.6%)
2	105 (25.6%)	12 (20.7%)
1 (Highest)	88 (21.5%)	29 (50.0%)

Cochrane-Armitage test for trend, p<0.001

Supplemental table 6: Plasma cell content quartiles by race in DVA

Plasma cell content (Quartile)	PC-W, n=410	PC-B, n=58
4 (Lowest)	1062 (27.2%)	100 (13.4%)
3	1031 (26.4%)	131 (17.5%)
2	968 (24.8%)	194 (25.9%)
1 (Highest)	839 (21.5%)	323 (43.2%)

Cochrane-Armitage test for trend, p<0.001

Minor Revisions

7) Authors should be specific in this journal that their use of “immunotherapy” is referring to sipuleucel-T (autologous dendritic cell vaccine) and not immune checkpoint blockade.

Reply

We agree with this point and have adjusted the text accordingly.

Line 67

Black men die more often of prostate cancer yet, interestingly, may derive greater survival benefits from immune-based treatment with sipuleucel-T.

Line 141

In a prospective trial assessing the immune-based therapy sipuleucel-T, Black men with PC experienced longer overall survival compared to White men, in particular when baseline serum prostate-specific antigen (PSA) was low.[10]

Line 77

In one immunotherapy trial assessing sipuleucel-T, autologous dendritic cells sensitized to a PC, the greatest responses were seen in Black men.[10]

8) Line 19: “No signatures of immune-responsiveness exist for PC.” This is contradicted in line 90 of the Methods section: “Increased expression of this IFNG signature was previously associated with response to immunotherapy for men with advanced PC.” (Reference 3) Please

clarify for consistency. Also, how does your signature compare to the one from Spranger et al. Nature. 2015 Jul 9;523(7559)?

Reply

We agree with the reviewer; The phrasing is contradictory and the point is paramount to the motivation for this work. The referenced work (PMID 32238575) was a prospective trial assessing the association between several markers and response to immunotherapy targeting CTLA-4.This work did demonstrate increased IFNG signature activity was seen in tumors of patients who responded to immunotherapy and we determine this same signature should be used in our work. Notably, IFNG was not assessed formally as a biomarker predictive of a clinical outcome in PMID 32238575. The result from the referenced work was based on 19 patients and the patients who responded best to treatment were also substantially less exposed to previous prostate cancer treatments which may have ultimately confounded the causative link between IFNG and response to immunotherapy. Although our intent here is not to criticize the important work by that group, we do think the previous phrasing in the initial submission surrounding IFNG was overstated and may come off as misleading. For this reason, we have adjusted our phrasing to maintain a consistent narrative.

Regarding the actual signature used to measure IFNG activity, as stated above, the signature is the same as the referenced work in PMID 32238575. Also, as above, given the findings from that study in prostate cancer specifically, we felt this signature was appropriate for our work. The source of the signature is from the Cancer Hallmark Molecular Signatures Database[11] available at the link below and as cited in our Methods. This signature is based on the expression of 200 genes that are up-regulated in response to IFNG activity.

https://www.gsea-msigdb.org/gsea/msigdb/cards/HALLMARK_INTERFERON_GAMMA_RESPONSE

In the reference the reviewer provides on melanoma (PMID 25970248), in Extended Data Figures 4 and 6 of that work, it appears the IFNG signature is the expression of the gene *IFNG* based on qRT-PCR. In general, in our work we have favored an approach of using validated signatures of activity comprised of multiple gene expressions to add specificity to biological processes. For this reason and the background from PMID 32238575, we ultimately used the Hallmark IFNG signature for our work. In summary, we have adjusted our verbiage based on the suggestion from the reviewer.

Line 110

In a prospective trial of 19 men with advanced PC, tumors responsive to anti-CTLA-4 treatment were enriched with enhanced markers of inflammation and interferon gamma (IFNG) prior to treatment.[12]

Line 350

From the Hallmark Gene Set Collection, we also derived a measure of genes upregulated during chronic and acute inflammation (HALLMARK_INFLAMMATORY_RESPONSE) and interferon gamma (IFNG; HALLMARK_INTERFERON_GAMMA_RESPONSE).[11] Increased expression of this

IFNG signature was previously shown to be enriched in tumors that responded to anti-CTLA-4 systemic therapy in men with advanced PC.[12]

9) Supplementary Tables 1-3: Would acknowledge that the cohorts are unbalanced by certain criteria (e.g. by grade group in the TCGA cohort, by organ-confined status in the GRID cohort), which is inevitable when only a small number of Black men are represented. This may skew the results.

Reply

We agree with the reviewer on this point and do note that the use of multiple cohort for validation of study capitalizes on the inherent strengths of each which may be limitations in others. In this case, only the JHMI cohort was grade and stage matched. We now provide a new supplement in our methods regarding the strengths and limitation of each cohort including the point about TCGA and DVA having unbalanced tumor characteristics. Please see our response to Reviewer 2 Comment #1 with regards to replacing the GRID cohort with DVA which is based on patient-identified race.

In summary, we have included the following text in the supplemental methods to address this comment:

Line 40 of Supplemental methods

Similarly, unlike JHMI, this cohort was not matched on clinical covariates based on race.

Line 53 of Supplemental methods

Also, unlike JHMI, this cohort was not matched on clinical covariates based on race.

10) Lines 154-156: "These data suggest the prognostic benefits of plasma cells in primary prostate cancer are likely related to activation of ADCC mediated by NK activity following tumor antigen recognition by plasma cells." This is a hypothesis, but there is no direct evidence here of tumor antigen recognition or ADCC. Please correct.

Reply

We agree with the reviewer and acknowledge these exploratory analyses were meant as hypothesis-generating assessments and are currently the focus of investigations in our group. This includes our findings related to associating plasma cell content and IgG expression with NK activity which is an eventual mediator of ADCC as a source of anti-tumor activity. Please note that Reviewer #3 comments 1 and 7 expressed similar concerns regarding overstating findings from this analysis. In summary, to address both this comment and the comments from Reviewer #3, we have made relevant adjustments to the text as below.

Lines 184

These data suggest future studies are indicated to assess the role of plasma cell-activated ADCC mediated by NK activity in tumor progression for primary PC.

Lines 215

These tumors demonstrated increased levels of NK cell activity suggesting plasma cells may augment ADCC within the TME, a mechanism that warrant future validation.

Reviewer #2 (Remarks to the Author): Expert in transcriptomics

The authors use multiple clinical prostate cancer cohorts to derive an hypothesis to help explain a potential improved response rate in African American (AA) over White men when receiving immunotherapy. Prostate cancer has show little response to immunotherapy as comparted to other cancer. The study does not provide any experimental confirmation of these proposed findings. The definition of AA ancestry is also variable between cohorts.

1) Defining the Cohorts. For the African American cohort how were the individuals defined? There appears to be an inconsistency in these definitions. The greatest concern would be using a transcriptomic signature to define the cohort (GRID) and then make comments about transcriptomics differences. Specifically, according to the methods supplement for the GRID cohort, “race was defined by a transcriptomic-based signature validated for expression data from primary PC tumours (Spratt et al. unpublished, ref 8))”. This should be clarified as this is an unpublished study. How can this signature distinguish between such a highly diverse AA population given the high level of genomic admixture? Was this same signature applied to other cohorts for validation? Does the signature harbour the immune signature that was reported as a result? It would be important to demonstrate that the two signatures are independent of one another. (the unpublished paper is submitted by lists XX,XX as the number of tested cases).

Reply

The reviewer is correct that Black race was defined differently in each of the cohorts we assessed. We understand that this reviewer could have concerns about the transcriptomic signature given that the race model has not yet been published. We have high confidence that the model is highly predictive of Black race. That manuscript is presently under the peer review process and thus regrettably we do not feel that we can fully present/republish the entire methodology in this work. We had hoped to have the race model accepted prior to this submission. We did discuss this issue with the Editor and now include an alternate strategy of using a second validation cohort which utilized patient-identified race.

More specifically, we have decided to replace the GRID validation cohort with an alternate large cohort which distinguishes race by patient-identification from the Durham Veterans Affairs Hospital (DVA; n=538).[1]

All the expression-based findings previously validated in GRID were confirmed in DVA and our figures and tables have been updated. We would like to reassure the reviewer that all the observations made in the discovery (JHMI) were found to be present in both DVA (and GRID). . Please note that race is patient-defined in JHMI (discovery cohort) and based on genomic ancestry in TCGA (our first validation cohort).

We discuss the advantage of using patient-identified and genomic-based race in our new supplemental methods. Please also see below for the changes made to our methods section clarifying race assignment. In summary, we have removed the GRID

cohort which relied on the transcriptomic race model to assign race and we have replaced it with a new large validation cohort which uses patient-identified race.

N.B.: Because of this reviewer's interest in our transcriptomic race model, we are including a confidential draft of that manuscript for their review. (Spratt et al.)

Line 237

The discovery cohort was comprised of two groups of patients who underwent radical prostatectomy and no additional treatment until metastatic recurrence at Johns Hopkins Medical Institute (JHMI) with patient-identified race.

Line 249

Our first validation cohort was generated from publicly available data within TCGA PRAD dataset (n=468). Race in this cohort was defined by genetic ancestry (410 White and 58 Black).[13]

Line 255

To further confirm findings from JHMI, we used a second, large retrospective cohort with whole transcriptomic data (DVA; n=538).[1] Race was patient-identified in this cohort (236 White and 302 Black).

2) The authors note that Sartor et al (Ref 4) propose a significantly better response in AA men over white men using spuleucel-T immunotherapy. Spuleucel-T immunotherapy was designed to activate the immune system against the prostatic acid phosphatase expressed on most prostate cancer cells. They also report a significantly higher PSA level in the AA men as compared to the white male population. Although, reported as non-significant, the PSA levels are also higher in the AA patients in the current study. Could PSA explain the differences in the immune profile?

Reply

This is an insightful comment from the reviewer. In response to this and other reviewer comments, we have created a new supplemental table expanding the number of interaction analyses we performed to assess for effect modifiers. Please see the response to the next comment from this reviewer for more details on the genomic alteration interaction analyses. Specific to this comment, we performed a linear regression for plasma cell content adjusting for race, PSA, and an interaction term between race and PSA. To summarize, in both JHMI and DVA, there was no significant interaction between race and increasing PSA suggesting PSA was not an effect modifier on the association between race and plasma cell content.

Supplemental table 8: Interaction analyses

Cohort	Variable	Estimate of interaction with Black race (95% CI)	p
JHMI, n=268	PTEN loss (Immunohistochemistry)	-41.67 (117.10 to 33.76)	0.3

TCGA, n=468	PTEN loss (Homozygous deletion)	-193.94 (-408.97 to 21.08)	0.077
DVA, n=467	PTEN loss (RNA-based signature)	19.22 (-137.37 to 175.81)	0.8
JHMI, n=298	PSA (Per increase by 10 ng/mL)	4.26 (-37.73 to 46.25)	0.8
DVA, n=522	PSA (Per increase by 10 ng/mL)	11.51 (-26.0 to 49.0)	0.5
JHMI, n=300	Age quartile		
	1 (Youngest)	Reference	
	2	-59.53 (-149.24 to 30.18)	0.193
	3	-2.73 (-85.78 to 80.32)	0.9
	4 (Oldest)	-61.99 (-145.11 to 21.14)	0.143
DVA, n=538	Age quartile		
	1 (Youngest)	Reference	
	2	13.87 (-69.64 to 97.38)	0.7
	3	-3.62 (-84.28 to 77.05)	0.9
	4 (Oldest)	-89.30 (-165.07 to 4.48)	0.063
TCGA, n=320	Subtype		
	ERG	Reference	
	ETV1	50.18 (-224.72 to 325.09)	0.7
	ETV4	-38.54 (-413.6 to 336.52)	0.8
	FLI1	129 (-284.06 to 542.07)	0.5
	SPOP	-46.62 (-212.9 to 119.67)	0.6
	FOXA1	-278.9 (-660.55 to 102.76)	0.151
	IDH1	-371.81 (-808.37 to 64.76)	0.095
	**Other**	**159.58 (17.62 to 301.55)**	**0.028**

Multiple multivariable linear regressions were performed for the outcome of plasma cell content. Included in each regression was race (White vs Black), the variable listed in this table, and an interaction between the respective variable and Black race. Patients were excluded from these analyses for missing data or if PTEN loss based on RNA-based signature was “Indeterminate” in the DVA cohort. Bolding indicates statistical significance at p<0.05.

Line 141

In a prospective trial assessing the immune-based therapy sipuleucel-T, Black men with PC experienced longer overall survival compared to White men, in particular when baseline serums prostate-specific antigen (PSA) was low.[10] Additionally, previous work has also noted enhanced immunoglobulin production in Black patients following vaccinations most notably at younger ages.[14,15] Here, the association between PC-B and increased plasma cell content was independent of baseline PSA and age (**Supplemental table 8**).

3) A number of prior studies have explored for genomics differences between AA and white populations of men with prostate cancer. Lower incidence of PTEN mutations but also other alterations have been reported. If the authors include these genomic differences do, they also

see an enrichment in their immune profile?

Reply

This is an important from the reviewer and, in fact, our group was among the first to note many of these differences.[8] As discussed in our response to the previous comment from this reviewer, our revised manuscript contains a number of new interaction analyses to assess for effect modifiers. This analyses assess rearrangements in *ERG*, *PTEN* loss, PSA, age, and molecular subtype based on TCGA Research Network analysis of prostate cancer in 2016 published in Cell.[16] We found no effect modification due to *ERG* rearrangements, *PTEN* loss, PSA, or age. We did note there was an interaction between the "Other" subtype or the non-subtypeable tumors and PC-B for the outcome of plasma cell content (Estimate +159.58, 95% CI 17.62 to 301.55, p=0.028; see **Supplemental table 8** reproduced in our response to this reviewer's previous comment). This was a very interesting finding given PC-B is more often categorized as "other" non-subtypeable prostate cancer, probably owing to the relative rarity of PC-B in TCGA.[7] This finding suggests the association between PC-B and increased plasma cell content was most prominent in tumors that were designated as not able to be subtyped. Indeed, we found 14 of the 16 (87.5%) of PC-B listed as "Other" subtype had above-median plasma cell content. We now list these patient ID's as a new supplemental table. We now also provide the list of mutated genes and copy number alterations in these 14 patients. To summarize, no gene is mutated more than twice and no gene is amplified or deep deleted more than three times in this cohort. This finding suggests perhaps "Other" non-subtypeable prostate cancer may be further classified based on the tumor microenvironment and immune content as opposed to genomic alterations. We have now updated own manuscript to reflect these findings and encourage future work investigating tumors denoted as the "Other" subtype which comprises >25% of primary prostate cancer in TCGA. We are very grateful to the reviewer for this insight suggestion as our findings have the potential to stimulate important future work on this topic.

In summary, we now assess genomic and clinicopathologic factors as potential effect modifiers of our main finding of increased plasma cell content in PC-B. We found the association between PC-B and increased plasma cell content was enhanced in "Other" non-subtypeable tumors which suggest these tumors may be further classified by their tumor microenvironment and immune content as opposed to genomic alterations.

Line 137

We next assessed several potential sources of effect modification for our main finding of increased plasma cell content in PC-B. The association between PC-B and increased plasma cell content was independent of rearrangements in *ERG* and *PTEN* loss, two of the most common genomic alterations in PC which are less common in PC-B,[7,8] (**Extended figure 1n-o** and **Supplemental table 8**). In a prospective trial assessing the immune-based therapy sipuleucel-T, Black men with PC experienced longer overall survival compared to White men, in particular when baseline serum prostate-specific antigen (PSA) was low.[10] Additionally, previous work has also noted enhanced immunoglobulin production in Black patients following vaccinations most notably at younger ages.[14,15] Here, the association between PC-B and increased plasma cell content was independent of baseline PSA and age (**Supplemental table 8**). Finally, since the frequency of PC molecular subtypes differ by race,[7] we assessed the interaction between subtype and race on plasma cell content in TCGA. We found the

association between PC-B and increased cell content was significantly enhanced among tumors classified as “other” non-subtypeable tumors (Estimate of interaction +159.58, 95% CI 16.62 to 301.55, p=0.028; **Supplemental table 8**). Accordingly, of the 16 PC-B classified as “other” non-subtypeable PC, 14 (87.5%) were found have to above median plasma cell content (**Supplemental table 9**). Among these 14 tumors, no single gene was amplified or deep deleted more than three time and no gene was mutated more than twice (**Supplemental data 1-2**). Since, “other” non-subtypeable PC comprises about 26% of tumors in TCGA and PC-B is more likely to not be classified as “other” non-subtypeable PC,[7,16] these finding suggest a substantial portion of “other” non-subtypeable PC-B may be defined by their tumor immune microenvironment.

4) Bryc et al (Proc Natl Acad Sci U S A 2010 Jan 12;107(2):786-91) but also a number of other genomic studies suggest that AA men have up to 20% European ancestry (genomic admixture). Can the signature also be plotted using ethnicity traits derived from the WES data (e.g., TCGA) to show that these findings are related to African ancestry and not some other confounding parameter like PSA levels, size of prostate gland at time of surgery, or BPH?

Reply

In our original submission, we evaluated enrichment of immune infiltrate in Black men. We originally utilized three distinct methodologies to assign race. First, we used a large cohort of individuals who patient-identified race (JHMI), second, we used, as suggested above, genomic ancestry from TCGA as previously described.[13] Third we used an additional cohort of individuals with transcriptomic-based race classified.

As noted in our response to the first comment from this reviewer, we have decided to remove the data from GRID given the peer-review process/publication of the manuscript detailing the transcriptomic race model is slightly behind this manuscript. We have since added data from a new cohort from the Durham Veterans Affair Hospital (DVA; PC-B=302; PC-W=236) - see comment Reviewer 2 Comment #1.[1] In each cohort (JHMI, TCGA, and DVA), we found enrichment of immune infiltrate, plasma cell content, and IFNG signaling in PC-B in all cohorts. (see **Figure 1b-g**).

To specifically answer the reviewer’s question, we do find that with WES-defined African ancestry there is enrichment of all these signatures. We showed this in the original submission of the manuscript (Figure 1e) but now do so in a clearer fashion and have, for other reviewers, updated the methods to more clearly state how race was assigned (see comment Reviewer 2 Comment #1 for updates to our methods which clarify how race was defined)

In addition, we now perform several new linear regressions with interactions terms to assess for potential effect modification (see response to comment #2 from this reviewer). From one of these regression, we note that increasing PSA did not modify the effect of race on plasma cell content and was not independently associated with plasma cell content. Additionally, for the reviewer’s reference, we performed an analysis of prostate weight and plasma cell content. Prostate weight was available in 128 of the 300 samples in JHMI (Range 25-180gm). In a univariable linear regression, the Log(prostate weight in grams) was not associated with plasma cell content (Estimate 10.0, 95% Confidence interval -63.6 to 83.7, p=0.8). We did not check for effect modification with race since only 32 of the 150 PC-B had information on tumor weight. Prostate weight is not reported in the other cohorts.

In summary, our plasma cell signature was increased in tumors from Black men defined by patient-identified race and genomic ancestry. The association between PC-B and plasma cell content was independent of PSA.

Minor comments

5) In the supplemental table 1, the JHH matching of 150 cases of AA and White men appears similar with all respects except for PSA levels. This is noted as not statistically significant. However, the difference between a mean (SD) of 9.8 (6.7) and 24.0 (114.0), for White and AA men, respectively, appear quite different. Is this correct?

Reply

We are grateful to the reviewer for noticing this point. Upon further analysis, we discovered two PC-B tumors were labeled as having PSA values of 999. After conferring with the original curator of these data (author F.A.F.), these PSA values were not available because they were not in the electronic medical records at the time of retrospective inquiry to compile the data. We now provide the median values of PSA only for men with known PSA values. The median PSA was 9.8 for White men and 10.8 for Black men. We apologize for this error. Please note that we have redone each our multivariable Cox-regression excluding these two patients. The results have not been altered appreciably and for the reviewer's reference, one of the two patients was lost to follow-up after 1 year and the other was lost to follow-up after 7 years. Neither patient experienced metastatic recurrence. In summary, we have fixed the error which accounts for the issue raised by the reviewer on this point.

Supplemental table 1: Johns Hopkins Medical Institute cohort

Characteristic		White n = 150	Black n = 150	p
Age				0.015
	Mean (SD)	60.0 (5.7)	58.3 (6.2)	
Grade Group				0.060
	2	49 (32.7%)	42 (28%)	
	3	42 (28%)	60 (40%)	
	4	24 (16%)	27 (18%)	
	5	35 (23.3%)	21 (14%)	
PSA, ng/mL				1.0
	Mean (SD)	9.8 (6.7)	10.8 (8.1)	
	Unknown	0 (0%)	2 (1.3%)	
Organ Confined				0.9
	Yes	110 (73.3%)	110 (73.3%)	
	No	40 (26.7%)	40 (26.7%)	
Margin +				0.145
	No	110 (73.3%)	96 (64%)	
	Yes	40 (26.7%)	52 (34.7%)	

Unknown	0 (0%)	2 (1.3%)

Abbreviations: SD, standard deviation; PSA, prostate specific antigen

6) It is not clear how the TCGA clinical outcomes data was followed. This was not included in the initial study. Can the authors confirm that cases reporting no failure, in fact, were followed for biochemical failure?

Reply

The reviewer is correct, the citation for TCGA prostate cancer does not detail the manner of follow-up clearly. We (and many others) determined failure using cBioPortal disease-free survival (DFS) as defined as the development of metastatic disease or as any biochemical recurrence. Thus, within the TCGA there are inherent limitations with lack of homogeneous follow-up regimens since DFS would necessarily be determined as time of detection of disease recurrence as opposed to the actual timing of recurrence. Since death and prostate-cancer-specific death was so uncommon on TCGA,[17] the best recognized outcomes among scientist utilizing this resource is to define clinical failure as metastatic recurrence and biochemical recurrence with the former being a much more prognostic surrogate endpoint.[18] Since TCGA documents either metastatic or biochemical recurrence as disease recurrence, the heterogeneity in this outcome is a limitation to outcome evaluations in TCGA.

This is why we conducted the main survival analyses in JHMI which has very rigorous and tightly defined clinical outcomes which was defined as metastatic recurrence. Thus, we use TCGA only as an independent validation of the main outcome evaluation in JHMI.

In response to this and another reviewer comment, we now have a new supplemental methods section on the strengths and limitations of each dataset and note that the combined use of these three unique and independent datasets with unique strengths and limitations helps to validate findings in each and increase the likely external validity of findings in this study. We have also updated our figures to better label metastasis-free survival versus disease-free survival.

In summary, we have updated our methods and figures to more clearly describe the follow-up for clinical outcomes in TCGA and JHMI. We can confirm TCGA reports either metastasis-free survival or biochemical recurrence-free survival while JHMI reports metastasis-free survival.

Lines 3 in Supplemental methods

JHMI strengths include follow-up assessment. All patients were uniform in that none received adjuvant treatment prior to any disease recurrence. Since all patients where managed at a single institution, the follow-up regimens for disease monitoring were likely similar. Finally, data on metastatic recurrence was available as opposed to biochemical recurrence which is less predictive of death from prostate cancer.[18,19]

Lines 32 in Supplemental methods

Limitations of TCGA include the clinical follow-up. In TCGA, disease-free survival (DFS) is defined as the earlier of development of metastatic disease or biochemical recurrence. Since death and prostate-cancer-specific death was so uncommon on TCGA,[17] the best outcomes to use for failure are metastatic recurrence and biochemical recurrence with the former being a much more prognostic surrogate endpoint for death.[18] Additionally, TCGA is comprised of patients from multiple institutions with likely heterogeneous follow-up regimens which would likely contribute to bias in terms of recurrence detection depending on the frequency of disease monitoring. Patients in TCGA may have also received adjuvant treatment prior to a disease recurrence which ultimately may have impacted the timing of recurrence.

Reviewer #3 (Remarks to the Author): Expert in survival analyses

The manuscript is original and contains potentially important information relevant to the immune microenvironment found in prostate cancers. The distinctions between clinical outcomes in AA men and other ethnic subsets are important on many levels and new insights derived from this manuscript may contribute to our better understanding of the pathophysiology underlying these differential outcomes.

1) The measurements of IgG activity are indirect and more caution seems appropriate in the interpretation of these indirect measurements.

Reply

As we note earlier to Reviewer #1 comment 10 and below to comment 7 from this reviewer, we agree and acknowledge our assessments of IgG and the association between our signature of IgG and plasma cell content with NK cell activity were designed as exploratory analyses to generate hypotheses and encourage future directions which are the focus of current ongoing work within our group. We have made substantial alterations to the text in the context of the limitations of these observational data. Importantly, we now refer to our signature as IgG "expression" as opposed to IgG "production" throughout the manuscript to further add transparency to the limitation of this signature. To summarize, the revised manuscript provides a more cautious interpretation of the IgG signature based on its inherent limitations.

Line 116

In the setting of increased IFNG activity, IgG class-switch is augmented.[20] With this in mind, we would expect tumors with high plasma cell content would also have increased markers of IgG expression. We found IgG expression correlated highly with plasma cell content (Spearman's correlation coefficient = 0.65) suggesting tumors with high plasma cell content also have high IgG expression, potentially as a reflection of plasma cell antibody secretion (**Extended figure 1i-j**).

2) Does other evidence exist that IgG production might vary by racial subsets? If so, this should be referenced. Is this a general phenomenon are is this unique to prostate cancer? What about vaccination literature?

Reply

We have updated our manuscript to better frame our findings related to IgG in the context of previous work on racial differences in immunoglobulin production. We did note previous work showing Black patients produced higher antibody responses to both rubella[15] and influenza vaccines.[14] Additionally, Black patients are more likely to experience graft failure following organ transplantation suggestive of more robust immune response to the graft.[21] These data, like ours, cannot determine whether these racial differences in antibody-mediated immune activity are related to qualities intrinsic to people of Black race or if they are related to differential exposures based on the social construct of race. We do note from these works that the trend noted in the study regarding the influenza vaccine was only observed in young (aged 35-45 years) patients and the rubella vaccine study was conducted in children. We thus sought to determine if age was an effect modifier on the association between PC-B and plasma cell content. To do this we, split the JHMI and DVA cohorts into quartiles of age and performed a linear regression which three covariates, age by increasing quartile, race (PC-W vs PC-B) and an interaction term between race and age by quartiles. No age quartile and none of the interaction terms were statistically significant while race was still independently associated with plasma cell content. Due to comments from other reviewers, we now include this interaction analysis as well others in a supplemental table assessing the potential of effect modification of multiple other relevant variables. Please see the reply to Reviewer 2 comments #2 and #3 for the complete text detailing the manuscript revisions.

In summary, based on prior existing evidence from vaccination literature, we now provide an assessment of the age as an effect modifier of plasma cell content in PC-B. The association between increased plasma cell content in PC-B was independent of age.

Line 141

In a prospective trial assessing the immune-based therapy sipuleucel-T, Black men with PC experienced longer overall survival compared to White men, in particular when baseline serums prostate-specific antigen (PSA) was low.[10] Additionally, previous work has also noted enhanced immunoglobulin production in Black patients following vaccinations most notably at younger ages.[14,15] Here, the association between PC-B and increased plasma cell content was independent of baseline PSA and age (**Supplemental table 8**).

Supplemental table 8: Interaction analyses

Cohort	Variable	Estimate of interaction with Black race (95% CI)	p
JHMI, n=268	PTEN loss (Immunohistochemistry)	-41.67 (117.10 to 33.76)	0.3
TCGA, n=468	PTEN loss (Homozygous deletion)	-193.94 (-408.97 to 21.08)	0.077
DVA, n=467	PTEN loss (RNA-based signature)	19.22 (-137.37 to 175.81)	0.8
JHMI, n=298	PSA (Per increase by 10 ng/mL)	4.26 (-37.73 to 46.25)	0.8
DVA, n=522	PSA (Per increase by 10 ng/mL)	11.51 (-26.0 to 49.0)	0.5
JHMI, n=300	Age quartile		
	1 (Youngest)	Reference	
	2	-59.53 (-149.24 to 30.18)	0.193

		3	-2.73 (-85.78 to 80.32)	0.9
		4 (Oldest)	-61.99 (-145.11 to 21.14)	0.143
DVA, n=538	Age quartile			
		1 (Youngest)	Reference	
		2	13.87 (-69.64 to 97.38)	0.7
		3	-3.62 (-84.28 to 77.05)	0.9
		4 (Oldest)	-89.30 (-165.07 to 4.48)	0.063
TCGA, n=320	Subtype			
	ERG		Reference	
	ETV1		50.18 (-224.72 to 325.09)	0.7
	ETV4		-38.54 (-413.6 to 336.52)	0.8
	FLI1		129 (-284.06 to 542.07)	0.5
	SPOP		-46.62 (-212.9 to 119.67)	0.6
	FOXA1		-278.9 (-660.55 to 102.76)	0.151
	IDH1		-371.81 (-808.37 to 64.76)	0.095
	**Other**		**159.58 (17.62 to 301.55)**	**0.028**

Multiple multivariable linear regressions were performed for the outcome of plasma cell content. Included in each regression was race (White vs Black), the variable listed in this table, and an interaction between the respective variable and Black race. Patients were excluded from these analyses for missing data or if PTEN loss based on RNA-based signature was "Indeterminate" in the DVA cohort. Bolding indicates statistical significance at p<0.05.

3) In Figure 1c, substantial differences in the overall immune content between TCGA and JHMI datasets appear to be present. Please comment on why this might be.

Reply

This is an important point raised by the reviewer. The height of the bars from figure 1c indicate the mean overall immune content from tumors of White and Black men in each of the three cohorts. In each of the cohort, there was enriched immune content in PC-B. But it is an astute observation that immune content appears to be less in JHMI versus TCGA. We believe that this difference in total immune content is because of difference in tissue procurement and handling. More specifically, the immune content signature is derived from an RNA expression-based signature as described in Yoshihara et al.[22] As detailed in the methods, the source tissues for the DVA and JHMI cohorts were derived from formalin-fixed paraffin embedded (FFPE) specimens while tissue from TCGA is derived from fresh-frozen (FF) specimens.[16] As noted in previous works, the gene expression may be decreased as measured in FFPE specimens compared to that of FF tissue yet are still considered reliable.[23]

For the reviewer's knowledge, we would like to point out that in light of this comment, we looked at the percentage of immune content that is plasma cells and assessed differences in race in all three cohorts. This would normalize the plasma cell marker between the three cohorts by assessing percentage. We found that plasma cells as a percentage of the entire tumor immune content was increased in Black vs White men in

JHMI (9.2% vs 5.9%, Wilcoxon Rank Sum $p<0.001$), TCGA (14.0% vs 11.9%, Wilcoxon Rank Sum $p<0.001$), and DVA (8.6% vs 10.8%, Wilcoxon Rank Sum $p<0.001$).

The three cohorts are each unique with their own strengths and limitations. Some, such as differences in tissue handling, are known and inherent to the databases. For instance, JHMI has the highest quality data on outcomes with excellent follow-up time and known data on metastatic recurrence. Additionally, JHMI had enough data on Black men grade- and stage-matched to White men to power the discovery portion of this study. Yet, as noted above, the expression signatures are likely diminished in JHMI due to the time between fixation and RNA extraction. TCGA offers independent validation of the findings in JHMI with the added data on genomic and methylomic analyses despite containing few Black men. The strengths of DVA include its large number of included tumors to further validate findings. The use of these three unique and independent datasets with unique strengths and weaknesses helps to validate findings in each and increase the likely external validity of findings in this study. We have expanded on all these points in our revised manuscript in a new supplemental methods section.

To summarize, the differences in the overall immune content between the three cohorts are related to tissue procurement and handling which would change the absolute value of RNA-expression without changing the relative differences between tumors within cohorts. These points are noted in our supplemental methods.

Line 13 in Supplemental methods

Limitations include the tissue procurement and handling. The source tissues for JHMI were derived from formalin-fixed paraffin embedded (FFPE) specimens which were several years old prior to RNA extraction. As noted in previous works, the absolute value of gene expression measurements may be decreased in FFPE specimens compared to that of fresh-frozen (FF) tissue yet are still considered reliable and show similar relative differences to cohorts with higher quality gene expression data.[23] This accounts for the relative discrepancies in the heights of the bars in **Figure 1c** which is an expression-based measurement of tumor immune content. Other limitations include lack of genetic data within JHMI.

Line 29 in Supplemental methods

Finally, TCGA RNA extraction occurred from FF tissue. Thus, the RNA expression levels are the least attenuated of the three cohorts.

Line 56 in Supplemental methods

Finally, similar to JHMI, the source tissues for DVA were derived from formalin-fixed paraffin embedded (FFPE) specimens with RNA extraction performed several years following fixation. As noted above, this would account for lower absolute value gene expression but not relative gene expression within each tumor and within each cohort.

4) Importantly, the relationship between outcomes and plasma cells appear to be somewhat non-linear. In the figure 2C, the lowest two quartiles of plasma cells have no impact on outcomes. Only the highest quartiles appear to be distinct. Please comment on the non-linearity and compare to the TCGA data shown in figure 2a.

Reply

This is an important point from the reviewer. Without a biologically justified cutoff for plasma cell content, we chose to assess the association of plasma cell and CD8+ T-cell content based on quartiles of content. As the reviewer notes, the highest two quartiles in JMHI and the top three quartiles in TCGA tended to differ in terms of longer failure-free survival. As described below and in response to Reviewer 2 comment #6, comparing survival outcomes between JHMI and TCGA should be limited to confirming trends in JHMI due the inherent limitations to outcomes in TCGA. For this reason, we were encouraged by the similar finding in TCGA of longer failure-free survival in tumors with higher plasma cell content. We also note the reviewer brings up an excellent point regarding the non-linear relation between plasma cell content and failure-free survival. We now acknowledge in the discussion section of the manuscript this point and note this will be the focus of ongoing work as this biomarker is scrutinized in ongoing prospective evaluations. In summary, we now comment on the non-linear relationship between plasma cell content and survival outcomes. We have also expanded our discussion of how follow-up was assessed in TCGA in our supplemental methods.

Line 223

Notably, a relationship between disease recurrence and plasma cell content was not completely linear (**Figure 2a-b**) which should encourage future work determining a manner to measure and ascertain a clinically relevant cutoff for plasma cell content.

5) In the outcome curves shown in figure 2a (particularly TCGA) only the lowest quartile of plasma cells appeared distinct. Is this finding different from that of 2c?

Reply

We apologize for the lack of clarity with these data. We would like to note that in TCGA only data on disease-free survival is available which may present as metastatic or biochemical recurrence (indicated by a rise in serum PSA). As noted in response to Reviewer 2 comment #6, we have updated our methods section to more clearly describe the follow-up in TCGA which is greatly limited by heterogeneous outcome failure (disease-free), shorter follow-up (28.2 months for those who did not fail as opposed to 7 years in JHMI), and various follow-up regimens (compared to JHMI where every patient was treated and managed at one institution). These aspects of TCGA render that dataset more limited as a source of outcome analyses compared to JHMI. As we detail in our new methods section on the strengths and limitations of each dataset, one strength of JHMI is that each patient was followed until metastatic recurrence without any adjuvant treatment following radical prostatectomy. For these reasons, we utilize TCGA as a source of validation for the findings from JHMI for failure after surgery. Because of the limitations of TCGA, we did not include a multivariable analysis survival outcomes in the initial submission. TCGA DFS data was used only in figure 2a in the Kaplan-Meier curves. The p-values from these two assessments of CD8+ T-cells and plasma cells were from log-rank tests. We now label figures 2b,c,f, and j more clearly as assessing metastasis-free survival in JHMI. We also now provide the multivariable Cox regression for TCGA with regards to plasma cell and CD8+ T-cell content as supplemental figures. As expected, increasing CD8+ T-cell content was not associated with longer DFS and neither was increasing plasma cell content in this analysis although the HR's for plasma cell content confirmed a trend towards longer DFS in TCGA.

To summarize, we have updated our figures and manuscript to more clearly indicate which cohort was used for survival analyses. We now include a Cox regression from TCGA. We also discuss in more details the limitations of outcome assessment in TCGA in our supplemental methods.

Line 164

Even after adjusting for clinical covariates, CD8+ T-cells remained non-significant, while increasing plasma cell content was associated with longer metastasis-free survival (**Figure 2b and extended figures 2a-b**).

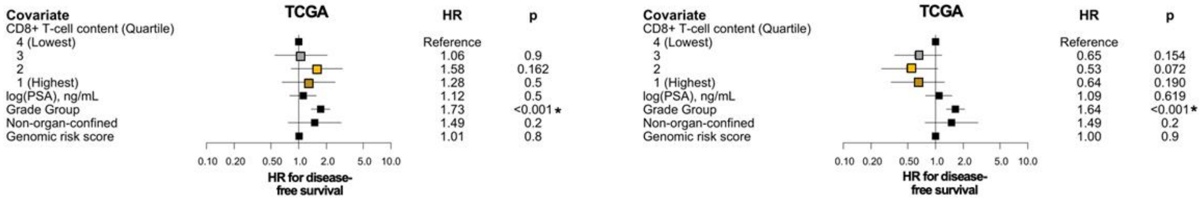

6) Were the figures in 2c comprised of JHMI or TCGA data or both? Please clarify.

Reply

As noted in our response to previous comment from this reviewer, we apologize for this confusion and we have updated the labels in figure 2 to clarify that the Cox regressions were all performed in JHMI and we now include in extended figure 2 the multivariable cox regressions for TCGA as noted in the previous comment response.

7) The relationship between plasma cells and ADCC should be framed as a hypothesis rather than a factual relationship. Though correlations are clear, causality is not.

Reply

We agree with this comment and as note in comment 1 from this reviewer and comment 10 from Reviewer #1, we have made substantial adjustments to our discussions surrounding IgG and ADCC to better frame these observations as associations and correlations and do not suggest we prove causality.

Line 184

These data suggest future studies are indicated to assess the role of plasma cell-activated ADCC mediated by NK activity in tumor progression for primary PC.

Line 215

These tumors demonstrated increased levels of NK cell activity suggesting plasma cells may augment ADCC within the TME, a mechanism that warrant future validation.

Reviewer #4 (Remarks to the Author): Expert in B cells

This short report of Weiner and colleagues uses a bioinformatics approaches to examine the

immune cell infiltrates in prostate cancers. They use newly developed tool to determine the race of samples and address the question of why in the US, black men with prostate cancer have a different outcomes to the white population, in terms of survival and the benefits of immune therapy. They find that black men have more plasma cells in their tumors, which may correlate with increased NK cell activity. This study fits in with an emerging body of evidence that B cells and plasma cells are key players in anti-tumor immunity.

Specific comments.

1. Major point. The authors need to provide some direct experimental evidence that plasma cells are increased in prostate cancer tumor mass. Immunohistochemistry for a plasma cell marker, probably a pan-anti-IgG is needed to validate the findings. Extended Figure 1d shows a B cell protein CD79a which is not really relevant for enumerating plasma cells.\\

Reply

We are grateful to this comment and the similar comment #3 from Reviewer 1. Please see below the new data produced similarly to the previous work on CD79a density in the original submission. These show that quantitative density of CD138+ cells was greater in PC-B. Since CD138+ is more specific to plasma cells,[24] these data along with our many other forms of orthogonal validation substantiate our main finding of increased plasma cell content in PC-B. Please see our response to Reviewer 1 comment #3 for our complete reply on this matter. Regarding additional assessment of IgG, other reviewers have commented on our assessment and verbiage surrounding our IgG signature. We have changed our verbiage surrounding the IgG signature and no longer call it a marker of "IgG production" and now instead refer to it as a marker of "IgG expression." As noted in response to comment #3 from this reviewer and reviewer 1 comment #3, we more clearly note the role of plasma cells and IgG production leading to increased ADCC should be the focus of future work and not proven by our study.

In summary, we now provide direct evidence of increase plasma cell content in PC-B using immunohistochemistry for CD138. We have also modified our verbiage regarding the IgG signature and ADCC such that we acknowledge future experimental work is needed to further characterize a mechanism for plasma cells antitumorigenic properties in prostate cancer.

Line 105

To further validate these findings, we leveraged tumors from the previously described Kaur et al.[6] cohort which showed PC-B possessed increased signals of B-lineage cells based on DNA methylation signatures (**Extended figure 1c)** and both CD79a+ and CD138+ cell density (**Extended figures 1d-g**).

Extended figure 1e and 1g

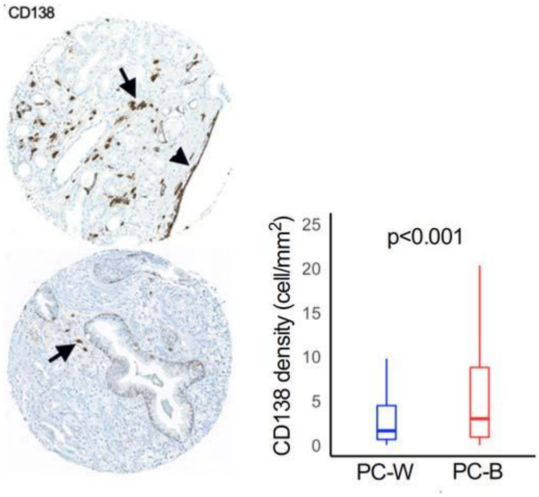

Line 116

In the setting of increased IFNG activity, IgG class-switch is augmented.[20] With this in mind, we would expect tumors with high plasma cell content would also have increased markers of IgG expression. We found IgG expression correlated highly with plasma cell content (Spearman's correlation coefficient = 0.65) suggesting tumors with high plasma cell content also have high IgG expression, potentially as a reflection of plasma cell antibody secretion (**Extended figure 1i-j**).

Lines 184

These data suggest future studies are indicated to assess the role of plasma cell-activated ADCC mediated by NK activity in tumor progression for primary PC.

Lines 215

These tumors demonstrated increased levels of NK cell activity suggesting plasma cells may augment ADCC within the TME, a mechanism that warrant future validation.

2. Figure 2d (lines 140-144) The authors test whether IgG3-to-IgG1 switched cells are increased in prostate cancer. The basis of this assay it ref 13, however although that study found that the IgG3-to-IgG1 switch occurred in tumors, it didn't find this event to be enriched in prostate cancer.

Reply

Thank you for bringing this to our attention. We would like to clarify the implications of this analysis. The reviewer is correct, relative to other tumor types, reference PMID 30742113 did not show substantial differences in IgG3-to-IgG1 subclass switch recombination in prostate tumor relative to benign prostate tissue.[25] In our analysis we are comparing IgG3-to-IgG1 switch exclusively from tumors (not tumors versus benign as in PMID 30742113).

More specifically, we present enrichment of plasma cell content and IgG expression in tumors with IgG3-to-IgG1 switch and note a higher proportion of tumors from Black men have IgG3-to-IgG1 switch in the tumor microenvironment.

We now make this point clearer in our manuscript and specifically note this is an "observation" to be transparent with the fact this was not an experimental finding. To summarize, in the revised manuscript we make it clearer the association between IgG3-to-IgG1 switch and tumors with high plasma cell content and IgG expression suggests these tumors have high plasma cell content and IgG expression as a result of B-cell antigen recognition and subsequent clonal expansion in the tumor microenvironment.

Lines 168

When B-cells encounter a foreign antigen, they undergo clonal expansion and immunoglobulin class-switch recombination. The most common class-switch recombination noted in multiple tumors types is IgG3 to 1 which is associated with cancer outcomes and can be measured by a signature originally described in an analysis by Hu et al.[25] In tumors from TCGA with any IgG3 to 1 class-switch recombination there were greater quantities of plasma cell content and more IgG expression, and a greater prevalence among PC-B (**Figure 2d and Extended Figure 2c)**. This observation suggests increasing plasma cell content and IgG expression may be reflective of B-cell antigen recognition within the TME

3. The text around lines 118-122 is not really accurate. Only a very low proportion of cells that secrete antibody proliferate. Plasma cells are post-mitotic, while plasmablasts do proliferate, although compared to B cell this is very minor. Also, plasma cells constitutively secrete Ab, and so there is not an activation state per se. The activation occurs in non-secreting B cell. Hence Ig production (of any isotype) and plasma cell numbers by definition correlate, making Extended figure 1j-k trivial.

Reply

We apologize for the confusion with the wording in this section. We have substantially modified the text to incorporate this reviewer's comments. We do think the extended figures regarding the IgG signature still contributes to the manuscript and thus we will leave it in the extended figure section. Please see our response to this reviewer's next comment as it is closely related to this one.

In summary, we have updated the verbiage in the manuscript to more accurately describe the relevance of the IgG signature based on the comment from this reviewer.

Lines 116

In the setting of increased IFNG activity, IgG class-switch is augmented.[20] With this in mind, we would expect tumors with high plasma cell content would also have increased markers of IgG expression. We found IgG expression correlated highly with plasma cell content (Spearman's correlation coefficient = 0.65) suggesting tumors with high plasma cell content also have high IgG expression, potentially as a reflection of plasma cell antibody secretion (**Extended figure 1i-j**).

Extended fig 1-j

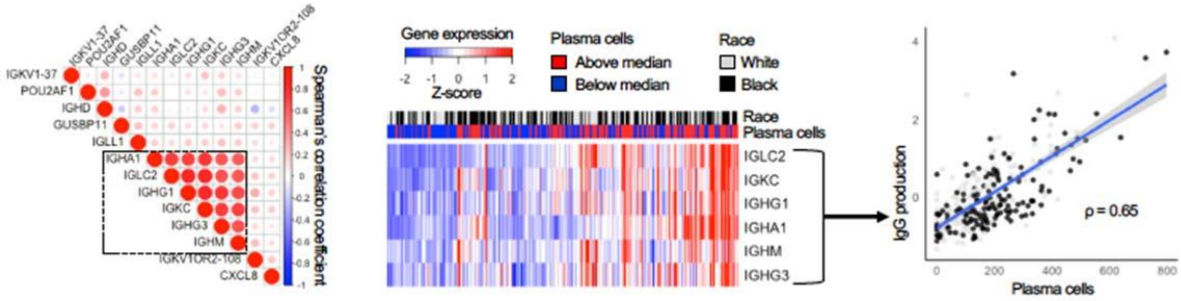

4. The authors have not cited or discussed Shalapour et al Nature 2015. This study is highly relevant as it examined plasma cells in clinical samples of prostate cancer and conducts extensive analysis the role of B cells and plasma cells in mouse models of treatment resistance prostate cancer. The authors conclude that plasma cells are protecting the tumors in some contexts.

Reply

In hindsight, we regret not including this paper given its relevance and the fact it was the first to show and characterize a role of plasma cells and B-cells in prostate cancer treatment response.

The main findings of this work published in 2015 are based on a murine model of advanced prostate cancer.[26] Following oxaliplatin treatment, there were detectable levels of 138+/IgA+ and induced treatment resistance in this model. In murine models replete of B-cells, the treatment resistance diminished. The authors thus characterize how immunosuppressive plasma cells can impede an anti-tumor T-cell response. These immunosuppressive plasma cells express IgA, IL-10 and PD-L1. They also noted in 24 metastatic or treatment-resistant tumors from humans contained more 138+/IgA+ cells than that seen in localized tumors.

Our current study is different from Shalapour et al. First, we explore primary prostate tumors only in human sample and second, we examined outcomes related to race. More specifically, we found primary prostate tumors from Black men have more immune content in the tumor microenvironment due to increased quantities of plasma cells and increased plasma cell content is associated with higher levels of IFNG, higher levels of NK cell activity, and longer time to disease recurrence following radical prostatectomy. Thus, our work in addition to others [27–29] in the context of the work from Shalapour et al.[26] support the general concept that B-cells in the tumor microenvironment are heterogeneous in terms of their protumorigenic or antitumorigenic roles with these divergent roles likely included by stage, grade and possibly race.[30]

One way to reconcile both observations would be to consider a heterogeneous role of B-cells in tumor microenvironments related to the inflammatory cytokine milieu in the microenvironment. For example, it is established that antibody isotype switch is driven by different local inflammatory cytokines in the microenvironment. IgA is mostly found in the intestines, predominantly driven by immune suppressive cytokine such as iNOS and IL-10[31,32] whereas switch to IgG can be driven by IFNG.[33,34] Shalapour et al., for instance, cite a review by Cerutti et al. which details several relevant inflammatory

factors that induce IgA class switch.[31] In the new supplemental figure below, we show the expression of these individual factors do not correlate with plasma cell content in primary prostate cancer and, as demonstrated by the original Figure 1g (reproduced in the previous comment reply), plasma cell content does correlate positively with IFNG. Therefore, the study from Shalapour et al. and our current study are likely assessing different tumor microenvironment environments and thus should be expected to have different Ig isotype and, potentially, different associated clinical outcomes. We now make this point in our revised manuscript and are thankful for the reviewer's insight on this point.

In summary, our manuscript now references the work by Shalapour et al. and suggests the heterogenous antitumorigenic/protumorigenic roles of B-cells and plasma cells may be related to differences in the inflammatory cytokine milieu in the tumor microenvironment.

Lines 188

Previous work from Shalapour et al. demonstrated 138+/IgA+ positive tumors were associated with immune-related treatment resistance and characterized how immunosuppressive plasma cells can impeded an anti-tumor T-cell response.[26] Thus, findings from the current work, other previous work,[27–29] and that of Shalapour et al.[26] suggest B-cells in the prostate TME are heterogeneous in terms of their protumorgenic or antitumorgenic roles.[30] One consideration to reconcile the potential heterogeneous roles of B-cells may be related the inflammatory cytokine milieu in the TME. Antibody isotype switch is driven by different local inflammatory cytokine milieu in the microenvironment. For example, IgA is mostly found in the intestines, predominantly driven by immune suppressive cytokine such as iNOS and IL-10[31,32] whereas switch to IgG is mostly driven by IFNG.[33,34]

As noted above, plasma cell content was positively associated with IFNG in primary PC (**Figure 1g**). Conversely, plasma cell content was not strongly correlated with the expression of several relevant inflammatory factors that induce IgA class switch (**Extended figure 2f**).[31] Thus, discrepancies in the TME inflammatory cytokine milieu leading to differential Ig isotype switching may account for the heterogeneous observed associations between plasma cell and PC outcomes.

Extended figure 2f

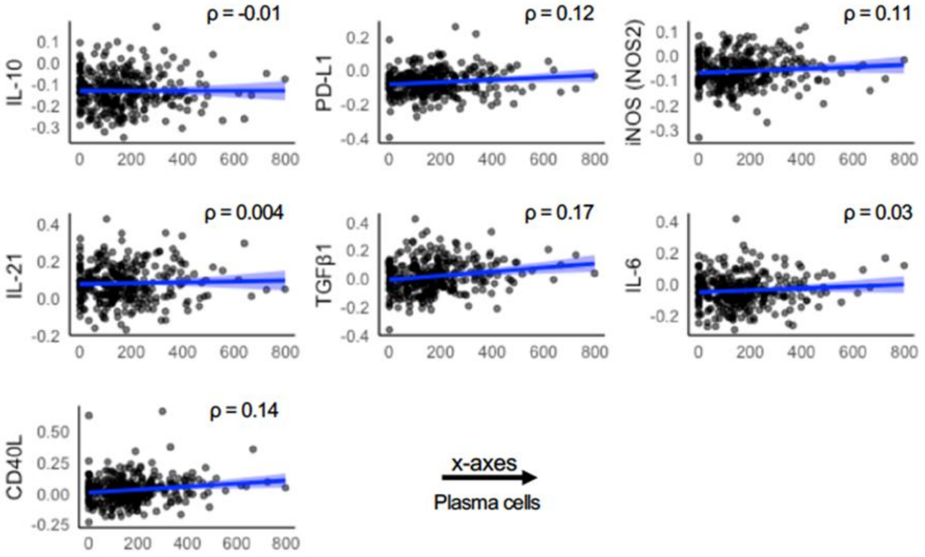

5. The data availability statement is vague. How is a reasonable request defined and what is Decipher Biosciences statement on the likelihood of granting permission? The authors should also provide the code on the GitHub depository or similar.

Reply

We agree with the reviewer. We have now uploaded the JHMI and DVA expression data to GEO which will be made available at the time of study publication. Please see Reviewer 2 comment #1 regarding replacing the original second validation cohort (GRID) with the cohort from the Durham Veterans Affairs Hospital (DVA). We also now provide a "Source data" excel document with all the data required to recreate our figures and regressions. This is a requirement for manuscripts published at *Nature Communications*. Additionally, all code in this study are based on previous publications available publicly and all citations have been detailed in the methods.

In summary, expression data from JHMI and DVA have been uploaded to GEO and a source data excel document has been provided to recreate all figures and regressions.

Line 425

Data availability: The data that support the findings of this study from the Johns Hopkins Medical Institute and Durham Veterans Affairs Hospital were deposited on NCBI Gene Expression Omnibus (GEO) and are accessible through GEO Series accession number GSE153352 and GSE157547, respectively. The source data underlying Figures 1a-k, 2a-j, Extended figures 1a-c, Extended figures 1f-j, Extended figures 1m-q, Extended figures 2a-b, Extended figures 2d-f, and Supplemental table 9 are provided as a Source Data file.

6. Extended figure 1F needs to be enlarged to be readable.

Reply

Thank you for point this out. We have decided to removed that figure in question in this revision due to the new inclusion of the CD138 density analysis.

References

1. Howard, L. E. *et al.* Validation of a genomic classifier for prediction of metastasis and prostate cancer-specific mortality in African-American men following radical prostatectomy in an equal access healthcare setting. *Prostate Cancer and Prostatic Diseases* 1–10 (2019) doi:10.1038/s41391-019-0197-3.
2. Robinson, D. *et al.* Integrative Clinical Genomics of Advanced Prostate Cancer. *Cell* **161**, 1215–1228 (2015).
3. Spratt, D. E. *et al.* Racial/Ethnic Disparities in Genomic Sequencing. *JAMA Oncol* **2**, 1070–1074 (2016).
4. Quigley, D. A. *et al.* Genomic Hallmarks and Structural Variation in Metastatic Prostate Cancer. *Cell* **174**, 758-769.e9 (2018).
5. Lee, H. J. *et al.* Evaluation of a genomic classifier in radical prostatectomy patients with lymph node metastasis. *Research and Reports in Urology* vol. 8 77–84 https://www.dovepress.com/evaluation-of-a-genomic-classifier-in-radical-prostatectomy-patients-w-peer-reviewed-article-RRU (2016).
6. Kaur, H. B. *et al.* Association of Tumor Infiltrating T-cell Density with Molecular Subtype, Racial Ancestry and Clinical Outcomes in Prostate Cancer. *Mod Pathol* **31**, 1539–1552 (2018).
7. Yuan, J. *et al.* Integrative comparison of the genomic and transcriptomic landscape between prostate cancer patients of predominantly African or European genetic ancestry. *PLOS Genetics* **16**, e1008641 (2020).
8. Tosoian, J. J. *et al.* Prevalence and Prognostic Significance of PTEN Loss in African-American and European-American Men Undergoing Radical Prostatectomy. *European Urology* **71**, 697–700 (2017).

9. Kroeger, D. R., Milne, K. & Nelson, B. H. Tumor-Infiltrating Plasma Cells Are Associated with Tertiary Lymphoid Structures, Cytolytic T-Cell Responses, and Superior Prognosis in Ovarian Cancer. *Clin Cancer Res* **22**, 3005–3015 (2016).

10. Sartor, O. *et al.* Survival of African-American and Caucasian men after sipuleucel-T immunotherapy: outcomes from the PROCEED registry. *Prostate Cancer and Prostatic Diseases* 1–10 (2020) doi:10.1038/s41391-020-0213-7.

11. Liberzon, A. *et al.* The Molecular Signatures Database Hallmark Gene Set Collection. *cels* **1**, 417–425 (2015).

12. Subudhi, S. K. *et al.* Neoantigen responses, immune correlates, and favorable outcomes after ipilimumab treatment of patients with prostate cancer. *Science Translational Medicine* **12**, (2020).

13. Yuan, J. *et al.* Integrated Analysis of Genetic Ancestry and Genomic Alterations across Cancers. *Cancer Cell* **34**, 549-560.e9 (2018).

14. Kurupati, R. *et al.* Race-related differences in antibody responses to the inactivated influenza vaccine are linked to distinct pre-vaccination gene expression profiles in blood. *Oncotarget* **7**, 62898–62911 (2016).

15. Haralambieva, I. H. *et al.* Associations between race, sex and immune response variations to rubella vaccination in two independent cohorts. *Vaccine* **32**, 1946–1953 (2014).

16. Abeshouse, A. *et al.* The Molecular Taxonomy of Primary Prostate Cancer. *Cell* **163**, 1011–1025 (2015).

17. Liu, J. *et al.* An Integrated TCGA Pan-Cancer Clinical Data Resource to Drive High-Quality Survival Outcome Analytics. *Cell* **173**, 400-416.e11 (2018).

18. Xie, W. *et al.* Metastasis-Free Survival Is a Strong Surrogate of Overall Survival in Localized Prostate Cancer. *JCO* **35**, 3097–3104 (2017).

19. Xie, W. *et al.* Event-Free Survival, a Prostate-Specific Antigen–Based Composite End Point, Is Not a Surrogate for Overall Survival in Men With Localized Prostate Cancer Treated With Radiation. *JCO* JCO.19.03114 (2020) doi:10.1200/JCO.19.03114.

20. Vazquez, M. I., Catalan-Dibene, J. & Zlotnik, A. B cells responses and cytokine production are regulated by their immune microenvironment. *Cytokine* **74**, 318–326 (2015).

21. Morris, A. A. *et al.* Influence of Race/Ethnic Differences in Pre-Transplantation Panel Reactive Antibody on Outcomes in Heart Transplant Recipients. *Journal of the American College of Cardiology* **62**, 2308–2315 (2013).

22. Yoshihara, K. *et al.* Inferring tumour purity and stromal and immune cell admixture from expression data. *Nature Communications* **4**, 1–11 (2013).

23. Hedegaard, J. *et al.* Next-Generation Sequencing of RNA and DNA Isolated from Paired Fresh-Frozen and Formalin-Fixed Paraffin-Embedded Samples of Human Cancer and Normal Tissue. *PLOS ONE* **9**, e98187 (2014).

24. Kambham, N., Kong, C., Longacre, T. A. & Natkunam, Y. Utility of Syndecan-1 (CD138) Expression in the Diagnosis of Undifferentiated Malignant Neoplasms: A Tissue Microarray Study of 1,754 Cases. *Applied Immunohistochemistry & Molecular Morphology* **13**, 304–310 (2005).

25. Hu, X. *et al.* Landscape of B cell immunity and related immune evasion in human cancers. *Nature Genetics* **51**, 560–567 (2019).

26. Shalapour, S. *et al.* Immunosuppressive plasma cells impede T-cell-dependent immunogenic chemotherapy. *Nature* **521**, 94–98 (2015).

27. Cabrita, R. *et al.* Tertiary lymphoid structures improve immunotherapy and survival in melanoma. *Nature* **577**, 561–565 (2020).

28. Petitprez, F. *et al.* B cells are associated with survival and immunotherapy response in sarcoma. *Nature* **577**, 556–560 (2020).

29. Helmink, B. A. *et al.* B cells and tertiary lymphoid structures promote immunotherapy response. *Nature* **577**, 549–555 (2020).

30. Tsou, P., Katayama, H., Ostrin, E. J. & Hanash, S. M. The Emerging Role of B Cells in Tumor Immunity. *Cancer Res* **76**, 5597–5601 (2016).

31. Cerutti, A. The regulation of IgA class switching. *Nature Reviews Immunology* **8**, 421–434 (2008).

32. Kunisawa, J. *et al.* Microbe-dependent CD11b + IgA + plasma cells mediate robust early-phase intestinal IgA responses in mice. *Nature Communications* **4**, 1772 (2013).

33. Coffman, R. L., Savelkoul, H. F. & Lebman, D. A. Cytokine regulation of immunoglobulin isotype switching and expression. *Semin. Immunol.* **1**, 55–63 (1989).

34. Finkelman, F. D., Katona, I. M., Mosmann, T. R. & Coffman, R. L. IFN-gamma regulates the isotypes of Ig secreted during in vivo humoral immune responses. *J. Immunol.* **140**, 1022–1027 (1988).

REVIEWER COMMENTS

Reviewer #1 (Remarks to the Author):

The revisions made here generally support the authors' tempered conclusions. The authors have addressed our concerns by investigating a small cohort of mCRPC tumors from Black men, evaluating the enrichment of plasma cells by CD138 IHC and TLS by CD20/CD138 IHC, assessing for an interaction between increased plasma cell content and PTEN loss, and making the suggested clarifications in the text. The following points remain:

1. In the title, it remains unclear what is meant by "enhanced immune infiltrate." As the authors have shown an association with improved metastasis-free survival and disease-free survival specifically with increased plasma cells but not CD8 T cells, it would be more accurate to revise the title as: "Plasma cells are enriched in localized prostate cancer in Black men and are associated with improved cancer outcomes"
2. Line 132: It is inaccurate to claim causality that no difference in plasma cell content by race was detected due to the low numbers of PC-B samples in the mCRPC cohort, as it is possible that there truly is no difference. Would refine this statement to say that it "may be due to the low numbers…"
3. Line 232: There is insufficient evidence to "imply B cell and plasma cells in the TME may play a more important role than CD8+ T cells in activating the tumor immune response." Please correct.
4. Please proofread the text for typographical errors.

Reviewer #2 (Remarks to the Author):

The authors have made major revisions and addressed my queries.

Reviewer #3 (Remarks to the Author):

There is currently no comment about clinical outcomes in the DBA cohort and some clarity should be provided on this issue particularly as the overall survival measurements are likely quite valid.why is this cohort excluded from survival analysis?

The burden of neoantigens was calculated but no data are presented. This in fact could be quite informative and brief mention should be added as to how this varies by race. This is important when alluding to issues such as potential responsiveness to immune therapies.

It should be made more clear that grade group one was excluded. This is an incredibly important subset of men particularly among those that have had PSA screening.

A simple grade group analysis is missing and one is left to wonder whether or not plasma cells and IgG correlate with grade. Certainly these data can be presented.

The distinction between plasma cell content and outcome is distinctly nonlinear in the JHMI and TCGA cohorts. This should be clearly pointed out as the current conclusion appears to be somewhat simplistic (especially when grade group one patients were excluded).

Reviewer #4 (Remarks to the Author):

the authors have done an excellent job with the revision of this paper and I am now in favour of publication as is

REVIEWER COMMENTS

Reviewer #1 (Remarks to the Author):

The revisions made here generally support the authors' tempered conclusions. The authors have addressed our concerns by investigating a small cohort of mCRPC tumors from Black men, evaluating the enrichment of plasma cells by CD138 IHC and TLS by CD20/CD138 IHC, assessing for an interaction between increased plasma cell content and PTEN loss, and making the suggested clarifications in the text. The following points remain:

1) In the title, it remains unclear what is meant by "enhanced immune infiltrate." As the authors have shown an association with improved metastasis-free survival and disease-free survival specifically with increased plasma cells but not CD8 T cells, it would be more accurate to revise the title as: "Plasma cells are enriched in localized prostate cancer in Black men and are associated with improved cancer outcomes"

Reply

We agree with the reviewer. To reduce the vagueness in the title, we have adopted the title proposed by the reviewer.

Line 1

Title: Plasma cells are enriched in localized prostate cancer in Black men and are associated improved cancer outcomes

2) Line 132: It is inaccurate to claim causality that no difference in plasma cell content by race was detected due to the low numbers of PC-B samples in the mCRPC cohort, as it is possible that there truly is no difference. Would refine this statement to say that it "may be due to the low numbers…"

Reply

We agree with the reviewer on this point and have updated the text based on their recommendations.

Line 135

No difference in plasma cell content by race was detected, which may be due to the low numbers of PC-B (**Extended figure 1m**).

3) Line 232: There is insufficient evidence to "imply B cell and plasma cells in the TME may play a more important role than CD8+ T cells in activating the tumor immune response." Please correct.

Reply

To avoid overstating our study's finding, we have altered the text based on this reviewer's comment.

Line 221

These tumors demonstrated increased levels of NK cell activity suggesting plasma cells may augment ADCC within the TME, a mechanism that warrants future validation. Together, these findings imply B-cells and plasma cells in the TME may play an important role in prostate tumor immune response.

4) Please proofread the text for typographical errors.

Reply

We apologize for any errors. Following this revision, multiple authors took the time to re-read the draft and assess for typographical errors.

Reviewer #2 (Remarks to the Author):

1) The authors have made major revisions and addressed my queries.

Reply

Thank you for taking the time to review our work.

Reviewer #3 (Remarks to the Author):

1) There is currently no comment about clinical outcomes in the DBA cohort and some clarity should be provided on this issue particularly as the overall survival measurements are likely quite valid.why is this cohort excluded from survival analysis?

Reply

We would like to thank the reviewer for allowing us to expand on our rationale for use of the DVA cohort. We used the DVA cohort due to the substantial numbers of PC-B tumors with RNA expression data (n=302) which is a rare resource in prostate cancer research. We specifically did not include clinical outcome measures in this manuscript because the DVA has limitations which, in our opinion, preclude meaningful interpretations:

1) Predominately lower risk cohort with few metastatic recurrences
2) High and inconsistent use of post-surgery treatments which impact time to recurrence

The DVA cohort consists of largely favorable-risk prostate cancer. The cohort was 73% (393/538) grade group 1 or 2 prostate cancer. Metastatic recurrence for men is low for these men: 0-3% for grade group 1 and 5-8% for grade group 2 or 3 in large series at 10 years of follow-up (the median follow-up in DVA was about 8 years).[1,2] Thus, metastatic events are expected to be low in DVA. In JHMI, we excluded grade group 1 as this is now considered to be non-surgical indolent cancer managed with surveillance per international guidelines.[3–5] In contradistinction to DVA, JHMI was enriched for high grade (grade group 4 or 5) disease (36%,107/300). With DVA consisting of only 12% (64/538)

high grade cases. Accordingly, only 5.4% (29/538) of DVA developed metastatic disease. This value is low relative to metastatic recurrence in JHMI (24%) and disease recurrence in TCGA (17.5%). Because of the very low clinical events in this cohort, we did not feel that an examination of it for clinical outcomes would be meaningful.

Additionally, there was also inconsistent and high use of additional treatments following surgery in the DVA cohort which we felt would also preclude any meaningful conclusions from a survival analysis. The observations in this manuscript, including our survival analyses, are based on the JHMI cohort which consisted of men diagnosed with prostate cancer who underwent initial treatment with surgery (radical prostatectomy) and then were observed until metastatic recurrence. One of the key features of this cohort that make it so informative related to clinical outcomes is that there were no treatments between the primary surgical event and detection of metastasis. This cohort is referred broadly within the prostate cancer community as "The Nature History Cohort."[6]

In contrast, the DVA cohort utilized inconsistent post-surgical treatments that render it a more limited resource for time-to-event analyses. More specifically, about 25% of men received immediate hormonal therapy following surgery which is known to delay biochemical recurrence and metastatic events. Furthermore, hormonal therapy was disproportionally used within the cohort: 35% of patients with tumors in the lowest quartile of plasma cell content and 25% of patients with tumors in the highest quartile of plasma cell content. Similarly, there was disproportionate use of additional post-surgical radiotherapy: 60% for the lowest quartile of plasma cell content and 45% for the highest quartile. These additional treatments, their timing following surgery, and duration and dosing would influence clinical outcomes assessments in indeterminable ways. For this additional reason, we did not include survival analyses for DVA.

Previously our only comment on clinical outcomes in the DVA cohort was the following:

Supplemental methods (previous revision)

Limitations include data on clinical outcomes in DVA. While data on recurrence has been reported for this cohort,[7] a substantial portion of this cohort received adjuvant hormonal or radiation therapy (**Supplemental table 3**) which would significantly bias results on tumor recurrence.

Reply

However, we would like to expand on these points for added clarity. In summary, we now substantially expand on the reasoning for not using the DVA cohort for survival outcome analyses.

Supplemental methods line 54 (current revision)

Limitations include data on clinical outcomes in DVA. While data on recurrence has been reported for this cohort,[7] the DVA cohort has several qualities that render it a more limited resource for time-to-event analyses in this study. This cohort is noticeably lower-risk relative to the JMHI. In JHMI 36% (107/300) of the cohort had high grade disease (grade group 4 or 5). In DVA only 12% (64/538) were high grade and thus the general risk of developing metastatic disease and death from prostate was very low. Accordingly,

only 5.4% (29/538) developed metastatic disease. This value is low relative to metastatic recurrence in JHMI (24%) and disease recurrence in TCGA (17.5%). Additionally, about 25% of men received hormonal therapy following surgery and nearly half received radiotherapy(**Supplemental table 3**). These additional treatments, their timing following surgery, and duration and dosing would influence clinical outcomes assessments in indeterminable ways. For instance, 60% of patients with tumors in the lowest quartile of plasma cell content received radiotherapy and 35% received hormonal therapy. These values were 45% and 25%, respectively, in patients with tumors in the highest quartile of plasma cell content. For these reasons, we did not include survival analyses for DVA.

2) The burden of neoantigens was calculated but no data are presented. This in fact could be quite informative and brief mention should be added as to how this varies by race. This is important when alluding to issues such as potential responsiveness to immune therapies.

Reply

Thank you for this important point. In the prior version of the manuscript we did present data on neoantigen and fraction genome altered(**Extended fig 2f-g**). We agree that added analysis looking at neoantigen and fraction genome altered by race would be informative and thus we have done the additional analysis and included it alongside the prior work (**Extended fig h-i**)

Our analyses did demonstrate that within prostate cancer neither neoantigen burden nor fraction genome altered correlated with plasma cells, IgG expression, NK activity, and now race. This work is consistent with the findings from melanoma and sarcoma as published recently in Nature (Cabrita et al. and Petitprez et al, respectively). In summary, we had demonstrated neoantigen burden and fraction genome altered did not correlate with plasma cells, IgG expression, or NK activity. We now also show no correlation with race.

Lines 185

Plasma cell content, IgG expression, and NK activity were all independent of tumor neoantigen burden and fraction genome altered in TCGA consistent with previous work on tumor plasma cell content and TLS in melanoma and sarcoma (**Extended figures 2f-g**).[8,9] Similarly, neoantigen burden and fraction genome altered did not correlate with race (**extended figures 2h-i**).

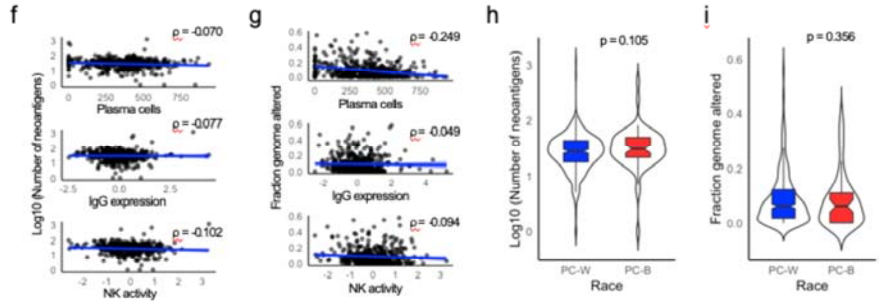

3) It should be made more clear that grade group one was excluded. This is an incredibly important subset of men particularly among those that have had PSA screening.

Reply

The reviewer is correct that patients with grade group 1 prostate cancer do comprise a very relevant patient group as it is the most commonly detected type of prostate cancer. Management of grade group 1 prostate cancer has undergone substantial modifications in the past several years as these cancers are now recognized to have minimal or no metastatic potential.[10] International guidelines now strongly endorse monitoring these cancers with programs referred to as active surveillance.[3–5]

Since grade group 1 prostate cancer is considered to be indolent, and surgery is not a preferred treatment, we excluded them from the JHMI cohort which was used as the primary source for survival analysis. Additionally, we previously noted there was disproportionate numbers of grade group 1 in JHMI based on race prior to creating the matched cohort (6 PC-W and 24 PC-B; **Methods**). Notably, men with grade group 1 prostate cancer were included in TCGA and DVA cohorts.

In summary, we now make this point clearer in the main text by updating the results, methods and supplemental methods sections.

Line 90

Utilizing a discovery cohort of intermediate- and high-grade primary PC from radical prostatectomy specimens grade- and stage-matched by race (Johns Hopkins Medical Institute [JHMI]; Black=150, [PC-B]; White=150, [PC-W]; **Supplementary table 1**) we assessed each tumor’s ability to resist lymphocyte infiltration using an established expression-based signature of tumor lymphocyte evasion (See Methods).

Line 247

To prepare for matching, 6 (1%) men were excluded due to missing data on pathological stage. All men with grade group 1 disease were excluded given the disproportionate numbers among Black men (24 vs 6; 30 total, 6%). Additionally, excluding men with grade group 1 PC would create a cohort more ideal for our analyses of outcomes following radical prostatectomy since the preferred treatment for most men with low grade PC is active surveillance with deferred treatment for signs of disease progression.[5] Finally, the cohort was randomly sorted before using the “matchit” function from the “Matchit” R package to derive a final grade- and stage-matched cohort of 300 men, 150 each of Black and White men. Race was defined by self-identification in this cohort.

Supplemental methods line 3

JHMI main strengths are related to clinical analyses which made this cohort our *a piori* main source for outcome analyses. This cohort was built excluding men with low grade (grade group 1) PC for whom the preferred treatment would be active surveillance as oppose to radical prostatectomy due to their low risk of developing metastatic disease and dying from PC and the disproportionate numbers of grade group 1 based on race prior to matching (6 PC-W and 24 PC-B).[5,11,12]

4) A simple grade group analysis is missing and one is left to wonder whether or not plasma cells and IgG correlate with grade. Certainly these data can be presented.

Reply

In the manuscript we performed a multivariable analysis to identify significant factors associated with metastatic recurrence. This multivariable analysis included important predictors of metastatic recurrence such as grade, stage, PSA, and genomic risk score. In these analyses, we show plasma cell content is a predictor of metastasis independent of these other factors, including grade.

Based on the reviewer comments, we now include a simple (univariable) grade group analysis. Within JHMI, there was no correlation between IgG or plasma cells and grade. In TCGA, a correlation was seen between higher grade tumors had lower plasma cell content.

In summary, we now show an analysis of grade group and plasma cell content and IgG expression.

Lines 162

Developing metastatic disease following local definitive treatment is a strong surrogate for overall survival for men with localized prostate cancer.[13] We found no association between metastasis-free and disease-free survival and CD8+ T-cell content. Conversely, increasing plasma cell content was associated with significantly prolonged metastasis-free and disease-free survival in JHMI and TCGA, respectively (**Figure 2a**). In JHMI, plasma cell content and IgG expression did not tend to vary by grade, while in TCGA lower grade tumors tended to have higher plasma cell content (Extended figure 2a-b) . In multivariable analysis including clinical covariates such as tumor grade, CD8+ T-cells remained non-significant while increased plasma cell content was independently associated with longer metastasis-free survival (**Figure 2b** and **extended figures 2c-d**).

Extended figure 2a-b

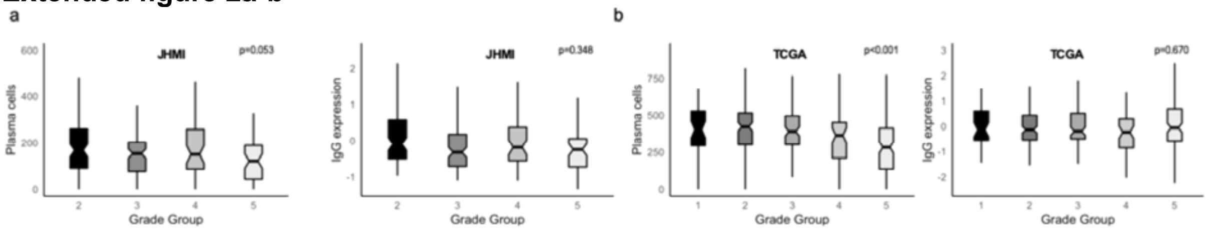

5) The distinction between plasma cell content and outcome is distinctly nonlinear in the JHMI and TCGA cohorts. This should be clearly pointed out as the current conclusion appears to be somewhat simplistic (especially when grade group one patients were excluded).

Reply

We agree with this point and have adjusted out discussion accordingly.

Line 228

Notably, a relationship between disease recurrence and plasma cell content was distinctly non-linear (**Figure 2a-b**) which should encourage future work determining a manner to measure and ascertain a clinically relevant cutoff for plasma cell content.

Reviewer #4 (Remarks to the Author):

1) the authors have done an excellent job with the revision of this paper and I am now in favour of publication as is

Reply

Thank you for taking the time to review our work.

References

1. Kweldam, C. F., Wildhagen, M. F., Bangma, C. H. & van Leenders, G. J. L. H. Disease-specific death and metastasis do not occur in patients with Gleason score ≤6 at radical prostatectomy. *BJU Int* **116**, 230–235 (2015).
2. Bianco, F. J. *et al.* Ten-Year Survival After Radical Prostatectomy: Specimen Gleason Score Is the Predictor in Organ-Confined Prostate Cancer. *Clinical Prostate Cancer* **1**, 242–247 (2003).
3. Parker, C. *et al.* Prostate cancer: ESMO Clinical Practice Guidelines for diagnosis, treatment and follow-up†. *Annals of Oncology* **31**, 1119–1134 (2020).
4. Chen, R. C. *et al.* Active Surveillance for the Management of Localized Prostate Cancer (Cancer Care Ontario Guideline): American Society of Clinical Oncology Clinical Practice Guideline Endorsement. *JCO* **34**, 2182–2190 (2016).
5. Mohler, J. L. *et al.* Prostate Cancer, Version 2.2019, NCCN Clinical Practice Guidelines in Oncology. *J Natl Compr Canc Netw* **17**, 479–505 (2019).
6. Ross, A. E. *et al.* Tissue-based Genomics Augments Post-prostatectomy Risk Stratification in a Natural History Cohort of Intermediate- and High-Risk Men. *European Urology* **69**, 157–165 (2016).
7. Howard, L. E. *et al.* Validation of a genomic classifier for prediction of metastasis and prostate cancer-specific mortality in African-American men following radical prostatectomy in an equal access healthcare setting. *Prostate Cancer and Prostatic Diseases* 1–10 (2019) doi:10.1038/s41391-019-0197-3.
8. Cabrita, R. *et al.* Tertiary lymphoid structures improve immunotherapy and survival in melanoma. *Nature* **577**, 561–565 (2020).
9. Petitprez, F. *et al.* B cells are associated with survival and immunotherapy response in sarcoma. *Nature* **577**, 556–560 (2020).

10. Hamdy, F. C. *et al.* 10-Year Outcomes after Monitoring, Surgery, or Radiotherapy for Localized Prostate Cancer. *New England Journal of Medicine* **375**, 1415–1424 (2016).

11. Klotz, L. *et al.* Long-Term Follow-Up of a Large Active Surveillance Cohort of Patients With Prostate Cancer. *JCO* **33**, 272–277 (2014).

12. Tosoian, J. J. *et al.* Intermediate and Longer-Term Outcomes From a Prospective Active-Surveillance Program for Favorable-Risk Prostate Cancer. *JCO* **33**, 3379–3385 (2015).

13. Xie, W. *et al.* Metastasis-Free Survival Is a Strong Surrogate of Overall Survival in Localized Prostate Cancer. *JCO* **35**, 3097–3104 (2017).

REVIEWERS' COMMENTS

Reviewer #3 (Remarks to the Author):

The responses to the reviewer are adequate. Thank you.
